# Substituents introduction of methyl and methoxy functional groups on resveratrol stabilizes mTOR binding for autophagic cell death induction

**DOI:** 10.1038/s41598-025-98616-6

**Published:** 2025-04-26

**Authors:** Zin Zin Ei, Satapat Racha, Preedakorn Chunhacha, Masashi Yokoya, Sohsuke Moriue, Hongbin Zou, Pithi Chanvorachote

**Affiliations:** 1 Department of Pharmacology and Physiology, Faculty of Pharmaceutical Sciences, Chulalongkorn university, Bangkok, 10330 Thailand; 2 Center of Excellence in Cancer Cell and Molecular Biology, Faculty of Pharmaceutical Sciences, Chulalongkorn University, Bangkok, 10330 Thailand; 3 Faculty of Pharmacy, Silpakorn University, Nakhon Pathom, Thailand; 4 Sustainable Environment Research Institute, Chulalongkorn University, Bangkok, Thailand; 5 Interdisciplinary Program in Pharmacology, Graduate School, Chulalongkorn university, Bangkok, 10330 Thailand; 6 Department of Biochemistry and Microbiology, Faculty of Pharmaceutical Sciences, Chulalongkorn University, Bangkok, Thailand; 7 Department of Pharmaceutical Chemistry, Meiji Pharmaceutical University, 2-522-1, Noshio, Kiyose, Tokyo, 204-8588 Japan; 8 College of Pharmaceutical Sciences, Zhejiang University, Hangzhou, 310058 China

**Keywords:** Autophagy, Stem cells, mTOR, Lung cancer, Resveratrol, SM-3, Computational biology and bioinformatics, Drug discovery

## Abstract

The regulation of the mammalian target of rapamycin (mTOR) protein by cancer cells can lead to uncontrol of cancer cell growth and cancer therapy resistance. The drug discovery of the anticancer agent 5-(3-hydroxy-4-methoxyphenethyl)-2-methoxy-3-methylphenol (SM-3), a derivative of resveratrol by substituting a methyl group at the hydroxy group of ring A and adding a methoxy group at the para position of ring B, shows promising potential for targeting autophagy to induce cell death and suppress cancer stem cells (CSCs) through the inhibition of the mTOR protein. In human lung cancer cells, SM-3 showed greater efficacy, with lower IC_50_ values of 72.74 ± 0.13, 67.66 ± 0.10, and 43.24 ± 0.11 µM in A549, H292, and H460 cells, respectively, compared to the parent compound, Resveratrol (Res). Moreover, the selectivity index (SI) values for BEAS2B cells compared to tumor cells treated with SM-3 were 10.99, 11.81, and 18.49 for A549, H292, and H460 cell lines, respectively. Therefore, SM-3 treatment led to reduced proliferation rates and colony formation in lung cancer cells. In our study, spheroids treated with SM-3 showed a higher proportion of dead spheroids compared to those treated with Res. Additionally, SM-3 treatment resulted in decreased expression of stem cell markers (CD133, CD44, and ALDH1A1) and transcription factors (OCT4, NANOG, and SOX2) in spheroids and organoids from human lung cancer cells by inhibiting the mTOR/pAkt pathway. SM-3 was also found to induce autophagic cell death, as indicated by Monodansylcadaverine staining, acidic vesicle formation, and the conversion of LC3BI to LC3BII. Using MM/GBSA calculations, SM-3 exhibited a stronger binding affinity (-25.09 kcal/mol) compared to Res (-18.85 kcal/mol). SM-3 also displayed greater stability during the entire simulation, maintaining lower RMSD values of 2–3 Å even after 80 ns. In summary, the introduction of methyl and methoxy functional groups on Res to create SM-3 effectively suppressed cancer spheroids and organoids formation in lung cancer cells by targeting the upstream mTOR/pAkt pathway.

**Supplementary Information:**

The online version contains supplementary material available at 10.1038/s41598-025-98616-6.

## Introduction

Lung cancer continues to be a major cause of cancer-related deaths globally, with survival rates remaining low despite advances in medical treatment^1^. The main challenges are cancer recurrence and drug resistance. New approaches to cancer therapy aim to target the fundamental mechanisms driving tumor growth and resistance^2–4^. Current research suggests that targeted therapy, which involves the use of drugs designed to act on specific molecular targets, can enhance treatment outcomes for lung cancer patients. The mTOR protein is crucial in regulating cellular metabolism and is frequently hyperactivated in many cancers, particularly lung cancer^5,6^. It serves as a key regulator of cell growth, proliferation, metastasis, and survival^7–9^. Therefore, targeting the mTOR has become a promising strategy in cancer therapy, as inhibiting its activity can curb cancer cell proliferation and trigger apoptosis.

The mTOR functions through two distinct complexes: mTOR complex 1 (mTORC1) and mTOR complex 2 (mTORC2). mTORC1 directly regulates autophagy, a process known to induce cell death in cancer cells that have developed resistance to chemotherapy^5,10^. Autophagy is a survival mechanism that enables cells to adapt to stress; however, excessive or uncontrolled autophagy can lead to cell death^11,12^. This occurs when the self-digestion process becomes overly active, causing the breakdown of vital cellular components. As a result, targeting autophagy has emerged as a potential therapeutic approach for inducing cell death in resistant cancer cells^13–15^. Numerous studies have demonstrated that autophagy and apoptosis can work in tandem in cancer treatment^16,17^. mTORC2 is crucial for cell survival, metabolism, proliferation, and cytoskeleton organization. It regulates cell survival by influencing the Akt phosphorylation pathway. mTORC2-mediated Akt-Ser473 phosphorylation plays a critical role in regulating cell survival pathways. Inhibiting key survival proteins like Akt and mTOR is a promising cancer treatment strategy, as this pathway is often dysregulated in cancers, including lung cancer^18^. Alterations in upstream proteins, such as the epidermal growth factor receptor (EGFR), can activate the mTOR/Akt pathway, leading to increased cancer cell survival by inhibiting apoptosis. Research indicates that inhibiting the mTOR/Akt pathway can induce cancer cell death and slow tumor progression in vivo^19^. Therefore, targeting mTOR and Akt is crucial for lung cancer therapy, and compounds that promote apoptosis and autophagy by inhibiting the PI3 K/Akt/mTOR pathway show potential as promising therapeutic agents^20^. Inhibiting mTOR signaling may effectively eliminate CSCs, which are involved in tumor heterogeneity and contribute to drug resistance, metastasis, tumor formation, and recurrence. Targeting mTOR reduces the CSCs population by disrupting their self-renewal and inducing apoptosis^21^. Although cancer immunotherapy has advanced significantly in the past decade, challenges like low response rates, side effects, and cost-effectiveness remain. Emerging experimental evidence suggests that therapies targeting CSCs could reduce metastasis and minimize the risk of cancer recurrence^22,23^.

Lung cancer clinical samples show elevated CD133 levels, a CSC marker associated with poor prognosis and therapy resistance. The CD133-high subpopulation demonstrates self-renewal and tumorigenic potential in vitro and in vivo^24^. This phenotype is linked to the overexpression of stemness-regulating transcription factors, including OCT4, NANOG, and SOX2^25^. Furthermore, CD44, a transmembrane glycoprotein, is essential for CSCs functions such as self-renewal, apoptosis resistance, migration, differentiation, and proliferation within the cancer microenvironment. CD44 is also recognized as a tumor-initiating marker in lung cancer cells, supported by both in vitro and in vivo studies^26^.

In this study, compounds derived from natural products have demonstrated potential for inhibiting tumor growth, metastasis, and cancer stemness. One such compound is Res (trans-3,5,4′-trihydroxystilbene), a natural polyphenol known for its strong antioxidant properties. Res may effectively induce autophagy by directly inhibiting the mTOR pathway in various type of cancer^27^. It has been reported to target mTOR inhibition in various cancers, including oral, breast, and lung cancer. However, Res has certain limitations, including low water solubility, susceptibility to degradation, and poor bioavailability^28,29^. In our research, modifying the structure of Res by substituting a methyl group on the hydroxy group of ring A and introducing a methoxy group at the para position on ring B resulted in the SM-3 compound, which may enhance the anticancer properties of Res The resulting SM-3 compound can improve bioavailability, slow down rapid metabolism, and increase lipophilicity compared to Res^30,31^. A previous study demonstrated that the Res derivative, Moscatilin, is more effective than Res in inhibiting cancer stem cells in lung cancer^32^. However, the therapeutic anticancer activity of Res derivatives like SM-3, particularly their role in autophagy and effects on 3D organoid stem cells via inhibition of the mTOR pathways, remains unclear.

The effects of SM-3 on mTOR and its underlying molecular mechanisms of action have not yet been explored. This study aims to investigate the molecular interaction between SM-3 and mTOR using computational approaches. The analysis includes drug-likeness evaluation, PAINS screening, molecular docking, molecular dynamics (MD) simulations, and free energy calculations to characterize protein–ligand interactions and identify compounds with drug-like properties. Furthermore, our findings indicate that SM-3 induces autophagy in lung cancer cells and suppresses self-renewal in spheroids and organoids formation by inhibiting the mTOR pathway.

## Results

### Synthesis of Res derivatives

The synthesis of bibenzyl compounds with various substituents can be easily achieved from aromatic aldehydes bearing the planned substituents. The compounds evaluated in this study (SM-1 ~ 3, 8) were synthesized by condensing substituted aromatic aldehydes **1c** and **1 d** with four Horner-Wadsworth-Emmons reagents (**3a ~ d**) to form E -stilbenes, followed by a hydrogenation reaction.

Aldehyde **1 d**was synthesized from 3-methylcatechol in a three-step process^33^. After conversion to compound **5** by regioselective benzyl protection, **1 d** was obtained through formylation and methyl etherification. The synthesis of the Horner-Wadsworth-Emmons reagents **3a** and **3c**has already been reported^34^. The synthesis of **3b** and **3 d** was carried out from isovanillin and **1 d**, respectively—aromatic aldehydes with the corresponding substituents—following the same method used for **3a** and **3c**.

**Fig. 1:**
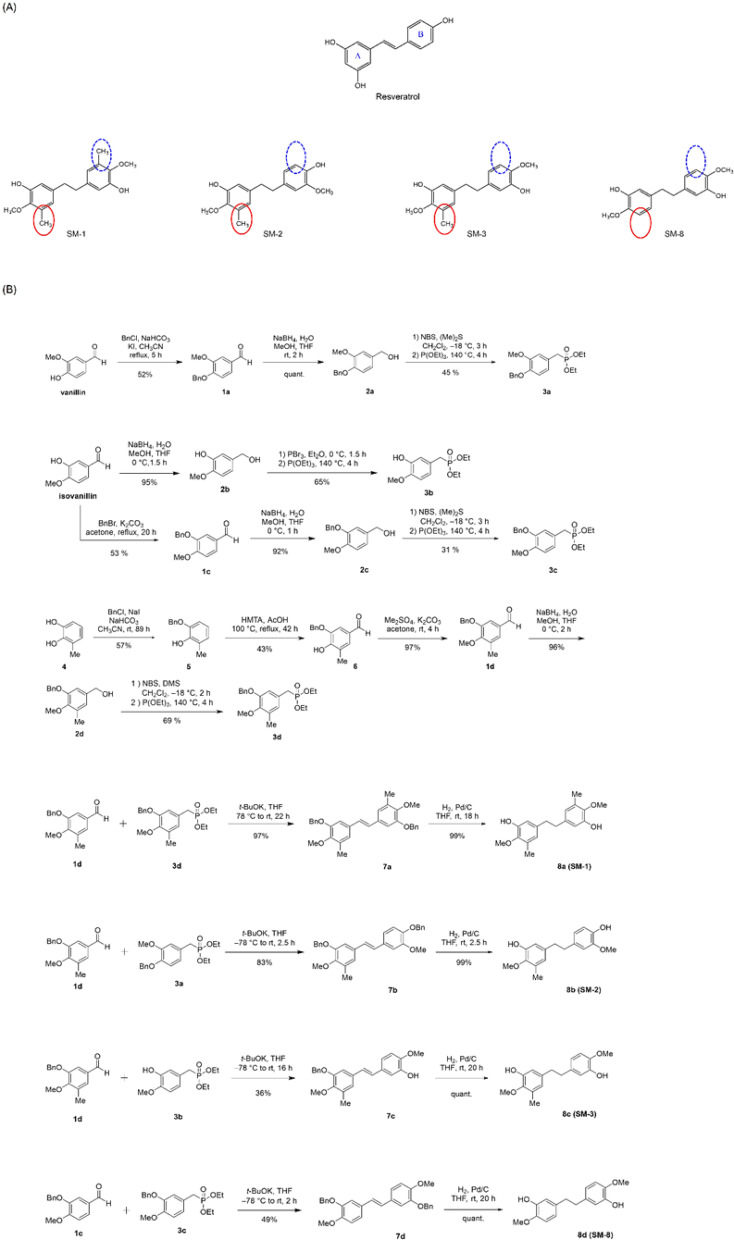
Derivatives of Res − 5,5’-(ethane-1,2-diyl)bis(2-methoxy-3-methylphenol) (SM-1), S5-(4-hydroxy-3-methoxyphenethyl)−2-methoxy-3-methylphenol (SM-2), 5-(3-hydroxy-4-methoxyphenethyl)−2-methoxy-3-methylphenol (SM-3), and 5,5’-(ethane-1,2-diyl)bis(2-methoxyphenol) (SM-8). (**A**) Structures of Res, SM-1, SM-2, SM-3, and SM-8. (**B**) Step by step synthesis for Res derivatives-SM-1, SM-2, SM-3, and SM-8.

E-stilbene derivatives **7a ~ d** was obtained by the condensation reaction of the synthesized aromatic aldehydes **1c** and **1 d** with the four Horner-Wadsworth-Emmons reagents (**3a ~ d**). Finally, the planned bibenzyl compounds (SM-1 ~ 3, 8) were obtained by catalytic hydrogenation of the alkenes in good yields (Fig. 1).

### Screening of Res derivatives induce cytotoxicity, and apoptosis in NSCLC cells

**Fig. 2:**
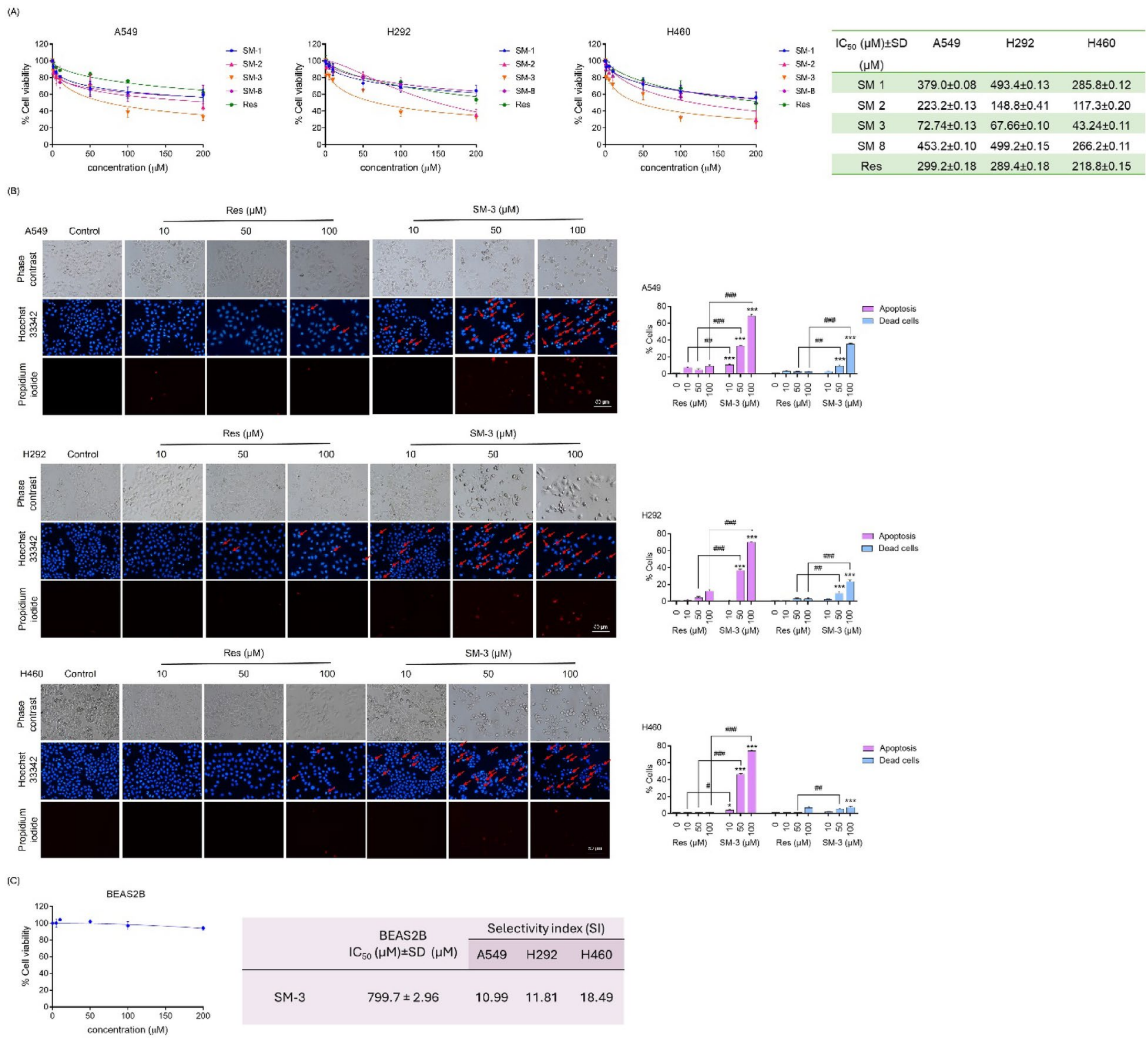
Effect of Res Derivatives on Cell Viability and Apoptosis in NSCLCs (A549, H292, and H460). (**A**) Lung cancer cells were cultured and treated with Res derivatives (0–200 µM) for 24 h. An MTT assay was conducted to determine the IC_50_ values for the Res derivatives (SM-1, SM-2, SM-3, and SM-8) used in treating the human lung cancer cells, with the parent Res compound serving as a positive control. (**B**) NSCLC cells were seeded and exposed to various doses of Res and SM-3 for 24 h. Apoptosis and cell death were assessed by co-staining with Hoechst 33,342 and propidium iodide (PI). Images were captured using a fluorescence microscope, and the percentages of apoptotic and dead cells were calculated. Scale bar: 50 μm (magnification: 20x) (**C**) The non-tumorigenic epithelial cell line derived from human bronchial epithelial cells (BEAS2B) was seeded and treated with SM-3 (0–200 µM) for 24 h. An MTT assay was performed to determine the IC_50_ values for SM-3 in the non-tumorigenic BEAS2B cell line. The selectivity index (SI) for lung cancer cells was then calculated. Data are presented as mean ± SD (*n* = 3), with significance indicated as ***p < 0.001 compared to untreated control cells, ^##^*p* < 0.01, ^###^*p* < 0.001 compared to Res-treated NSCLC cells.

First, a cell viability assay was conducted to evaluate the cytotoxicity of Res derivatives compared to the parent compound, Res. Various modified Res derivatives were used to treat non-small cell lung cancer (NSCLC) cells (A549, H292, and H460) at different concentrations (0–200 µM) for 24 h. The results showed that the SM-3 compound had a markedly stronger cytotoxic effect on these lung cancer cell lines compared to the original Res compound. Among the Res derivatives, SM-3 demonstrated the highest efficacy in the A549, H292, and H460 cell lines, with IC_50_ values of 72.74 ± 0.13, 67.66 ± 0.10, and 43.24 ± 0.11 µM, respectively. In contrast, the IC_50_ values for the parent Res compound were 299.2 ± 0.18, 289 ± 0.18, and 218.8 ± 0.15 µM in the A549, H292, and H460 cell lines, respectively. Additionally, the IC_50_ values for SM-1, SM-2, and SM-8 ranged from approximately 200 to 500 µM, which are generally higher than those of the parent Res compound (Fig. 2A).

The effects of Res and SM-3 on apoptosis and necrosis in lung cancer cells were assessed using Hoechst 33,342 and propidium iodide (PI) staining to identify apoptotic and necrotic cells. SM-3 treatment led to significantly more apoptotic cells compared to Res, showing characteristic changes like chromatin condensation and nuclear fragmentation across lung cancer cell lines. In contrast, Res treatment did not cause nuclear fragmentation (Fig. 2B). Overall, SM-3 was more effective than Res in reducing cell viability in lung cancer cells.

To assess whether SM-3 selectively targets lung cancer cells, normal human lung epithelial BEAS2B cells were treated with the same concentration of SM-3 to calculate the selectivity index (SI). The IC_50_ value for SM-3 in BEAS2B cells was 799.7 ± 2.96 µM. The SI values for BEAS2B cells compared to tumor cells treated with SM-3 were 10.99, 11.81, and 18.49 for A549, H292, and H460 cell lines, respectively (Fig. 2C). A selectivity index (SI) greater than 1.0 suggests that the compound is more effective in targeting lung cancer cells compared to normal cells.

Various concentrations of SM-3 (0–200 µM) were treated to normal human lung epithelial BEAS2B cells for 48 h and 72 h. The results indicated that the IC_50_ values for SM-3 in BEAS2B cells were 349.0 ± 0.43 µM at 48 h and 206.9 ± 0.53 µM at 72 h (Fig. S1). These values were higher than those observed in SM-3 treated lung cancer cells at 24 h, which were 72.74 ± 0.13 µM for A549, 67.66 ± 0.10 µM for H292, and 43.24 ± 0.11 µM for H460. Therefore, SM-3 demonstrated greater efficacy and selectivity, suggesting a favorable safety profile for normal human lung epithelial BEAS2B cells.

### Res derivatives, SM-3 inhibits proliferation and decreases colony formation that compare with parent compound, Res in human lung cancer cells

**Fig. 3:**
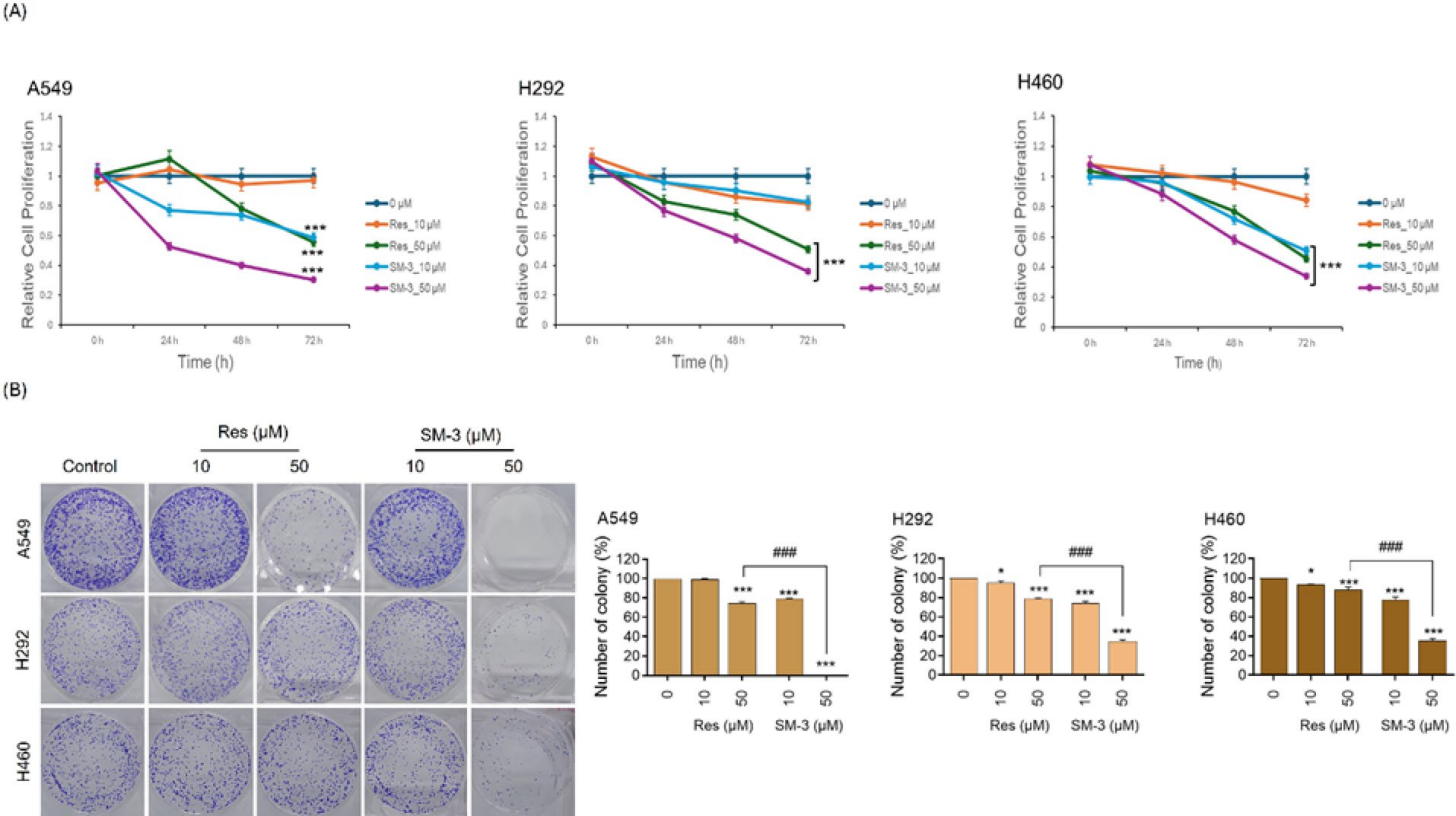
Effects of SM-3 on antiproliferation and colony formation inhibition in NSCLC cells (A549, H292, and H460) were examined, with Res serving as a positive control. (**A**) The impact of Res and SM-3 (0–50 µM) on lung cancer cell proliferation was assessed using an MTT assay over 24, 48, and 72 h, and results were analyzed relative to the control group at 0 h. (**B**) Lung cancer cells were seeded and treated with 0–50 µM of Res and SM-3 for 24 h. Following treatment, the cells were incubated for an additional 7 days, stained with crystal violet, and the number of colonies was counted. Data are presented as mean ± SD (*n* = 3). Significance is indicated as *p < 0.05, **p < 0.01, ***p < 0.001 compared to untreated control cells, and ^###^*p* < 0.001 compared to Res-treated NSCLC cells.

To determine the antiproliferative effect of SM-3, the study explored various concentrations ranging from 0 to 50 µM. Lung cancer cells were cultured in a growth medium with or without SM-3 for 72 h, and an MTT assay was conducted every 24 h. The proliferation assay revealed that A549 cells treated with SM-3 showed a decreased proliferation rate starting at 24 h and continuing through 72 h at a dose of 10 µM, compared to Res. In contrast, treating A549 cells with 10 µM of Res had no impact on their proliferation. Additionally, H292 cells treated with Res did not show any effect at doses of 10 and 50 µM. However, treatment with SM-3 resulted in a significant reduction in proliferation, beginning at 24 h with doses of 10 and 50 µM. At the later time points of 48 and 72 h, SM-3 treated cells exhibited a notable reduction in proliferation compared to those treated with Res. The SM-3 treatment at a dose of 10 µM significantly reduced the proliferation rate of H460 cells, beginning at 48 h to 72 h, compared to Res. In contrast, 10 µM of Res showed no effect on the proliferation of H460 cells (Fig. 3A).

The colony formation assay was employed to evaluate the effectiveness of Res and SM-3 for assessing cell survival, based on the principle that a single cancer cell can grow into a colony. After treating NSCLC cells (A549, H292, and H460) with Res and SM-3 (at concentrations of 0, 10, and 50 µM) for 24 h, the drugs were removed, and the cells were allowed to culture for an additional 7 days to facilitate colony growth. SM-3 inhibited colony formation in all cell lines in a dose-dependent manner, while Res had no impact on colony formation. Crystal violet staining showed a significantly higher inhibition rate in A549, H292, and H460 cells treated with 50 µM of SM-3 (Fig. 3B). Overall, these results indicate the promising anti-proliferative and anti-cancer effects of SM-3 on lung cancer cells.

### Lipinski and PAINS filters

Table S1 shows that the resveratrol and SM-3 compounds lack potential PAINS liabilities and comply with Lipinski’s rules, making them promising candidates for the early stages of drug discovery. These results suggest that the compounds are unlikely to produce false positives in laboratory-based assays, supporting their potential for further development.

### Molecular docking

**Fig. 4:**
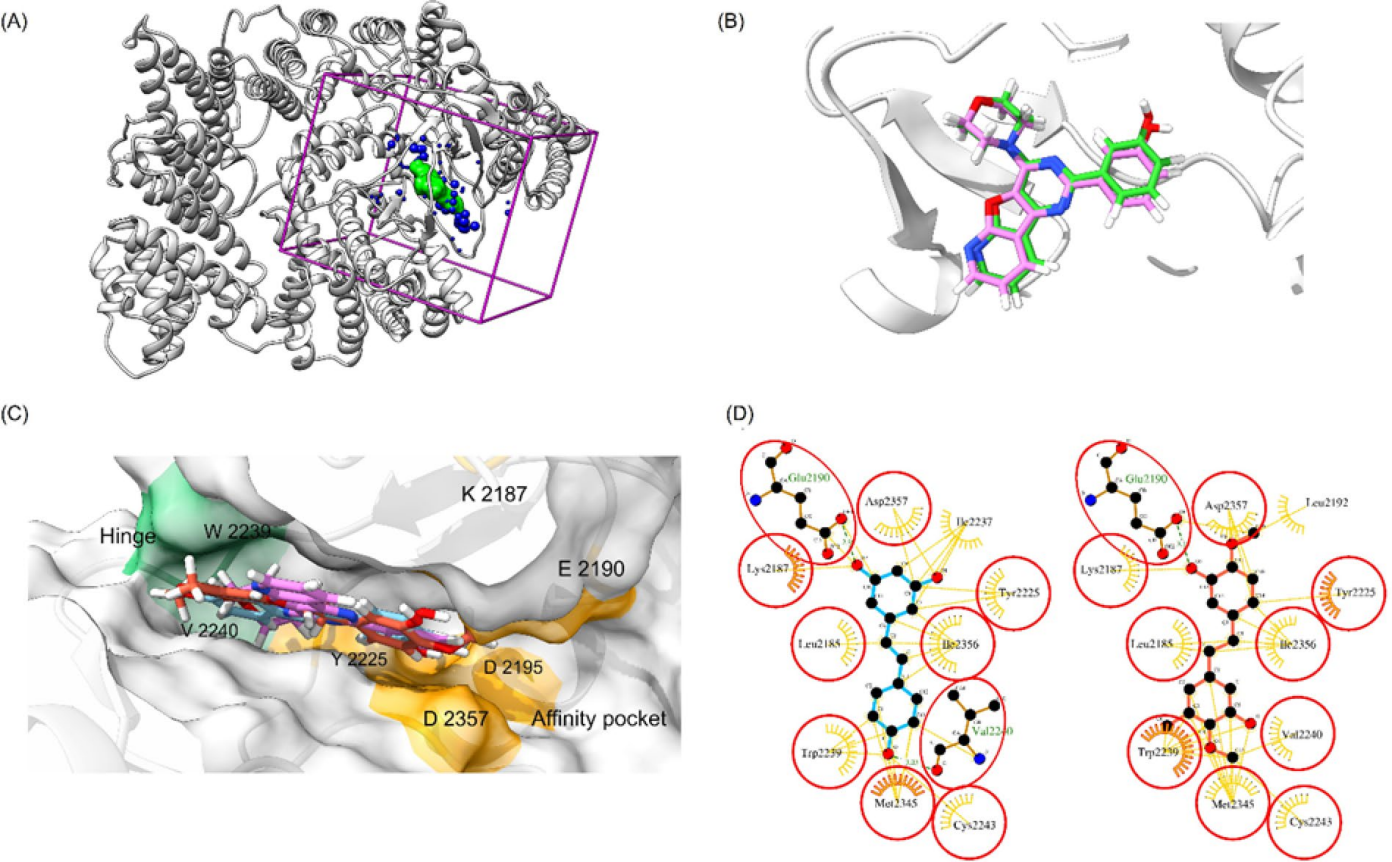
(**A**) Docking setup of kinase mTOR (PDB code 4 JT6). (**B**) Superimposition of co-crystallized ligand (green) and docked ligand PI-103 (pink). (**C**) Superimposed binding structures of PI-103 (pink), Res (blue), and SM-3 (red) in the ATP binding site of mTOR. (**D**) Comparison of docked poses between Res (blue) and SM-3 (red).

The docking workflow was validated by assessing its performance in redocking a co-crystallized ligand (Fig. 4A and B). The results showed an RMSD value of 0.485 Å, and the redocking indicated a binding mode similar to that of the co-crystallized ligand (PDB code: 4 JT6), confirming the reliability of the docking protocol.

Res has been reported to inhibit both mTORC1 and mTORC2 by docking into the ATP-binding site of mTOR^35^. To further investigate the binding effects of the Res analogue SM-3, we conducted modeling studies to reveal its binding modes within the ATP-binding site of mTOR. In molecular docking simulations, SM-3 demonstrated a stronger binding affinity (−34.02 kcal/mol) compared to Res (−25.16 kcal/mol). Visualization of the compounds’ binding poses (Fig. 4C and D) shows that both are effectively contained within the ATP-binding site of mTOR, interacting with critical residues in the hinge region (Val2240) and affinity pocket (Lys2187, Glu2190, Tyr2225, and Asp2357)^36^. Additionally, key interactions with Trp2239 contribute to SM-3’s activity and specificity for mTOR over PI3 Ks^36^. Based on the predicted binding affinities and binding poses, these compounds were selected for further MD simulations to assess their stability, followed by extensive calculations using AMBER18.

### MD simulations and free energy calculations

Due to the inherent limitations of molecular docking, many protocols tend to ignore or oversimplify the role of water molecules and solvent effects, despite their critical importance in protein-ligand interactions. Initial recognition interactions between the compound and protein may shift under dynamic conditions. To more accurately evaluate the stability of the predicted consensus pose, we conducted a 100 ns MD simulation of the ligand-mTOR complex.

**Fig. 5:**
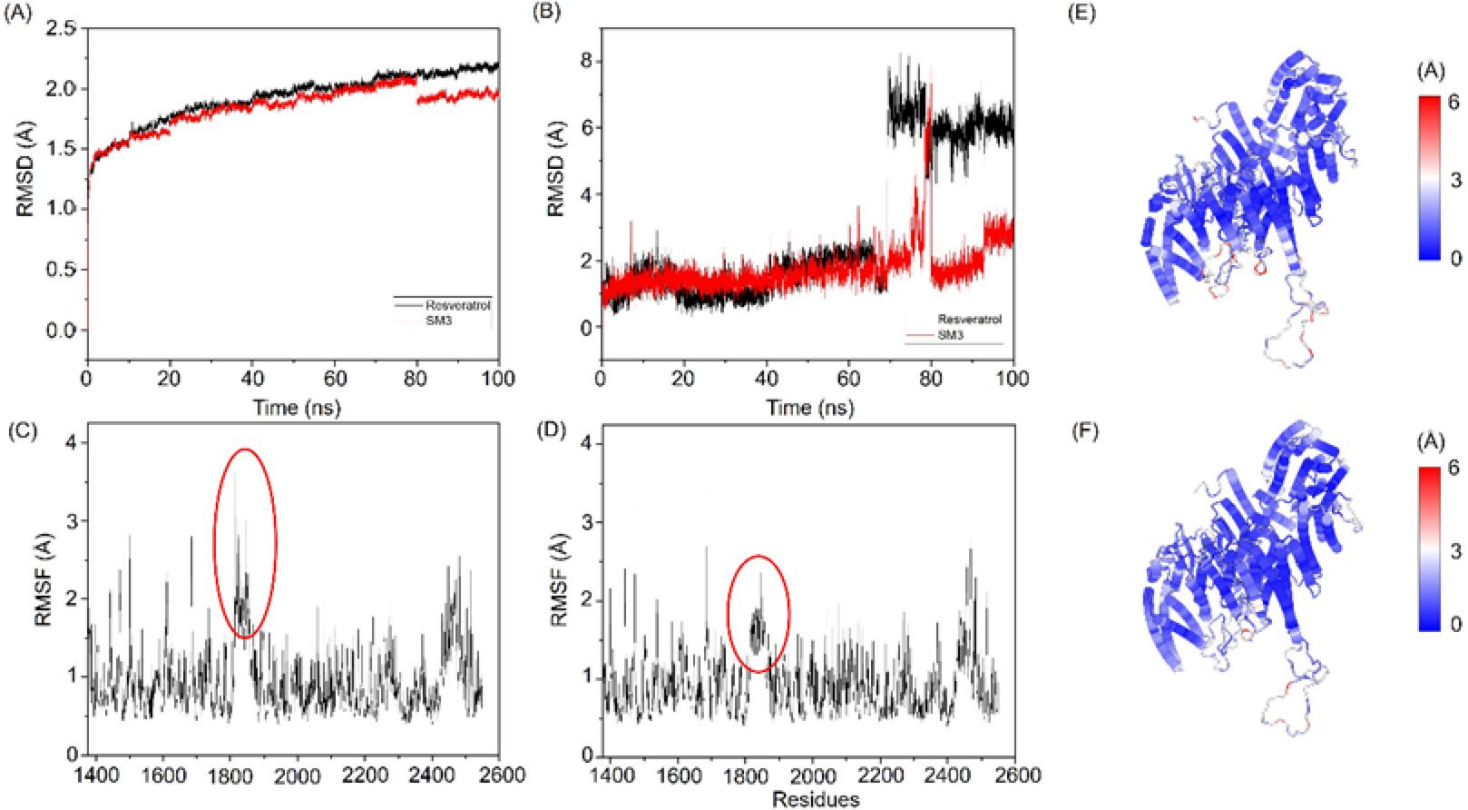
(**A**) Root Mean Square Deviation (RMSD) of the ligand-protein complexes. (**B**) Ligand-only RMSD. (**C**) RMSF profile of the mTOR–Res complex. (**D**) RMSF profile of the mTOR–SM-3 complex. (**E**) C-alpha RMSD of the final structure for the mTOR–Res complex. (**F**) C-alpha RMSD of the final structure for the mTOR–SM-3 complex.

As shown in Fig. 5A, the RMSD of the SM-3 and Res complex indicates that the system reaches equilibrium around 80 ns from the start of the MD simulation. By the end of the simulation, the RMSD of the Res complex stabilizes at approximately 2.2 Å, while the SM-3 complex achieves a slightly lower RMSD value of about 1.8 Å. The ligand RMSDs, as plotted in Fig. 5B, reflect the stability of the ligands within the protein’s binding pocket. During the first 60 ns, both Res and SM-3 demonstrate relatively stable behavior, with RMSD values fluctuating between 1 and 2 Å, indicating that they maintain conformations close to their initial binding positions. However, a significant change occurs around the 70–80 ns mark, during which Res experiences a major conformational shift; its RMSD value increases sharply to 6–8 Å and remains elevated for the remainder of the simulation. In contrast, SM-3 exhibits greater stability throughout the entire simulation period, maintaining lower RMSD values of 2–3 Å even after 80 ns. This suggests that SM-3 remains close to its original binding pocket throughout the simulation. Generally, a low RMSD indicates that the ligand maintains a stable, well-defined orientation within the active site. The RMSD profiles of SM-3 and Res show a significant contrast. This suggests that SM-3 has a more stable binding mode compared to Res, which undergoes substantial repositioning in the binding site. The consistent and lower RMSD values of SM-3 indicate that it may have a more favorable binding interaction with the target protein.

The RMSF values offer insights into the mobility and flexibility of residues within a protein structure. RMSF analysis of the mTOR complexes with Res and SM-3 (Fig. 5C and D) revealed similar profiles, indicating comparable flexibility across most residues. Notably, residues 1800–1850 in the mTOR–SM-3 system exhibited lower RMSF values, suggesting reduced mobility in this region, likely due to strong binding interactions between mTOR and SM-3. This observation highlights a potential stabilizing effect of SM-3 on the protein-ligand complex, which could influence its functional dynamics.

The C-alpha RMSD analysis, comparing the protein structure from the initial docking pose to the final MD simulation, provides insights into the conformational stability of the mTOR complexes (Fig. 5E and F). The mTOR–SM-3 system exhibited lower RMSD values, indicating minimal structural rearrangement and a stable conformation. In contrast, higher RMSD values for the mTOR–Res system suggest significant conformational changes, potentially reflecting increased flexibility or reduced stability.

**Fig. 6:**
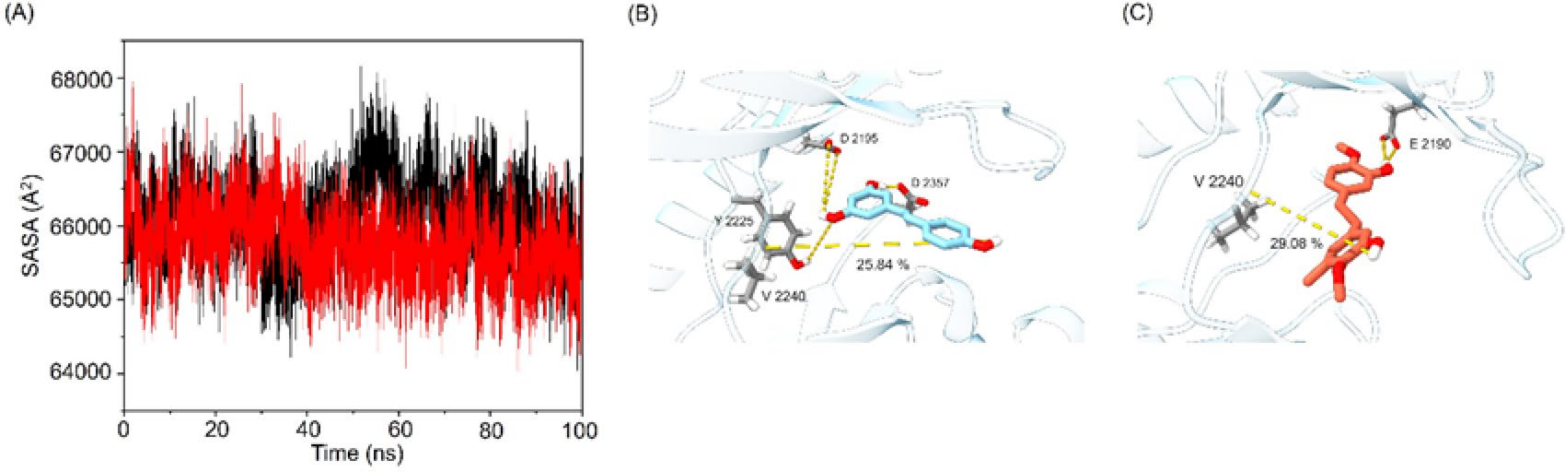
(**A**) SASA analysis of the mTOR–Res (black) and mTOR–SM-3 (red) systems. (**B**) Binding conformation of Res and (**C**) binding conformation of SM-3 in complex with mTOR, as observed in the final structure of the MD simulation. Yellow dashed lines indicate hydrogen bonds.

The expansion of protein volume varied across the different systems and was quantified by the solvent-accessible surface area (SASA), which represents the fraction of a protein accessible to the solvent. An increase in SASA correlates with a rise in protein volume, while minimal variation during the simulation period indicates structural stability. The binding of small molecules may influence SASA, potentially inducing conformational changes in the protein. The average SASA values for the mTOR–Res and mTOR–SM-3 systems were determined to be 66,093 Å² and 65,759 Å², respectively. Notably, the higher SASA value of the mTOR–Res system suggests greater exposure and potential instability. In contrast, the lower SASA value observed for the mTOR–SM-3 system indicates relatively higher stability and compactness (Fig. 6A).

Hydrogen bonds are essential for stabilizing mTOR–ligand complexes and facilitating specific interactions between the receptor and ligands. To assess these interactions, a hydrogen bond lifetime analysis was conducted during a 100 ns MD simulation (Table S2). The analysis revealed that SM-3 demonstrated a higher percentage of interactions with Val2240 compared to Res (Fig. 6B and C). These results indicate that the mTOR–SM-3 complex exhibits greater stability and rigidity than the mTOR–Res complex, likely due to the involvement of Val2240 as a key residue.

**Fig. 7:**
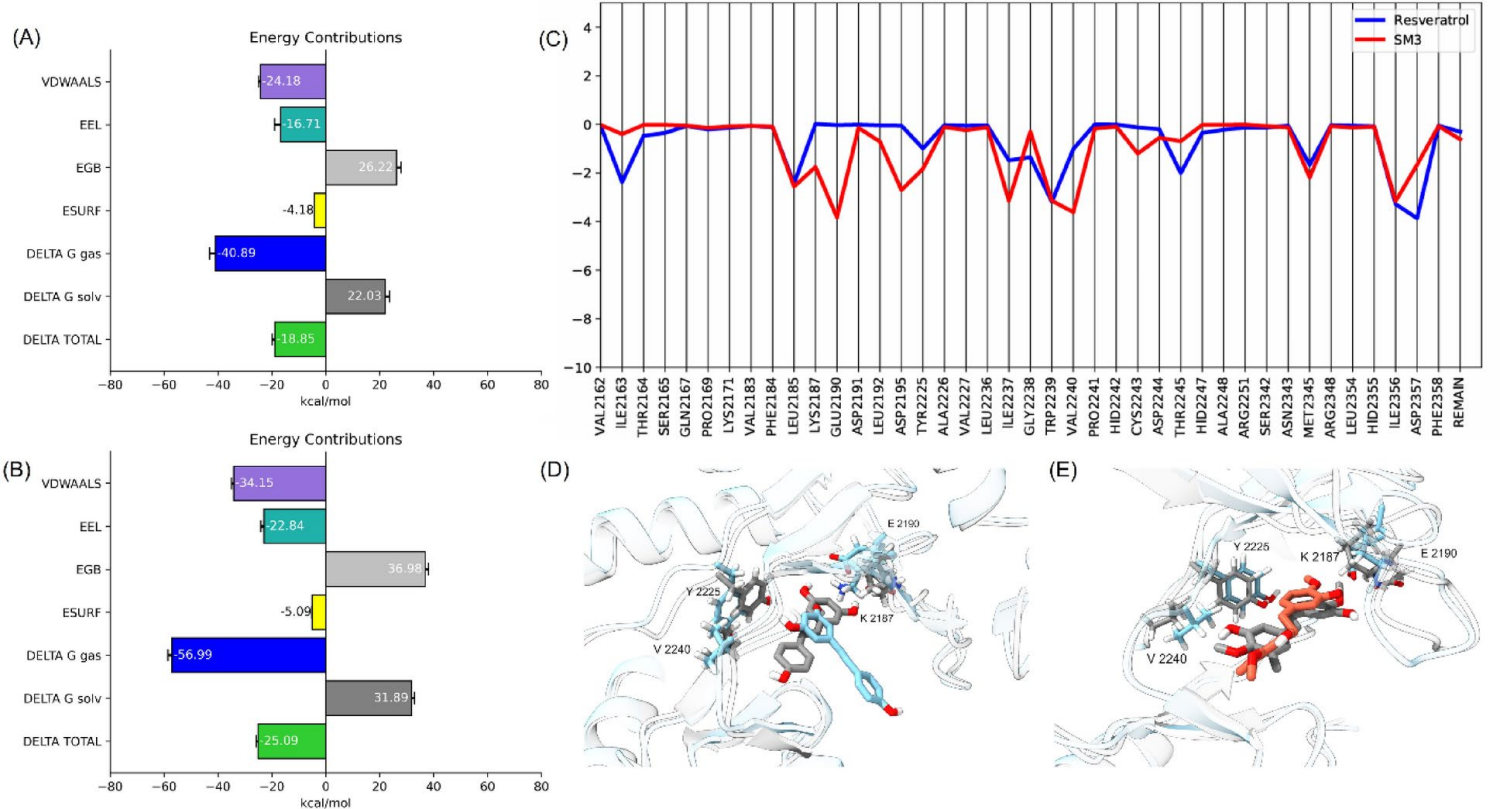
(**A**) MM/GBSA of Res. (**B**) MM/GBSA of SM-3. (**C**) Energy decomposition analysis. (**D**) Superimposition of the pre-MD (grey) and post-MD (blue) of Res complex. (**E**) Superimposition of the pre-MD (grey) and post-MD (red) of SM-3 complex.

The binding energy analysis using MM/GBSA highlights the superior binding affinity of SM-3 compared to Res, with total values of −25.09 kcal/mol and − 18.85 kcal/mol, respectively. This distinction underscores SM-3’s stronger van der Waals and electrostatic interactions (Fig. 7A and B), which contribute to its stable association within the mTOR binding site. These interactions not only mitigate the desolvation penalty upon binding but also enhance SM-3’s potential as a favorable candidate for mTOR inhibition.

The energy decomposition analysis (Fig. 7C) provides further insight into the molecular basis of SM-3’s binding affinity, identifying Glu2190, Trp2239, Val2240, and Ile2356 as critical residues that contribute significantly to stabilizing the complex. The interaction with Val2240 within the hinge region is particularly noteworthy, as this region is crucial for mTOR inhibition^37^, suggesting that SM3 effectively targets a mechanistically significant site. These stable interactions likely underlie SM3’s ability to maintain its binding pose throughout the simulation, ensuring effective engagement with key residues (Fig. 7E).

Conversely, Res’s inability to sustain interactions with key residues, including Val2240, Lys2187, Glu2190, and Tyr2225, reflects its limited binding potential (Fig. 7D). The observed instability, attributed to Res’s higher structural mobility as indicated by its ligand RMSD profile, results in weaker binding interactions and eventual displacement from the binding site. This instability highlights the limitations of Res as an mTOR inhibitor and underscores the importance of robust interactions with critical residues for effective binding.

These findings position SM-3 as a promising candidate for mTOR inhibition, given its stable interactions within the hinge region and affinity pocket of the ATP binding site. Additionally, the insights gained from the energy decomposition analysis could guide the design of SM-3 derivatives with optimized interactions for enhanced inhibitory potency.

### mTOR targeting of SM-3 treated human lung cancer cells

mTOR protein can drive tumor growth and progression through various mechanisms, such as enhancing growth factor receptor signaling, promoting angiogenesis, supporting glycolytic and lipid metabolism, facilitating cancer cell migration (metastasis), and inhibiting autophagy. Targeting mTOR inhibition presents a promising approach to addressing the challenges of treating human lung cancer^37,38^.

**Fig. 8:**
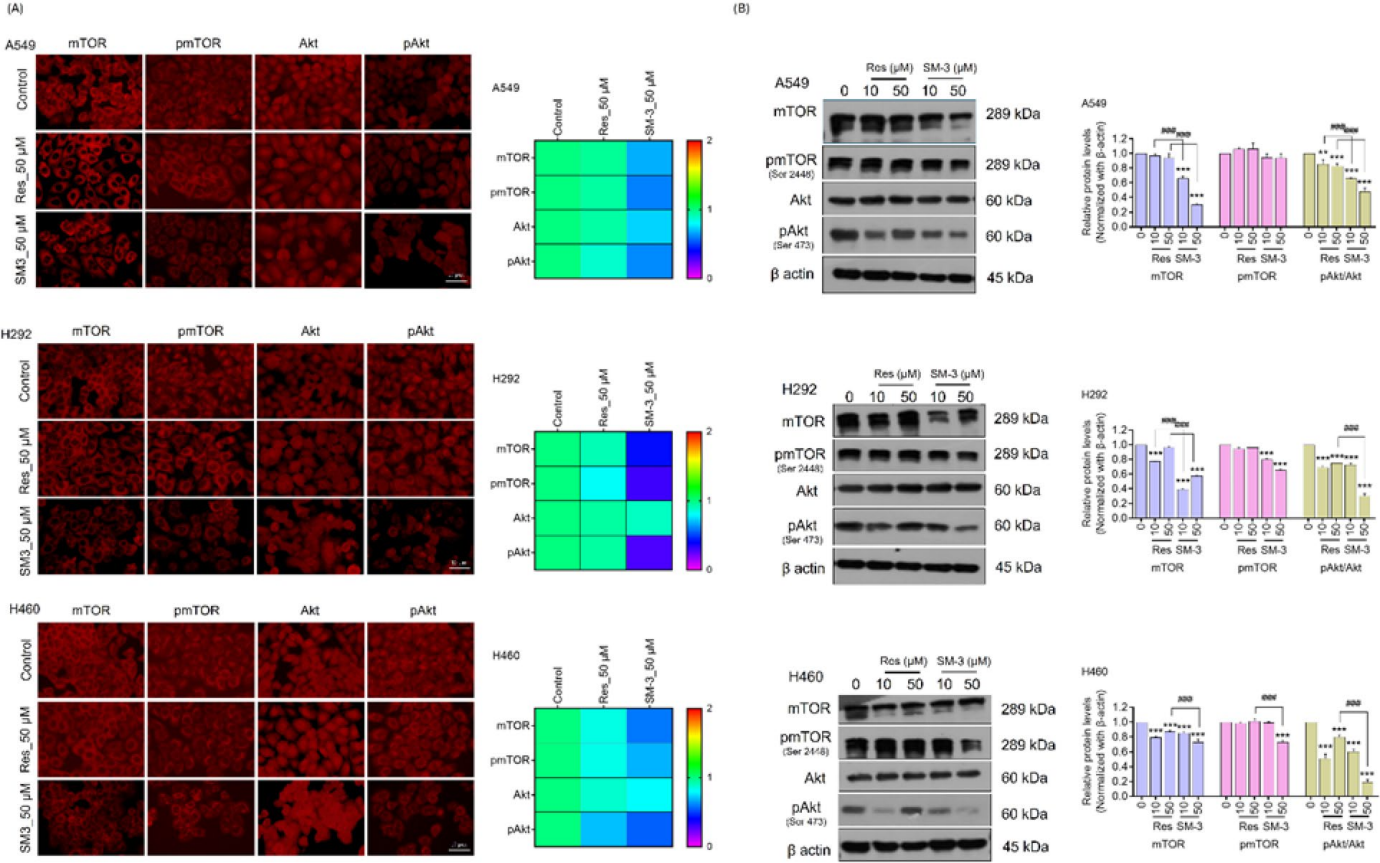
mTOR targeting in SM-3-treated NSCLC cells (A549, H292, and H460) was evaluated. The cancer cells were exposed to varying concentrations of SM-3 (0–50 µM) for 24 h. (**A**) Levels of mTOR, p-mTOR (Ser2448), Akt, and p-Akt (Ser473) were measured through immunofluorescence analysis, with fluorescence intensity quantified using ImageJ software. Scale bar: 10 μm (magnification: 40x) (**B**) The protein expression of mTOR, p-mTOR (Ser2448), Akt, and p-Akt (Ser473) was assessed by western blotting, with β-actin used to confirm equal protein loading. Blots were analyzed by densitometry using ImageJ. Data are presented as mean ± SD (*n* = 3). Significance is indicated as *p < 0.05, **p < 0.01, ***p < 0.001 versus untreated control cells and ^###^*p* < 0.001 compared to Res-treated NSCLC cells.

The protein levels of Akt and mTOR were analyzed in lung cancer cells treated with SM-3 and Res using immunofluorescence. Treatment with SM-3 led to a significant decrease in mTOR, pmTOR (Ser2448), and pAkt (Ser473) in A549, H292, and H460 cells compared to those treated with the parent compound, Res (Fig. 8A).

Human lung cancer cells were treated with SM-3 and Res for 24 h, and the targeted proteins were analyzed using western blot analysis. The results showed that SM-3-treated lung cancer cells exhibited reduced expression of mTOR and its phosphorylated form (Ser2448) compared to those treated with Res. Additionally, a significant decrease in the p-Akt (Ser473)/Akt protein levels was observed in NSCLC cells treated with 50 µM of SM-3 after 24 h, compared to the Res-treated cells. These findings suggest that the Res derivative SM-3 effectively inhibits mTOR, making it a potential treatment for NSCLCs (Fig. 8B).

Inside cells, mTOR assembles into two catalytic complexes: mTORC1 and mTORC2. mTORC1 mainly controls cell metabolism, including autophagy, and growth, whereas mTORC2 plays a key role in cell survival, stemness, and proliferation. In cancer cells, the mTOR/Akt signaling pathway is frequently overactivated, contributing to tumor development and progression^7–9^.

Based on previous findings, SM-3 can modulate the mTOR pathway, leading to a significant reduction in the NSCLC cell population. To validate the effects of SM-3 on mTOR, rapamycin, an allosteric inhibitor of mTORC1, was used. Our results demonstrated that rapamycin pretreatment enhanced the cytotoxic effects of SM-3, as evidenced by a significant increase in cytotoxicity compared to SM-3 treatment alone (Fig. S2A).

Furthermore, the impact of rapamycin pretreatment on SM-3-induced apoptosis and necrosis in lung cancer cells was assessed using Hoechst 33,342 and propidium iodide (PI) staining to distinguish apoptotic and necrotic cells. NSCLC cells pretreated with rapamycin before SM-3 exposure exhibited a significantly higher number of apoptotic cells compared to SM-3 alone, characterized by chromatin condensation and nuclear fragmentation across lung cancer cell lines (Fig. S2B).

For further confirmation, NSCLC cells were pretreated with rapamycin (0.2 µM) for 1 h, followed by SM-3 (50 µM) treatment for 24 h, and key target proteins were analyzed using western blotting. The results indicated that rapamycin pretreatment combined with SM-3 resulted in a lower expression of mTOR and its phosphorylated form (Ser2448) compared to treatment with SM-3 alone (Fig. S2C).

Overall, these findings confirm that SM-3 effectively inhibits mTOR, highlighting its potential as a therapeutic agent for NSCLC.

### Suppressive effects of SM-3 on CSC-enriched spheroids and targeting CSC markers via mTOR Inhibition during spheroids and organoids formation in human lung cancer cells

**Fig. 9:**
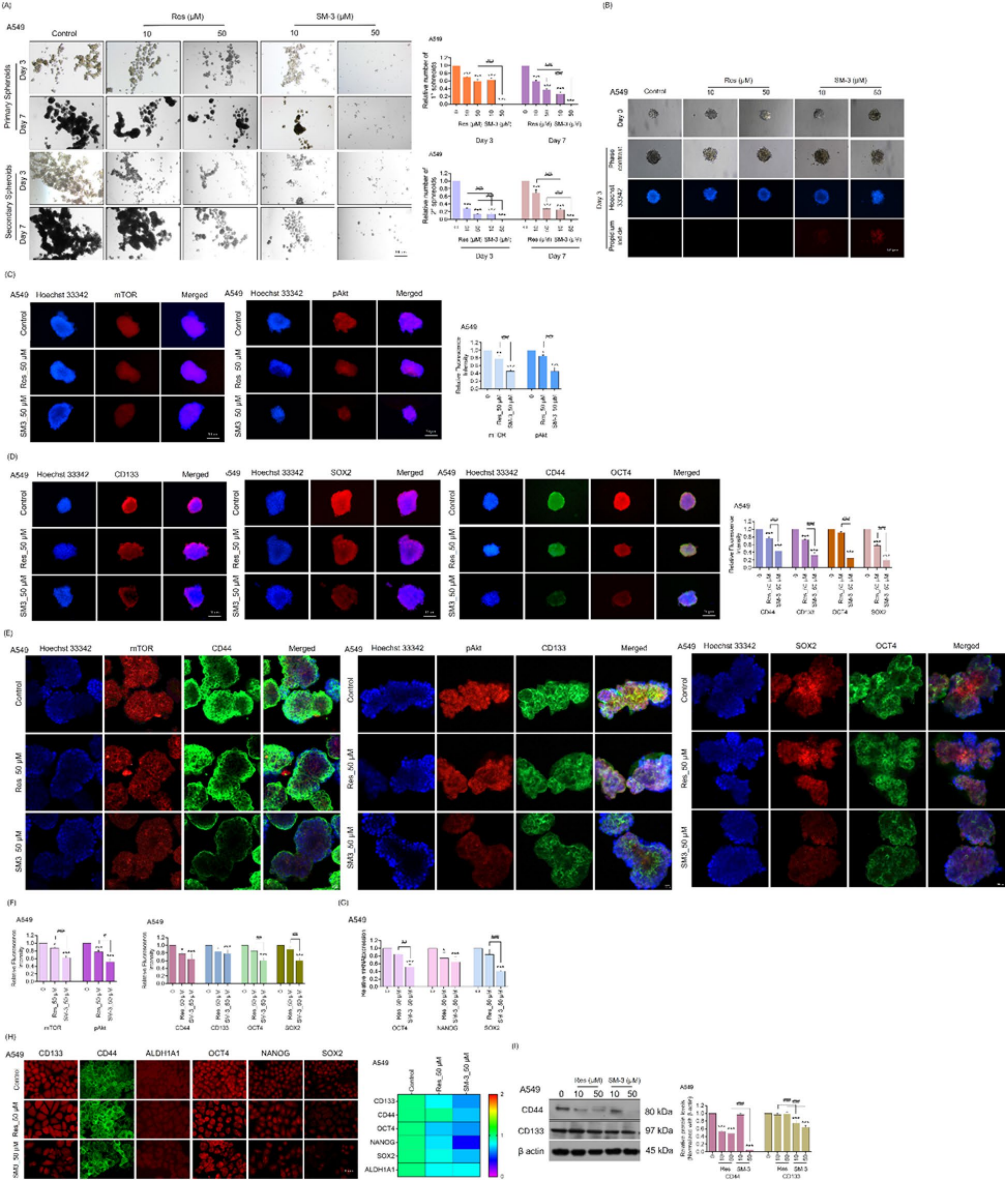
Suppressive effects of SM-3 on CSCs markers via mTOR inhibition during spheroids and organoids formation in A549 cells. (**A**) The A549 cells were treated with SM-3 (50 µM) and observed using a phase-contrast microscope after 3 and 7 days of incubated primary and secondary spheroids. The relative number of spheroids were analyzed using ImageJ software. Scale bar: 100 μm (magnification: 4x) (**B**) Single spheroids from a CSCs-rich population of A549 cells were treated with SM-3 (50 µM) for 3 days, and CSCs viability was evaluated using Hoechst 33,342/PI double staining. Scale bar: 50 μm (magnification: 20x) (**C**, **D**) The CSCs-rich spheroids were treated with SM-3 (50 µM) for 24 h. The levels of upstream proteins mTOR and pAkt in SM-3 treated CSCs-rich spheroids were assessed using immunofluorescence. The SM-3 treated A549 spheroids were examined for the expression of stem cell markers, as well as the transcription factors. Scale bar: 50 μm (magnification: 20x) (**E**) SM-3 targeted CSCs by inhibiting mTOR during 3D organoids formation in A549 cells, as analyzed by immunofluorescence. The expression levels of pAkt (Ser473), mTOR, CSCs-rich transcription factors (CD133 and CD44), and stem cell markers (OCT4 and SOX2) were measured in SM-3-treated organoids. Scale bar: 20 μm (magnification: 40x) by confocal microscope (**F**) The fluorescence intensity was measured by image J software. (**G**) Cells were treated with SM-3 (50 µM), and the mRNA expression levels of the stem cell transcription factors OCT4, NANOG, and SOX2 were measured. The mRNA levels were normalized against the housekeeping gene GAPDH, and relative mRNA expression was calculated using comparative Ct cycles. (**H**) The heat map displays the fluorescence intensity of stem cell markers and stem cell transcription factors analyzed by using Image J software. Scale bar: 10 μm (magnification: 40x) (I) The protein expression levels of stem cell markers were assessed using western blot analysis, with the blot reprobed for β-actin to ensure equal protein loading. The parent compound Res (50 µM) served as the positive control. Data are presented as mean ± SD (*n* = 3). Significance is indicated as *p < 0.05, **p < 0.01, ***p < 0.001 compared to untreated control cells, and ^#^*p* < 0.05, ^##^*p* < 0.01, ^###^*p* < 0.001 compared to Res-treated A549 cells.

CSCs are critical targets for developing new anticancer agents. The study examined the effects on lung cancer CSCs by enriching the CSCs subpopulation and analyzing stemness traits like self-renewal and differentiation in CSCs-enriched spheroids cultured under detachment conditions. Lung cancer cells were initially treated with 50 µM of Res or SM-3 and cultured to form primary spheroids over 7 days in ultra-low attachment plates. These primary spheroids were then reseeded to develop secondary spheroids over a period of 7 days. After 7 days incubation, the spheroids formation was evaluated. The results indicated that untreated cells had a strong capacity for spheroid formation, whereas SM-3-treated spheroids showed a reduced ability to form lung cancer spheroids compared to those treated with the parent compound, Res (Fig. 9A).

The ability of SM-3 to suppress CSCs-like phenotypes and induce apoptosis in CSCs populations was assessed using a three-dimensional (3D) spheroid formation assay, followed by double staining with Hoechst 33,342 and PI. CSCs-enriched lung cancer cell populations (secondary spheroids) were generated as described in the methods section, and individual spheroids were isolated in a 96-well ultra-low attachment plate and treated with Res and SM-3 (50 µM) for 3 days. The effects on CSCs-rich spheroids were monitored at 0 h, 24 h, 48 h, and 72 h. After 72 h of treatment, the spheroids were costained with Hoechst 33,342 and PI. The spheroid formation assay revealed that SM-3-treated spheroids showed a significant reduction in size and viability (indicated by PI staining) compared to those treated with Res (Fig. 9B).

SM-3-treated spheroids exhibited decreased viability in forming H292 and H460 spheroids compared to those treated with the parent compound, Res (Fig. S3A, S3B, S4 A, S4B).

CSCs represent promising drug targets, providing a potentially effective strategy for cancer treatment. In our research, the CSCs spheroids of A549, H292, and H460 cells were treated with 50 µM of SM-3, and the expression levels of CSCs markers (CD133 and CD44) as well as stem cell transcription factors (OCT4 and SOX2) were analyzed using immunofluorescence. Figure 9D indicates that treatment with SM-3 resulted in a significant reduction in the levels of CD133, CD44, SOX2, and OCT4, suggesting a depletion of stem cell phenotypes. For comparison, Res was used as a positive control, and the results demonstrated that Res had a lesser impact on the CSCs population.

Moreover, the high levels of mTORC2 promote cancer stemness, which contributes to cell survival and cancer cell proliferation^21^. Consequently, mTOR and pAkt levels were assessed in 3D spheroid cells enriched with CSCs. The 3D CSCs-rich population was treated with 50 µM of SM-3 and Res for 24 h, and the expressions of mTOR and pAkt were evaluated using an immunofluorescence assay. The results indicated that SM-3 treated spheroid cells exhibited a greater reduction in mTOR and pAkt expression compared to Res-treated spheroids across all cancer cell lines (A549, H292, and H460) (Fig. 9C).

To verify the inhibition of CSCs in lung cancer spheroids through the mTOR/pAkt pathway, we will conduct additional experiments using organoids. Organoids are formed through in vitro 3D culture and can replicate the structure and physiological functions of organs or tissues in vivo. As a 3D cell culture model, organoids closely resemble human organs in both structure and function, exhibiting characteristics such as cell proliferation and differentiation, self-renewal, self-assembly, long-term culture, and genetic stability^39^. In our study, we utilized matrigel to produce organoids from lung cancer cells to evaluate the anticancer activity of the SM-3 compound. The resulting organoid population was treated with Res and SM-3 (50 µM) for 24 h, after which we assessed the mTOR/pAkt pathway, stem cell markers (CD44 and CD133), and stem cell transcription factors (SOX2 and OCT4) using immunofluorescence analysis (Fig. 9D).

The results showed that the population of 3D organoids treated with SM-3 exhibited a significant decrease in pAkt/Akt and pmTOR/mTOR levels compared to the Res-treated organoid cultures. This suggests that SM-3 has promising anticancer activity, primarily targeting the inhibition of the mTOR protein.

mTOR is essential for promoting stem cell differentiation, facilitating the growth and proliferation of stem and progenitor cells, and influencing the differentiation pathways of multipotent stem cell populations in cancer cells^40^. Treatment of the 3D organoid cell population with SM-3 resulted in a significant reduction in the levels of stem cell markers (CD133 and CD44) and stem cell transcription factors (OCT-4 and SOX2) in lung cancer organoids (Fig. 9E and F).

SM-3-treated spheroids demonstrated lower levels of stem cell markers and transcription factors in spheroids and organoids of H292 and H460 through mTOR inhibition, compared to the parent compound, Res (Fig. S3C-F, S4 C-F).

To further validate the suppression of CSCs, we examined whether SM-3 could reduce the expression of stem cell-related transcription factors. The mRNA expression levels of stemness transcription factors were analyzed using the real-time RT-qPCR method. Our findings revealed that in A549 cells treated with SM-3 at a concentration of 50 µM, the mRNA expression levels of OCT4, NANOG, and SOX2 decreased significantly to 0.4-fold, 0.5-fold, and 0.25-fold, respectively (Fig. 9G). Likewise, the mRNA results showed that SM-3 substantially reduced the levels of the transcription factors OCT4, NANOG, and SOX2 compared to Res-treated lung cancer cells, including H292 and H460 cells (Fig. S3G, S4G).

Subsequently, the protein expression levels of stem cell markers and transcription factors were assessed. A549, H292, and H460 cells were treated with SM-3 (50 µM) for 24 h, after which the expression levels of stem cell markers (CD44, CD133, and ALDH1 A1) and stem cell transcription factors (NANOG, OCT4, and SOX2) were analyzed using immunofluorescence assays. The heatmap shown in Fig. 9H, S3H, S4H demonstrates that treatment with SM-3 led to a significant decrease in the levels of stem cell markers and transcription factors in A549, H292, and H460 cells compared to lung cancer cells treated with the parent compound, Res. Thus, SM-3, a derivative of Res, functions as a targeted therapy for stem cells in lung cancer cells.

A549, H292, and H460 cells were treated with 50 µM of SM-3 for 24 h, and the expression levels of stem cell markers CD133 and CD44 were analyzed using Western blot analysis. Figure 9I, S3I, S4I illustrates that SM-3 resulted in a significant reduction of the CSCs markers CD133 and CD44 in all three cell lines. These results indicate that SM-3 may effectively target stem cells in human lung cancer.

Thus, these findings indicate that SM-3 demonstrates a more significant inhibitory effect on the CSCs population in lung cancer cells. In summary, SM-3 exhibits greater potency in suppressing CSCs by targeting the mTOR/pAkt pathway.

### Effect SM-3 on mTOR’s role in Akt regulation and the impact of LY294002 on stemness and organoids formation

The mTOR target plays a crucial role in regulating Akt. We added the experiment indicating that LY294002 (Akt inhibitor) can suppress the stemness as well as organoids formation.

The lung cancer cells were initially pretreated with LY294002 (5 µM) for 1 h with 50 µM for SM-3 24 h cultured to form primary spheroids over 7 days in ultra-low attachment plates. These primary spheroids were then reseeded to develop secondary spheroids over a period of 7 days. After 7 days incubation, the spheroids formation was evaluated. The results indicated that LY294002 with SM-3-treated spheroids showed a reduced ability to form lung cancer spheroids compared to those treated with SM-3 alone treated spheroids (Fig S5A, S6 A, S7 A).

SM-3 suppressed CSC-like phenotypes and induced apoptosis in CSC-enriched lung cancer spheroids, as assessed by a 3D spheroid formation assay and Hoechst 33,342/PI staining. Secondary spheroids were treated LY294002 with SM-3-treated spheroids SM-3 (50 µM) for 72 h. LY294002 with SM-3-treated spheroids significantly reduced spheroid size and viability compared to SM-3 alone treated spheroids (Fig S5B, S6B, S7B).

An experiment our drug SM-3 with LY294002 (Akt inhibitor) demonstrated its ability to suppress stemness and organoids formation than SM-3 alone treatment. In our finding, the CSCs spheroids of A549, H292, and H460 cells were pretreated with LY294002 (5 µM) for 1 h with SM-3 (50 µM) for 24 h, and the expression levels of pAkt, CSCs markers (CD133 and CD44) as well as stem cell transcription factors (OCT4 and SOX2) were analyzed using immunofluorescence. Fig. S5C, S6 C, S7 C indicates that LY294002 pretreatment with SM-3 population showed in a significant reduction in the levels of pAkt, CD44, CD133, OCT4, and SOX2, suggesting a depletion of stem cell phenotypes than SM-3 treated alone.

Additional organoids experiments will be conducted to verify CSCs inhibition in lung cancer organoids via the mTOR/pAkt pathway. Matrigel-based lung cancer organoids were used to evaluate SM-3’s anticancer activity. Organoids pretreated with LY294002 (5 µM) for 1 h with SM-3 (50 µM) for 24 h were analyzed for mTOR/pAkt pathway activity, stem cell markers (CD44, CD133), and transcription factors (SOX2, OCT4) via immunofluorescence. LY294002 with SM-3 treated organoids were significantly reduced pAkt, stem cells transcription factors, and stemness markers compared to SM-3 treated alone, suggesting its potential to suppress stemness in lung cancer organoids (Fig. S5D, S6D, S7D).

The expression levels of stem cell markers (CD44, CD133, ALDH1 A1) and transcription factors (NANOG, OCT4, SOX2) were subsequently evaluated in A549, H292, and H460 cells were pretreated with LY294002 (5 µM) for 1 h with SM-3 (50 µM) for 24 h, followed by immunofluorescence analysis. As shown in the heatmap (Fig. S5E, S6E, S7E), LY294002 pretreatment with SM-3 population were significantly reduced these markers compared to cells treated with SM-3 alone.

Additionally, western blot analysis (Fig. S5F, S6 F, S7 F) confirmed a marked reduction in CSC markers CD133 and CD44 across all three cell lines after treatment with LY294002 (5 µM) for 1 h with SM-3 (50 µM) for 24 h.

According to our confirmation experiments, our interesting drug SM-3 effectively targets CSCs population in lung cancer via inhibiting mTOR/Akt pathway.

### SM-3 triggers autophagy-related cell death and modifies the levels of autophagy-related proteins in human lung cancer cells

Autophagy is the mechanism through which cells degrade and recycle proteins and organelles to preserve intracellular balance. Typically, autophagy serves a protective function within cells; however, when autophagy processes are disrupted or when there is excessive autophagic flux, it can result in cell death^11–13^. In lung cancer cells, autophagy-mediated cell death is a significant therapeutic target. Most anti-cancer drugs primarily aim to enhance the formation of autophagosomes.

**Fig. 10:**
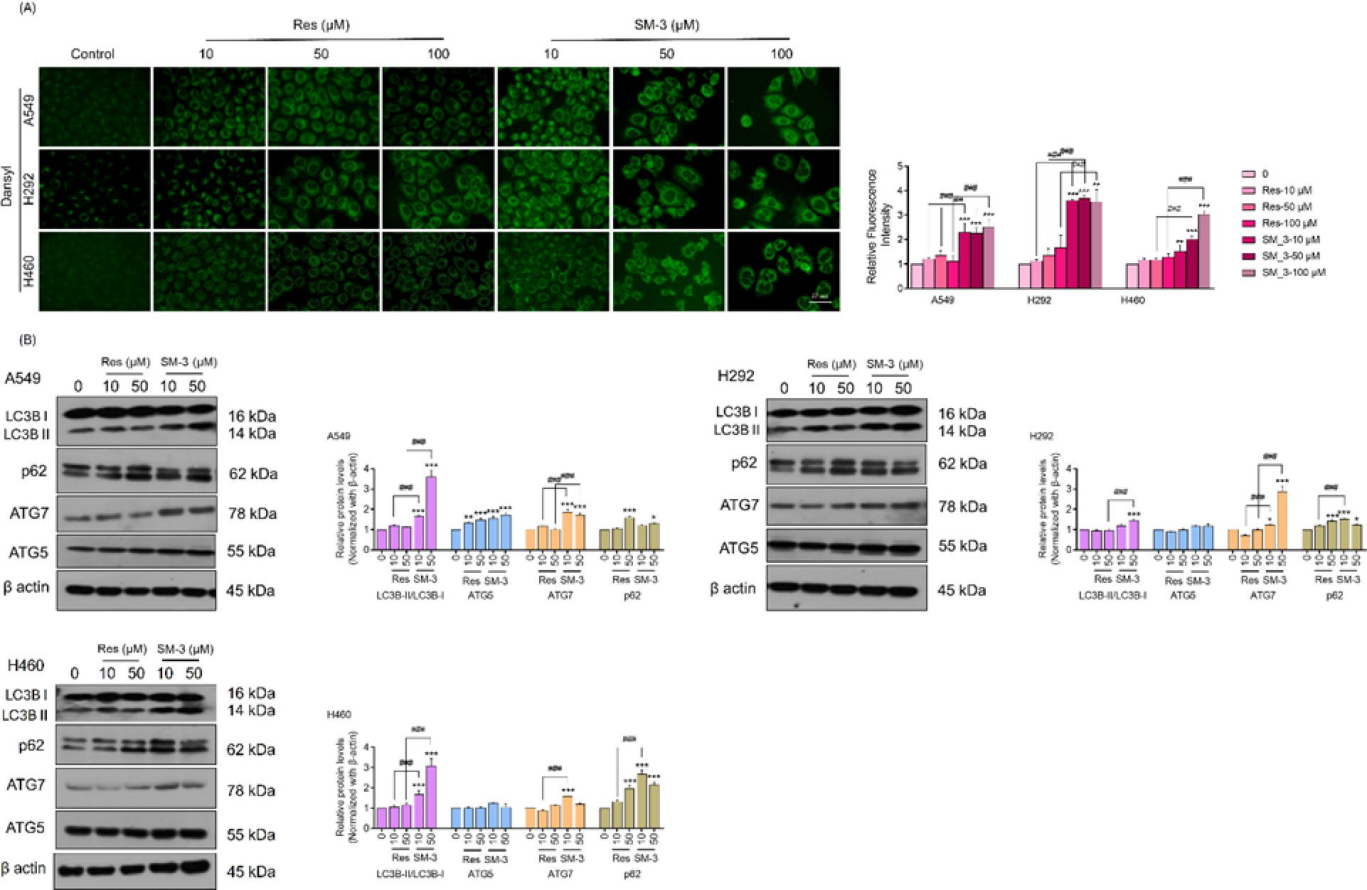
Induction of autophagy-related cell death and changes in autophagy-related proteins were observed in human lung cancer cells treated with SM-3. (**A**) Human lung cancer cells were treated with concentrations ranging from 0 to 100 µM of SM-3 and stained with monodansylcadaverine. The stained autophagic vacuoles in the treated cells were visualized using fluorescence microscopy (Olympus IX51 with DP70). Scale bar: 10 μm (magnification: 40x). (**B**) The levels of autophagy-related proteins in SM-3 treated human lung cancer cells were measured using western blot analysis. This analysis detected the protein levels of LC3BII, SQSTM1/p62, ATG5, and ATG7. The membrane was reprobed with β-actin to ensure equal protein loading. Data are presented as mean ± SD (*n* = 3). Significance is indicated as *p < 0.05, **p < 0.01, ***p < 0.001 compared to untreated control cells, and ^#^*p* < 0.05, ^##^*p* < 0.01, ^###^*p* < 0.001 compared to Res-treated NSCLCs.

Monodansylcadaverine serves as a crucial fluorescent marker for autophagic vacuoles within cells. Following treatment with SM-3, the formation of autolysosomes in cancer cells was stained using Monodansylcadaverine. The findings showed that the bright green fluorescence of the autophagic vacuoles was more prominent in lung cancer cells treated with SM-3 compared to those treated with Res (Fig. 10A).

Subsequently, the autophagic induction in SM-3 treated cells was verified through western blot analysis of autophagy-related protein markers, including LC3BI/II, SQSTM1/p62, ATG5, and ATG7. Notably, LC3B-II is a key marker for the formation of autophagosomes, produced by the conjugation of cytosolic LC3B-I with phosphatidylethanolamine (PE) on the surfaces of newly formed autophagosomes. Our results indicate that the formation of LC3B-II is significantly higher in lung cancer cells treated with SM-3 compared to those treated with the parent compound, Res. This suggests that SM-3 primarily promotes the increase in autophagosome formation.

The proteins SQSTM1/p62, ATG5, and ATG7 are involved in the formation of phagophores, promoting their elongation. The results indicated that the expression of ATG7 and ATG5 proteins was elevated in lung cancer cells treated with SM-3 compared to those treated with Res. Additionally, the levels of the scavenger protein p62 were significantly higher in SM-3 treated lung cancer cells than in untreated cells. The p62 protein can bind to both ubiquitin and LC3, thus targeting autophagosomes and aiding in the clearance of ubiquitinated proteins. Overall, in lung cancer cells treated with SM-3, there was a significant conversion of LC3B-I to LC3B-II, along with increased levels of ATG7, ATG5, and p62 (Fig. 10B).

### Effects of SM-3 on mitochondrial membrane potential and mitochondrial function on NSCLC that compare to lead compound, Res

The mitochondrial metabolic activity of living cells for assessing reduction of a non-fluorescent dye, resazurin into a brightly fluorescent pink dye called resorufin by resazurin reduction assay^41^. This assay determined the primary function of mitochondrial respiratory chain within the cells. SM-3 and Res were used to treat NSCLC (A549, H292, and H460) at different concentrations (0–200 µM) for 24 h. The results showed that the SM-3 compound had markedly lower mitochondrial activity and cell viability on these lung cancer cell lines compared to the parent Res compound (Fig. S8A).

The abnormalities of mitochondrial function and integrity can lead to a variety of intracellular signaling cascades, oxidative stress and the initiation of programmed cell death^42^. Therefore, the detection of mitochondrial abnormalities is crucial for SM-3 treated NSCLC cells. The mitochondrial abnormalities were determined for mitochondrial function and membrane potential or integrity.

Firstly, the mitochondria functional status was determined by measuring ROS level. Several recent reports suggested that Res and its derivatives can induce apoptosis in cancer cells via ROS-dependent manner. DCFH_2_-DA fluorescence probe was used to detect intracellular ROS levels in the cells by fluorescence microscopy. Our results showed that the relative fluorescence intensity of DCFH_2_-DA was significantly increased in both A549, H292, and H460 cells in a dose-dependent manner (0–50 µM) SM-3 treatment compared to lead compound, Res (Fig. S8B). When high intracellular ROS can attack mitochondria DNA to produce oxidative damage, that results in reduced mitochondria ATP synthesis and mitochondrial membrane potential damage.

The mitochondrial integrity for SM-3 treated NSCLC cells was measured by highly sensitive fluorescence probe JC-1 (5,5′,6,6′-tetrachloro-1,1′,3,3′-tetraethylbenzimidazolocarbocyanine iodide). The SM-3 treated NSCLC cells were low mitochondrial membrane potential and produced green fluorescence, JC-1 cannot aggregate in the matrix of mitochondria as monomer. According to our results, the ratio of aggregate by monomeric ratio decreased in SM-3 treated NSCLC cells than Res treated population (Fig. S9C). In normal mitochondria, JC-1 aggregates in the mitochondria matrix to form as polymer (aggregate) and emits strong red fluorescence.

**Fig. 11:**
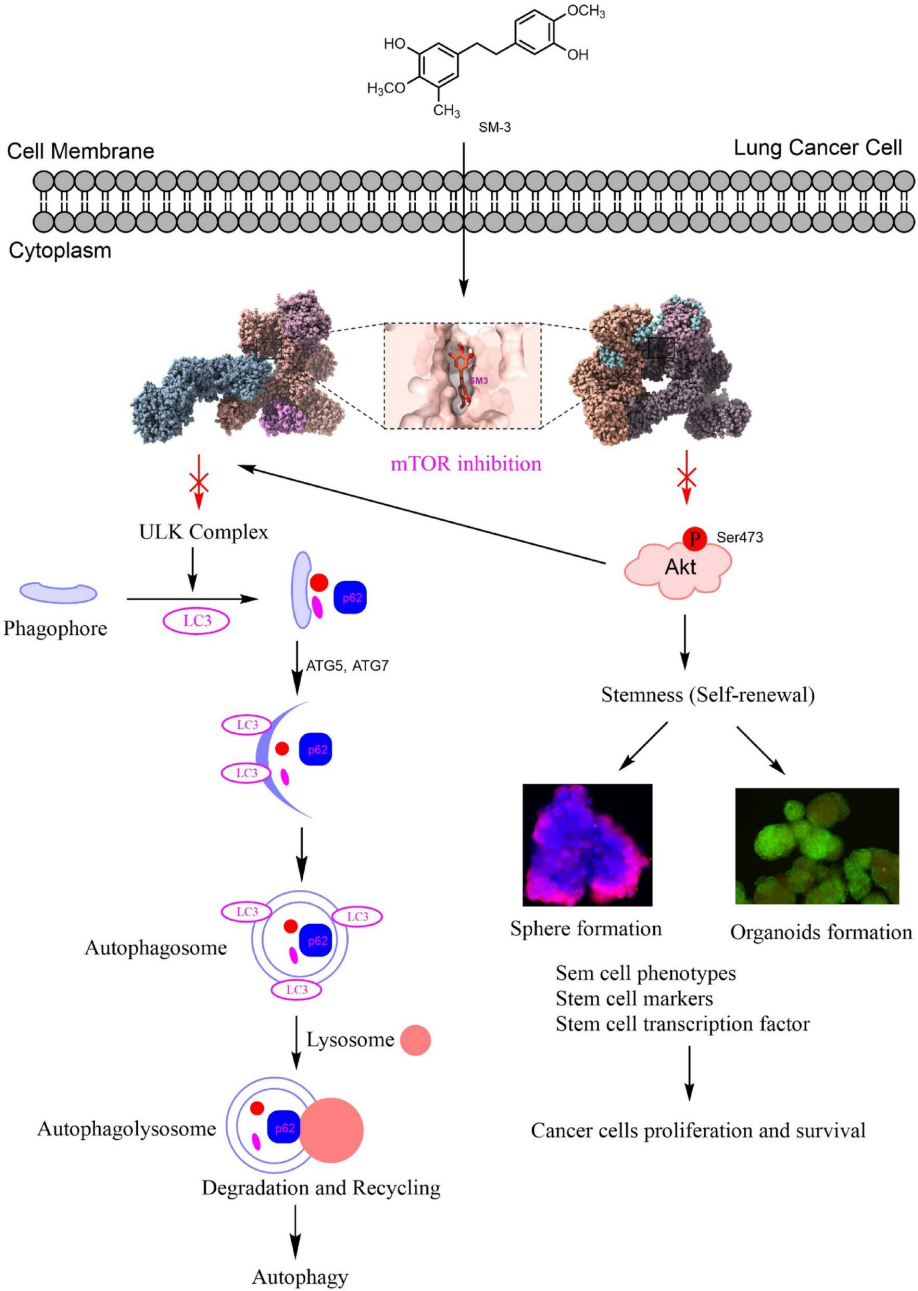
SM-3 treatment induces autophagic cell death in lung cancer cells while reducing CSCs characteristics and stem cell transcription factors by targeting the upstream inhibition of mTOR.

## Discussion

Res is a natural polyphenolic compound found in sources like peanuts, grapes, red and white wine, blueberries, cranberries, cocoa, and dark chocolate. Numerous studies have highlighted its anticancer properties, showing effectiveness against cancers such as oral, breast, and lung cancer^43^. Previous research has also indicated that a derivative of Res, called Moscatilin, may be more effective in inhibiting lung cancer stem cells compared to the parent compound, Res^32^.

Although Res demonstrates anticancer potential, its main limitations are low water solubility, rapid degradation, and poor bioavailability^28,29^. To address these challenges, modifications to the parent structure of Res can be made. To overcome these limitations, we focused on modifying the Res structure by introducing specific functional groups to enhance its effectiveness.

Numerous studies have shown that functional groups like fluoro, methoxy, methyl, amino, hydroxy, nitro, bromo, chloro, methylamino, ethoxy, carbonyl, iodo, and trifluoromethyl exhibit significant antimigration and antiproliferation activities^44^. In our research, we enhanced the anticancer properties of Res by replacing hydroxyl groups with methyl groups on ring A, improving its stability and bioavailability. Modifying the hydroxyl group on ring B with a methoxy group further boosts bioavailability, reduces rapid metabolism, and increases lipophilicity. Based on these modifications, we hypothesize that the Res derivative, SM-3, will demonstrate enhanced anticancer activity and stability compared to the parent compound, Res.

The methyl and methoxy functional groups are crucial for the anticancer activity of the Res structure. Accordingly, we synthesized and assessed the anticancer properties of multiple Res derivatives. In our research, the A ring of the parent compound, Res, was modified by substituting a hydroxyl group with a methyl group, leading to the development of SM-1, SM-2, and SM-3 compounds. In contrast, SM-8 lacked the methyl substitution on the A ring of Res. Cell viability studies showed that SM-8 had the highest IC_50_ values among the Res derivatives, with values of 453.2 ± 0.10, 499.2 ± 0.15, and 266.2 ± 0.11 µM in A549, H292, and H460 cells, respectively (Fig. 2A). These findings suggest that the absence of the methyl functional group diminishes its anticancer efficacy.

For the Res derivative SM-1, the presence of a methyl functional group on both ring A and ring B of Res influences its anticancer activity. However, the position of the methyl group plays a crucial role in determining its efficacy. Methyl substitution at the para position of the ring has demonstrated strong anticancer activity^44^. In contrast, the SM-1 compounds, which feature methyl substitution at the meta position of ring B on Res, exhibit lower potency compared to the parent compound. The SM-1 exhibited IC_50_ values of 379.0 ± 0.08, 493.4 ± 0.13, and 285.8 ± 0.12 µM in A549, H292, and H460 cells, respectively (Fig. 2A). The reduced efficacy with meta substituent methyl group on ring B of Res highlights the importance of the placement and quantity of these functional groups.

The key difference between SM-2 and SM-3 lies in the position of the methoxy substituent on ring B of Res. Methoxy substitution at the para position exhibits greater anticancer activity than at the meta position^45^. This is evident from the significantly different lower IC_50_ values observed in cell viability assays with human lung cancer cells. SM-2, with a meta methoxy substitution on ring B, exhibits IC_50_ values of 223.2 ± 0.13, 148.8 ± 0.41, and 117.3 ± 0.20 µM in A549, H292, and H460 cells, respectively. In contrast, SM-3, with a para methoxy substitution, shows much lower IC_50_ values of 72.74 ± 0.13, 67.66 ± 0.10, and 43.24 ± 0.11 µM in the same cell lines (Fig. 2A). This comparison indicates that SM-3 is more effective in inhibiting lung cancer cells, likely due to the para positioning of the methoxy group on ring B of the parent compound, Res.

To assess the effects of SM-3 on cell proliferation, NSCLCs were treated with varying concentrations of SM-3 (0, 10, 50 µM), and the proliferation rate was measured at 0, 24, 48, and 72 h using an MTT assay. At a concentration of 50 µM, SM-3 significantly reduced the cell proliferation rate compared to the parent compound, Res, in lung cancer cell lines (A549, H292, and H460) after 72 h (Fig. 3A). Additionally, a colony formation assay showed a dose-dependent decrease in the number of colonies in cancer cells treated with SM-3 compared to those treated with Res (Fig. 3B). Both assays demonstrated that SM-3 is more effective at inhibiting cancer cell proliferation than the original Res compound.

Computational methods have become indispensable tools in modern drug discovery, offering insights into protein–ligand interactions and enabling the identification of promising candidates with drug-like properties^46,47^. Techniques such as molecular docking, molecular dynamics (MD) simulations, and free energy calculations provide a detailed mechanistic understanding at the atomic level^48,49^. This study employs these approaches to investigate the molecular interaction between SM-3 and mTOR, aiming to elucidate SM-3’s mechanism of action and assess its potential as a drug-like compound. This computational analysis complements experimental approaches, offering a cost-effective and efficient pathway for hypothesis generation and validation.

The results of this study highlight the potential of SM-3 as a promising mTOR inhibitor, exhibiting superior binding affinity and stability compared to Res. SM-3 demonstrated a lower RMSD throughout the 100 ns MD simulation, maintaining close proximity to the ATP-binding pocket, which suggests a stable binding mode. In contrast, Res underwent significant conformational changes, as reflected by its higher RMSD values and decreased stability during the simulation. The hydrogen bond analysis further supported SM-3’s enhanced stability, showing persistent interactions with Val2240, a key residue in the hinge region critical for mTOR inhibition.

Compared to Res, SM-3 formed stronger van der Waals and electrostatic interactions with mTOR, contributing to its higher binding affinity of −34.02 kcal/mol, as calculated by MM/GBSA. Energy decomposition analysis revealed significant contributions from residues such as Glu2190, Trp2239, Val2240, and Ile2356, underscoring the critical role of these residues in stabilizing SM-3 within the binding site (Fig. 7D). The lower SASA values observed in the SM-3 complex further suggest a more compact and stable interaction compared to the mTOR-Res complex, which displayed increased solvent exposure and potential instability.

These findings align with previous reports that emphasize the importance of robust nonpolar interactions in mTOR inhibition. Moreover, SM-3’s specific interactions with the hinge and affinity pocket residues may confer greater selectivity for mTOR over related kinases such as PI3 Ks. This selectivity is crucial for minimizing off-target effects and improving therapeutic outcomes. The reduced RMSF values in certain mTOR regions upon SM-3 binding also suggest a stabilizing effect on the protein, which could enhance its functional dynamics and inhibitory potential.

The mechanistic target of rapamycin (mTOR) is a serine/threonine kinase that integrates various cellular signals related to cell growth, proliferation, and survival. It consists of two primary complexes: mTORC1 and mTORC2. Activation of Akt stimulates mTORC1 activity by phosphorylating tuberous sclerosis complex 2 (TSC2) and PRAS40, both of which are negative regulators of mTOR^38^. Increased mTORC1 activation leads to the translational upregulation of the anti-apoptotic protein BCL-2. Additionally, mTORC1 serves as the primary regulator of autophagy, which is also activated by Akt.

In contrast to mTORC1, mTORC2 is the protein complex responsible for stabilizing and activating Akt at Thr-450 and Ser-473. Without mTORC2-mediated phosphorylation, Akt becomes inactive and may undergo degradation^50^. As a result, mTOR present promising targets for anticancer therapies aimed at inducing cell death through apoptosis and autophagy^51^.

The results indicate that treatment with SM-3 reduced the activation of mTOR and Akt in lung cancer cells, as evidenced by lower levels of both phosphorylated and total protein expressions compared to the parent compound, Res (Fig. 7).

mTOR is a potential protein target of SM-3, as evidenced by the enhanced cell death observed when the known mTOR inhibitor, rapamycin, was added to SM-3-treated cells. Since mTOR plays a crucial role in cell survival, its inhibition may amplify the cytotoxic effects of SM-3. Furthermore, pretreatment with rapamycin followed by SM-3 resulted in a greater reduction of both mTOR and its phosphorylated form compared to treatment with SM-3 alone (Fig.S2). Mechanistically, rapamycin and SM-3 target mTOR in a different manner, which suggest that they might exhibit synergistic effect. Rapamycin is an allosteric inhibitor that binds to the FKBP12-mTOR interface on the FRB domain, selectively inhibiting mTORC1 but not mTORC2^52,53^. In contrast, we have the data on SM-3 that point toward the direct role as ATP-competitive inhibitor, binding in the mTOR catalytic domain and therefore blocking both mTORC1 and mTORC2 associated functions. Since these two compounds have a distinct mode of action, using them in combination would result in a more comprehensive blockage of mTOR signaling than either agent alone.

The mTOR are essential proteins involved in the regulation of apoptosis and autophagy. Treatment of lung cancer cells with SM-3 resulted in autophagic cell death, as evidenced by the conversion of LC3I to LC3II, along with increased levels of ATG7, ATG5, and p62 (Fig. 10B).

Treatment of lung cancer cells with SM-3 (50 µM) can induce autophagic cell death by reducing several proteins, including Akt and mTOR. Autophagy is an intracellular pathway responsible for the degradation of proteins through lysosomal processes. Autophagy is a highly regulated process involving selective protein degradation. Receptors like p62 and SLRs help target proteins for autophagic degradation, with p62 binding to LC3 at the LIR motif to facilitate their transport to the autophagosome. Additionally, Akt has been shown to accumulate in the autophagosome via its interaction with Phafin2. Similarly, mTOR degradation occurs through a lysosomal pathway^54^.

Since the introduction of the concept of CSCs in the late 1990 s, it has become evident that these long-lived, self-renewing cells may contribute to tumor heterogeneity and treatment resistance. Various treatment strategies have been investigated for NSCLCs, particularly focusing on targeting CSCs and modulating self-renewal pathways as a crucial approach to lung cancer therapy. Substantial evidence indicates that self-renewal capacity and the overexpression of stem cell transcription factors are linked to enhanced cancer cell proliferation, increased invasion and metastasis, drug resistance, and poor clinical outcomes^55^. 3D spheroids, which replicate in vivo conditions and tumor characteristics, offer a more reliable and effective model for in vitro drug screening, aiding in the identification of new anti-cancer agents^56^. Culturing cells in ultra-low attachment plates promotes cell detachment, stimulating and maintaining the self-renewal ability of lung cancer cell populations enriched with CSCs. Additionally, spheroid formation is associated with significantly elevated expression levels of stem cell markers (CD44, CD133, and ALDH1 A1) and stem cell transcription factors (NANOG, OCT4, and SOX2), which support the self-renewal and proliferation of these spheroids^57^.

In our experiments, we observed that treating spheroid formations with SM-3 (50 µM) resulted in a decrease in their size and number. Both primary and secondary spheroids exposed to SM-3 did not exhibit growth compared to those treated with Res (Fig. 9A, S3 A, S4 A). Moreover, lung cancer spheroids treated with SM-3 exhibited higher levels of apoptosis than those treated with Res, as demonstrated by Hoechst 33,342/PI co-staining (Fig. 9B, S3B, S4B). Furthermore, SM-3 treatment led to a significant reduction in the levels of stem cell markers CD133 and CD44, as well as the stem cell transcription factors OCT4 and SOX2, compared to the spheroids treated with Res (50 µM) in lung cancer cells (Fig. 9D, S3D, S4D).

Moreover, lung cancer cells treated with SM-3 exhibited decreased mRNA expression levels of the transcription factors NANOG, OCT4, and SOX2. This reduction inhibited CSCs-rich spheroids, reducing their self-renewal capacity and tumor-forming potential in lung cancer stem cells (Fig. 9G, S3G, S4G). According to western blot and immunofluorescence analyses, SM-3-treated human lung cancer cells (A549, H292, and H460) showed decreased levels of stem cell markers (CD44, CD133, ALDH1 A1) and stem cell transcription factors (NANOG, OCT4, SOX2) (Fig. 9H, S3H, S4H and 9I, S3I, S4I). Overall, SM-3 compounds represent promising therapeutic candidates for targeting human lung cancer cells.

Organoids are created through in vitro 3D cultures and can replicate the structure and physiological functions of organs or tissues found in vivo. Organoids closely mimic physiological structures, offering a more accurate representation of drug responses and bridging the gap between drug screening and clinical trials. Additionally, numerous studies have demonstrated that organoids hold significant potential in new drug development, drug screening, tumor mechanism research, and precision medicine^58,59^.

The results showed that the population of 3D organoids treated with SM-3 exhibited significantly lower levels of pAkt and mTOR protein compared to those treated with Res. These findings suggest that SM-3 exhibits promising anticancer activity, primarily through mTOR inhibition. Furthermore, the 3D organoid population treated with SM-3 showed a marked reduction in the levels of stem cell markers (CD44 and CD133) and stem cell transcription factors (SOX2 and OCT4) in lung cancer organoids (Fig. 9E, S3E, S4E). Overall, the findings from both spheroids and organoids treated with Res derivatives indicate that SM-3 offers a novel strategy for anticancer drug development by targeting cancer stem cells through mTOR inhibition.

Thus, the Res derivative, SM-3 shows great promise as a targeted treatment for CSCs-rich sub-populations in lung cancer. The induction of stem cell transcription factors like OCT4 and NANOG enhances the properties of cancer stem cells and contributes to the malignancy of lung adenocarcinoma. Additionally, the SOX2 transcription factor is involved in tumor development and the maintenance of pluripotency in human lung cancer.

The mTOR target is essential for regulating Akt. To investigate this further, we conducted an experiment showing that LY294002, an Akt inhibitor, can suppress both stemness and organoids formation. Additionally, SM-3 treatment, in combination with LY294002, led to a decreased expression of cancer stem cell markers and transcription factors in spheroids and organoids formation in lung cancer (Fig. S5-S7).

The abnormalities of mitochondrial function and integrity can lead to a variety of intracellular signaling cascades, oxidative stress and the initiation of programmed cell death. Therefore, the detection of mitochondrial abnormalities is crucial for SM-3 treated NSCLC cells. The mitochondrial abnormalities were determined for mitochondrial function and membrane potential or integrity. The mitochondria functional status was determined by measuring ROS level. The DCFH2-DA fluorescence probe revealed a dose-dependent increase in intracellular ROS levels (0–50 µM) in A549, H292, and H460 cells following SM-3 treatment, showing higher fluorescence intensity compared to the lead compound, Res. JC-1 fluorescence analysis confirmed lower mitochondrial membrane potential in SM-3-treated NSCLC cells compared to Res, indicating greater mitochondrial dysfunction and impaired aggregation of JC-1 in mitochondria (Fig. S8).

## Materials and methods

### Synthesis of Resveratrol derivatives

#### 5-(hydroxymethyl)−2-methoxyphenol (**2b**)

A solution of **isovanillin** (5.00 g, 32.9 mmol) in methanol (78 mL), tetrahydrofuran [(THF) 78 mL], and H_2_O (8.0 mL) was added NaBH_4_ (1.37 g, 36.1 mmol, 1.1 equiv.) at 0 °C, and the reaction mixture was stirred for 1.5 h. The reaction was diluted with CH_2_Cl_2_ (30 mL) and quenched with HCl solution (20 mL, 1 mol/L). The obtained solution was evaporated under vacuum. The residue was diluted with H_2_O (80 mL) and extracted with EtOAc (200 mL×3), washed with brine, dried over anhydrous Na_2_SO_4_, and concentrated to give **2b** (4.80 g, 95%) as a colorless solid.

^1^H NMR (300 MHz, CDCl_3_) δ: 6.95 (1 H, m), 6.85 (2 H, d, *J* = 1.8 Hz), 5.63 (1 H, brs), 4.59 (2 H, s), 3.89 (3 H, s).

#### Diethyl (3-hydroxy-4-methoxybenzyl) phosphonate (**3b**)

A solution of **2b** (1.00 g, 6.49 mmol) in Et_2_O (45.3 mL) was added PBr_3_ (800 µL, 8.43 mmol, 1.3 equiv.) at 0 °C. The reaction mixture was stirred at this temperature for 1.5 h. The reaction mixture was concentrated in vacuo and the obtained mixture was diluted with H_2_O (50 mL) and extracted with EtOAc (100 mL×3), washed with saturated NaCl solution and H_2_O, dried over anhydrous Na_2_SO_4_, and concentrated. The crude product was dissolved in triethyl phosphite (1.39 mL, 8.04 mmol, 1.24 equiv.). The reaction mixture was stirred at 140 °C for 4 h. After cooling to room temperature, the reaction mixture was evaporated under vacuum. The residue was purified over SiO_2_ column (n-Hex. : EtOAc = 1 : 9) to give **3b** (1.16 g, 65%) as a yellow oil.

^1^H NMR (400 MHz, CDCl_3_) δ: 6.89–6.90 (1 H, m), 6.75–6.80 (2 H, m), 6.06 (1 H, brs), 3.87–4.09 (4 H, m), 3.87 (3 H, s), 3.06 (2 H, d, *J* = 21.3 Hz), 1.26 (6 H, t, *J* = 7.1 Hz).

#### 2-(benzyloxy)−6-methylphenol (**5**)

A solution 3-methylcatechol (7.00 g, 56.4 mmol) in CH_3_CN (50 mL) was added NaHCO_3_ (7.11 g, 84.7 mmol, 1.5 equiv.), NaI (3.80 g, 25.4 mmol, 0.45 equiv.), and benzyl chloride (13.0 mL, 113 mmol, 2 equiv.). And the reaction mixture was stirred at rt for 89 h. The reaction mixture was filtered, and the obtained filtrate was diluted with H_2_O (500 mL) and extracted with EtOAc (500 mL×3), washed with brine, dried over anhydrous Na_2_SO_4_, and concentrated. The crude product was purified over SiO_2_ column (n-Hex. : EtOAc = 19 : 1) to give **5** (6.84 g, 57%) as a colorless oil.

^1^H NMR (300 MHz, CDCl_3_) δ: 7.25–7.42 (5 H, m), 6.70–6.77 (3 H, m), 5.74 (1 H, s), 5.10 (2 H, s), 2.26 (3 H, s).

#### 3-(benzyloxy)−4-hydroxy-5-methylbenzaldehyde (**6**)

A solution of **5** (6.84 g, 31.9 mmol) in AcOH (50 mL) was added hexamethylenetetramine (HMTA, 13.4 g, 95.8 mmol, 3 equiv.). The reaction mixture was refluxed for 42 h. After cooling to room temperature, the reaction mixture was basified with sat. NaHCO_3_ sol. (500 mL) and extracted with Et_2_O (500 mL×3), washed with brine, dried over anhydrous Na_2_SO_4_, and concentrated. The crude product was purified over SiO_2_ column (n-Hex. : EtOAc = 8 : 2) to give **6** (3.29 g, 43%) as a colorless solid.

^1^H NMR (300 MHz, CDCl_3_) δ: 9.79 (1 H, s), 7.32–7.42 (7 H, m), 6.29 (1 H, s), 5.12 (2 H, s), 2.27 (3 H, s).

#### 3-(benzyloxy)−4-methoxy-5-methylbenzaldehyde (**1 d**)

A solution **6** (277 mg, 1.14 mmol) in acetone (5.7 mL) was added K_2_CO_3_ (474 mg, 34.3 mmol, 3 equiv.) and Me_2_SO_4_ (217 µL, 2.29 mmol, 2 equiv.). And the reaction mixture was stirred at rt for 4 h. The reaction mixture was filtered, and the obtained filtrate was diluted with HCl solution (10 mL, 1 mol/L) and extracted with Et_2_O (20 mL×3), washed with brine, dried over anhydrous Na_2_SO_4_, and concentrated. The crude product was purified over SiO_2_ column (n-Hex. : EtOAc = 8 : 2) to give **1 d** (283 mg, 97%) as a yellow oil.

^1^H NMR (300 MHz, CDCl_3_) δ: 9.84 (1 H, s), 7.31–7.48(7 H, m), 5.16 (2 H, s), 3.93 (3 H, s), 2.33 (3 H, s).

#### [3-(benzyloxy)−4-methoxy-5-methylphenyl] methanol (**2 d**)

A solution of **1 d** (1.67 g, 6.52 mmol) in methanol (15 mL), tetrahydrofuran [(THF) 15 mL], and H_2_O (1.5 mL) was added NaBH_4_ (271 mg, 7.17 mmol, 1.1 equiv.) at 0 °C, and the reaction mixture was stirred for 2 h. The reaction was diluted with Et_2_O (20 mL) and quenched with HCl solution (45 mL, 1 mol/L). The obtained solution was evaporated under vacuum. The residue was diluted with H_2_O (35 mL) and extracted with EtOAc (150 mL×3), washed with brine, dried over anhydrous Na_2_SO_4_, and concentrated. The crude product was purified over SiO_2_ column (n-Hex. : EtOAc = 1 : 1) to give **2 d** (1.61 g, 96%) as a colorless oil.

^1^H NMR (300 MHz, CDCl_3_) δ: 7.29–7.47 (5 H, m), 6.85 (1 H, d, *J* = 1.6 Hz), 6.78 (1 H, m), 5.11 (2 H, s), 4.57 (2 H, s), 3.79 (3 H, s), 2.23 (3 H, s).

#### Diethyl [3-(benzyloxy)−4-methoxy-5-methylbenzyl] phosphonate (**3 d**)

A solution of NBS (482 mg, 2.71 mmol, 3.5 equiv.) in CH_2_Cl_2_ (2.8 mL) was added dimethylsulfide (238 µL, 3.26 mmol, 4.2 equiv.) at 0 °C over 6 min. The reaction mixture was stirred at this temperature for 10 min. A solution of **2a** (200 mg, 0.774 mmol) in CH_2_Cl_2_ (2.8 mL) was cooled at − 18 °C and was added above solution. The reaction mixture was stirred at − 18 °C for 2 h. The reaction mixture was warmed to 0 °C and diluted with H_2_O and extracted with CH_2_Cl_2_ (10 mL×3), washed with saturated NaHCO_3_ solution and H_2_O, dried over anhydrous Na_2_SO_4_, and concentrated. The crude product was dissolved in triethyl phosphite (167 µL, 0.960 mmol, 1.24 equiv.). The reaction mixture was stirred at 140 °C for 4 h. After cooling to room temperature, the reaction mixture was evaporated under vacuum. The residue was purified over SiO_2_ column (n-Hex. : EtOAc = 1 : 1) to give **3 d** (203 mg, 69%) as a colorless oil.

^1^H NMR (400 MHz, CDCl_3_) δ: 7.45 (2 H, d, *J* = 7.3 Hz), 7.38 (2 H, t, *J* = 7.3 Hz), 7.31 (1 H, t, *J* = 7.3 Hz), 6.79 (1 H, s), 6.70 (1 H, s), 5.10 (2 H, s), 3.92–4.06 (4 H, m), 3.82 (3 H, s), 3.03 (2 H, d, *J* = 21.5 Hz), 2.25 (3 H, s) 1.24 (6 H, t, *J* = 7.3 Hz).

^13^C NMR (100 MHz, CDCl_3_) δ: 151.6 (C), 146.8 (C), 137,1 (C), 132.0 (C), 128.5 (CH), 127.8 (CH), 127.2 (CH), 126.7 (C), 124.6 (CH), 113.4 (CH), 70.5 (CH_2_), 62.1 (CH_2_), 60.2 (CH_3_), 33.4 (CH_2_), 16.4 (CH_3_), 15.8 (CH_3_). IR (KBr cm^−1^): 2994,1591, 1495, 1331, 1215, 1148, 1029, 966, 741, 668, 490. EI-MS *m/z* (%): 379 (21), 378 (100), 287 (30), 241 (10), 151 (12), 91 (51). HRMS (EI): Calcd for C_20_H_27_O_5_P, 378.1596; Found: *m/z* 378.1598.

#### (*E*)−1,2-bis[3-(benzyloxy)−4-methoxy-5-methylphenyl] Ethene (**7a**)

A solution of **3 d** (257 mg, 0.679 mmol, 1.2 equiv.) in THF (3.4 mL) was stirred at − 78 °C and added *t*-BuOK solution in THF (913 µL, 1.6 equiv., 1.0 M) over 30 min. The reaction mixture was stirred for 20 min. at the same temperature, and was added **1 d** (145 mg, 0.566 mmol) in THF (620 µL) over 20 min. and the mixture was stirred for 1 h at − 78 °C and for 10 min. at 0 °C. Then, the reaction mixture was stirred for 22 h at room temperature. The reaction mixture was cooled to 0 °C and diluted with saturated NH_4_Cl solution and extracted with EtOAc (40 mL×3), washed with saturated NH_4_Cl solution and H_2_O, dried over anhydrous Na_2_SO_4_, and concentrated. The crude product was purified over SiO_2_ column (n-Hex. : EtOAc = 1 : 9) to give **7a** (265 mg, 97%) as a colorless solid.

^1^H NMR (400 MHz, CDCl_3_) δ: 7.48 (4 H, d, *J* = 6.8 Hz), 7.40 (4 H, t, *J* = 6.8 Hz), 7.33 (2 H, t, *J* = 6.8 Hz), 6.94 (4 H, d, *J* = 3.9 Hz), 6.84 (2 H, s), 5.15 (4 H, s), 3.85 (6 H, s), 2.29 (6 H, s).

^13^C NMR (100 MHz, CDCl_3_) δ: 151.9 (C), 147.6 (C), 137.2 (C), 133.0 (C), 132.1 (C), 128.6 (CH), 127.9 (CH), 127.5 (CH), 127.3 (CH), 121.7 (CH), 109.8 (CH), 70.8 (CH_2_), 60.3 (CH_3_), 16.0 (CH_3_). IR (KBr cm^−1^): 3683, 3019, 2400, 1499, 1216, 775, 670, 489, 482, 467, 461, 439. EI-MS *m/z* (%): 481 (33), 480 (100), 91 (35). HRMS (EI): Calcd for C_32_H_32_O_4_, 480.2301; Found: *m/z* 480.2298.

#### 5,5’-(ethane-1,2-diyl) bis(2-methoxy-3-methylphenol) (**8a**, **SM-1**)

A solution of **7a** (209 mg, 0.435 mmol) in THF (23.6 mL) was hydrogenated over 10% Pd/C (55% water, 93.0 mg) at room temperature for 18 h. The catalyst was removed by celite filtration and the filtrate was concentrated in vacuo to give **8a** (**SM_1**, 130 mg, 99%) as a colorless solid.

^1^H NMR (400 MHz, CDCl_3_) δ: 6.67 (2 H, d, *J* = 1.5 Hz), 6.54 (2 H, s), 5.57 (2 H, brs), 3.78 (6 H, s), 2.74 (4 H, s), 2.27 (6 H, s).

^13^C NMR (100 MHz, CDCl_3_) δ: 148.5 (C), 143.5 (C), 138.4 (C), 130.4 (C), 122.4 (CH), 113.0 (CH), 60.7 (CH_3_), 37.5 (CH_2_), 15.8 (CH_3_). IR (KBr cm^−1^): 3277, 3019, 1215, 755, 669, 482, 458, 454, 426, 419, 406. EI-MS *m/z* (%): 302 (33), 151 (100). HRMS (EI): Calcd for C_18_H_22_O_4_, 302.1518; Found: *m/z* 302.1517.

#### (*E*)−1-(benzyloxy)−5-(4-(benzyloxy)−3-methoxystyryl)−2-methoxy-3-methylbenzene (**7b**)

A solution of **3a** (400 mg, 1.10 mmol, 1.2 equiv.) in THF (5.5 mL) was stirred at − 78 °C and added *t*-BuOK solution in THF (1.46 mL, 1.46 mmol, 1.6 equiv., 1.0 M) over 30 min. The reaction mixture was stirred for 20 min. at the same temperature, and was added **1 d** (235 mg, 0.915 mmol) in THF (1.0 mL) over 20 min. and the mixture was stirred for 1 h at − 78 °C and for 10 min. at 0 °C. Then, the reaction mixture was stirred for 2.5 h at room temperature. The reaction mixture was cooled to 0 °C and diluted with saturated NH_4_Cl solution and extracted with EtOAc (60 mL×3), washed with saturated NH_4_Cl solution and H_2_O, dried over anhydrous Na_2_SO_4_, and concentrated. The crude product was purified over SiO_2_ column (CH_2_Cl_2_) to give **7b** (354 mg, 83%) as a colorless solid.

^1^H NMR (400 MHz, CDCl_3_) δ: 7.28–7.65 (10 H, m), 7.05 (1 H, d, *J* = 1.5 Hz), 6.94–6.96 (3 H, m), 6.89 (1 H, d, *J* = 16.1 Hz), 6.85 (1 H, d, *J* = 8.8 Hz), 6.84 (1 H, d, *J* = 16.1 Hz), 5.17 (2 H, s), 5.15 (2 H, s), 3.94 (3 H, s), 3.85 (3 H, s), 2.29 (3 H, s).

^13^C NMR (100 MHz, CDCl_3_) δ: 151.9 (C), 149.8 (C), 147.9 (C), 147.5 (C), 137.2 (C), 137.0 (C), 133.1 (C), 132.1 (C), 131.0 (C), 128.5 (CH), 127.8 (CH), 127.8 (CH), 127.5 (CH), 127.3 (CH), 127.2 (CH), 126.7 (CH), 121.6 (CH), 119.5 (CH), 114.0 (CH), 109.8 (CH), 109.3 (CH), 71.0 (CH_2_), 70.8 (CH_2_), 60.3 (CH_3_), 56.0 (CH_3_), 16,0 (CH_3_). IR (KBr cm^−1^): 3019, 2400, 1583, 1510, 1454, 1422, 1331, 1246, 1215, 1139, 1091, 1008, 958, 756, 697, 668, 489, 419. EI-MS *m/z* (%): 467 (12), 466 (37), 376 (25), 375 (100), 91 (47). HRMS (EI): Calcd for C_31_H_30_O_4_, 466.2144; Found: *m/z* 466.2142.

#### 5-(4-hydroxy-3-methoxyphenethyl)−2-methoxy-3-methylphenol (**8b**, **SM-2**)

A solution of **7b** (162 mg, 0.347 mmol) in THF (6.0 mL) was hydrogenated over 10% Pd/C (55% water, 74.0 mg) at room temperature for 2.5 h. The catalyst was removed by celite filtration and the filtrate was concentrated in vacuo to give **8b** (**SM_2**, 99.4 mg, 99%) as a colorless solid.

^1^H NMR (400 MHz, CDCl_3_) δ: 6.84 (1 H, d, *J* = 8.0 Hz), 6.69 (1 H, dd, *J* = 1.9, 8.0 Hz), 6.66 (1 H, d, *J* = 2.0 Hz), 6.65 (1 H, d, *J* = 1.9 Hz), 6.52 (1 H, d, *J* = 2.0 Hz), 5.60 (1 H, s), 5.50 (1 H, s), 3.85 (3 H, s), 3.77 (3 H, s), 2.73–2.83 (4 H, m), 2.27 (3 H, s).

^13^C NMR (100 MHz, CDCl_3_) δ: 148.5 (C), 146.2 (C), 143.7 (C), 143.5 (C), 138.4 (C), 133.8 (C), 130.4 (C), 122.5 (CH), 120.9 (CH), 114.2 (CH), 113.0 (CH), 111.0 (CH), 60.7 (CH_3_), 55.8 (CH_3_), 37.8 (CH_2_), 37.5 (CH_2_), 15.8 (CH_3_). IR (KBr cm^−1^): 3421, 2945, 1593, 1514, 1429, 1270, 1231, 1034, 527, 495, 483, 470, 459, 453, 439, 427, 421, 414, 402. EI-MS *m/z* (%): 289 (10), 288 (59), 152 (13), 151 (100), 137 (86), 22 (12), 332 (28), 331 (100). HRMS (EI): Calcd for C_17_H_20_O_4_, 288.1362; Found: *m/z* 288.1360.

#### (*E*)−5-[3-(benzyloxy)−4-methoxy-5-methylstyryl]−2-methoxyphenol (**7c**)

A solution of **3b** (200 mg, 0.729 mmol, 1.2 equiv.) in THF (3.6 mL) was stirred at − 78 °C and added *t*-BuOK solution in THF (2.22 mL, 2.19 mmol, 3.6 equiv., 1.0 M) over 30 min. The reaction mixture was stirred for 20 min. at the same temperature, and was added **1 d** (156 mg, 0.608 mmol) in THF (740 µL) over 16 min. and the mixture was stirred for 1 h at − 78 °C and for 10 min. at 0 °C. Then, the reaction mixture was stirred for 2 h at room temperature. The reaction mixture was cooled to 0 °C and diluted with saturated NH_4_Cl solution and extracted with EtOAc (35 mL×3), washed with saturated NH_4_Cl solution and H_2_O, dried over anhydrous Na_2_SO_4_, and concentrated. The crude product was purified over SiO_2_ column (n-Hex. : EtOAc = 1 : 1) to give **7c** (83 mg, 36%) as a yellow solid.

^1^H NMR (400 MHz, CDCl_3_) δ: 7.48 (2 H, d, *J* = 7.4 Hz), 7.40 (2 H, t, *J* = 7.4 Hz), 7.33 (1 H, t, *J* = 7.4 Hz), 7.11 (1 H, d, *J* = 2.1 Hz), 6.93–6.96 (3 H, m), 6.87 (1 H, d, *J* = 16.7 Hz), 6.83 (1 H, d, *J* = 16.7 Hz), 6.82 (1 H, d, *J* = 8.2 Hz), 5.62 (1 H, brs), 5.15 (2 H, s), 3.89 (3 H, s), 3.85 (3 H, s), 2.28 (3 H, s).

^13^C NMR (100 MHz, CDCl_3_) δ: 151.8 (C), 147,4 (C), 146.2 (C), 145.7 (C), 137.2 (C), 133.1 (C), 132.1 (C), 131.2 (C), 128.5 (CH), 127.8 (CH), 127.4 (CH), 127.3 (CH), 126.8 (CH), 121.6 (CH), 119.1 (CH), 111.6 (CH), 110.6 (CH), 109.7 (CH), 70.7 (CH_2_), 60.3 (CH_3_), 56.0 (CH_3_), 16.0 (CH_3_). IR (KBr cm^−1^): 3684, 3019, 2400, 1510, 1215, 750, 668, 485, 454, 443, 424, 415. EI-MS *m/z* (%): 377 (24), 376 (100), 257 (26), 225 (16), 91 (25). HRMS (EI): Calcd for C_24_H_24_O_4_, 376.1675; Found: *m/z* 376.1671.

#### 5-(3-hydroxy-4-methoxyphenethyl)−2-methoxy-3-methylphenol (**8c**)

A solution of **7c** (56.0 mg, 0.149 mmol) in THF (8.1 mL) was hydrogenated over 10% Pd/C (55% water, 32.0 mg) at room temperature for 20 h. The catalyst was removed by celite filtration and the filtrate was concentrated in vacuo to give **8c** (**SM_3**, 42.9 mg, quant.) as a colorless solid.

^1^H NMR (400 MHz, CDCl_3_) δ: 6.79 (1 H, d, *J* = 2.4 Hz), 6.77 (1 H, d, *J* = 8.3 Hz), 6.65–6.68 (2 H, m), 6.53 (1 H, d, *J* = 1.5 Hz), 5.59 (2 H, brs), 3.86 (3 H, s), 3.77 (3 H, s), 2.72–2.81 (4 H, m), 2.27 (3 H, s).

^13^C NMR (100 MHz, CDCl_3_) δ: 148.5 (C), 145.4 (C), 144.8 (C), 143.5 (C), 138.5 (C), 135.3 (C), 130.4 (C), 122.4 (CH), 119.7 (CH), 114.6 (CH), 113.0 (CH), 110.6 (CH), 60.7 (CH_3_), 56.0 (CH_3_), 37.6 (CH_2_), 37.2 (CH_2_), 15.8 (CH_3_). IR (KBr cm^−1^): 3539, 3019, 1213, 741, 668, 505, 473, 456, 448, 441, 426, 418, 409, 403. EI-MS *m/z* (%): 288 (55), 151 (100), 137 (66). HRMS (EI): Calcd for C_17_H_20_O_4_, 288.1362; Found: *m/z* 288.1361.

#### (*E*)−1,2-bis(3-(benzyloxy)−4-methoxyphenyl) Ethene (**7 d**)

A solution of **3c** (200 mg, 0.549 mmol, 1.2 equiv.) in THF (2.8 mL) was stirred at − 78 °C and added *t*-BuOK solution in THF (732 µL, 0.732 mmol, 1.6 equiv., 1.0 M) over 30 min. The reaction mixture was stirred for 20 min. at the same temperature, and was added **1 d** (110 mg, 0.457 mmol) in THF (500 µL) over 20 min. and the mixture was stirred for 1 h at − 78 °C and for 10 min. at 0 °C. Then, the reaction mixture was stirred for 2 h at room temperature. The reaction mixture was cooled to 0 °C and diluted with saturated NH_4_Cl solution and extracted with EtOAc (60 mL×3), washed with saturated NH_4_Cl solution and H_2_O, dried over anhydrous Na_2_SO_4_, and concentrated. The crude product was purified over SiO_2_ column (CH_2_Cl_2_) to give **7 d** (101 mg, 49%) as a colorless solid.

^1^H NMR (400 MHz, CDCl_3_) δ: 7.47 (4 H, d, *J* = 7.3 Hz), 7.38 (4 H, t, *J* = 7.3 Hz), 7.31 (2 H, t, *J* = 7.3 Hz), 7.06 (2 H, d, *J* = 1.5 Hz), 7.02 (2 H, d, *J* = 1.5, 8.3 Hz), 6.87 (2 H, d, *J* = 8.3 Hz), 6.78 (2 H, s), 5.19 (4 H, s), 3.89 (6 H, s).

^13^C NMR (100 MHz, CDCl_3_) δ: 149.4 (C), 148.3 (C), 137.1 (C), 130.6 (C), 128.6 (CH), 127.9 (CH*)*, 127.4 (CH*)*, 126.6 (CH), 120.0 (CH), 111.9 (CH), 111.7 (CH), 71.2 (CH_2_), 56.1 (CH_3_). IR (KBr cm^−1^): 3687, 3019, 2399, 1215, 756, 669, 458, 445, 431, 417, 407. EI-MS *m/z* (%): 453 (31), 452 (100), 270 (14), 91 (79). HRMS (EI): Calcd for C_30_H_28_O_4_, 452.1988; Found: *m/z* 452.1988.

#### 5,5’-(ethane-1,2-diyl) bis(2-methoxyphenol) (**8 d**)

A solution of **7 d** (70.0 mg, 0.155 mmol) in THF (8.4 mL) was hydrogenated over 10% Pd/C (55% water, 33.2 mg) at room temperature for 20 h. The catalyst was removed by celite filtration and the filtrate was concentrated in vacuo to give **8 d** (**SM_8**, 42.4 mg, quant.) as a colorless solid.

^1^H NMR (400 MHz, CDCl_3_) δ: 6.78 (2 H, d, *J* = 1.8 Hz), 6.76 (2 H, d, *J* = 8.3 Hz), 6.65 (2 H, dd, *J* = 1.8, 8.3 Hz), 5.55 (2 H, brs), 3.86 (6 H, s), 2.79 (4 H, s).

^13^C NMR (100 MHz, CDCl_3_) δ: 145.4 (C), 144.7 (C), 135.2 (C), 119.7 (CH), 114.6 (CH), 110.5 (CH), 56.0 (CH_3_), 37.4 (CH_2_). IR (KBr cm^−1^): 3383, 3019, 1514, 1215, 754, 668, 471, 457, 446, 418, 404. EI-MS *m/z* (%): 274 (35), 137 (100). HRMS (EI): Calcd for C_16_H_18_O_4_, 274.1205; Found: *m/z* 274.1207.

### Cell culture

The human non-small cell lung cancer (NSCLC) cell lines A549, H292, and H460 were obtained from the American Type Culture Collection (ATCC, Manassas, VA, USA). The accession numbers for A549, H292, and H460 from ATCC (Homo sapiens) are CVCL_0023, CVCL_0455, and CVCL_0459, respectively. A549 cells were cultured in Dulbecco’s Modified Eagle’s Medium (DMEM) with high glucose (cat no: 12800-058, Gibco, Grand Island, NY, USA). The H292 and H460 cells were maintained in Roswell Park Memorial Institute (RPMI) 1640 medium (cat no: 11875-093, Gibco, Grand Island, NY, USA). Both media were supplemented with 10% fetal bovine serum (FBS) (cat no: SV30160.03, HyClone™, Cytiva, Global life Sciences, Austria), 2 mM L-glutamine (Ref no: 35050-061, Gibco, Gaithersburg, MA, USA), and 100 units/mL of antibiotic-antimycotic (Ref no: 15240-062, Gibco, Grand Island, NY, USA) and the cells were incubated at 37 °C with 5% CO2 until they reached 70–80% confluence for subsequent experiments.

### Reagents and antibodies

Dulbecco’s Modified Eagle’s Medium (DMEM), Roswell Park Memorial Institute (RPMI) 1640 medium, L-glutamine, fetal bovine serum (FBS), 100 units/mL antibiotic-antimycotic, 0.25% trypsin-EDTA (Cat no: SH30042.02, HyClone™, Cytiva, Global life Sciences, Austria), and phosphate-buffered saline (PBS) used for cell culture were obtained from Gibco (Grand Island, NY, USA). The reagents 3-(4,5-Dimethylthiazol-2-yl)−2,5-diphenyl tetrazolium bromide (MTT) (Cat no: M5655), bovine serum albumin (BSA) (Cat no: 9048-46-8),Hoechst 33,342 (Cat no: B2261), propidium iodide (PI) (Cat no: P4170), monodansylcadaverine (Cat no: 30432), Rapamycin (Cat no: 553210), Akt inhibitor, LY 294,002 (Cat no: 440202), Resazurin (Cat no: R7017), 2′,7′-Dichlorofluorescin diacetate, DCFH-DA (Cat no: D6883), and crystal violet (Cat no: C0775) were purchased from Sigma-Aldrich, Co. (St. Louis, MO, USA). Primary antibodies, including rabbit monoclonal antibodies for Akt (cat no: 9272), p-Akt (Ser473, cat no: 4060), mTOR (cat no: 2983), p-mTOR (cat no: 5536), ALDH1 A1 (cat no: 36671), β-actin (cat no: 4970) as well as mouse monoclonal antibodies for OCT4 (cat no: 9B7), CD44 (cat no: 3570) were sourced from Cell Signaling (Beverly, MA, USA). The mouse monoclonal antibodies for CD133 (cat no: MAB4399-I) from Millipore sigma. Additionally, primary rabbit monoclonal antibodies against OCT4 (cat no: ab19857), NANOG (cat no: ab80892), SOX2 (cat no: ab97959), and CD133 (cat no: ab19898) were obtained from Abcam (Waltham, MA, USA). The rabbit secondary antibodies, anti-rabbit (cat no: 7074) and anti-mouse IgG (cat no: 7076), were also provided by Cell Signaling. Alexa Fluor™ 594 goat anti-rabbit IgG (H + L) (Ref no: A11037) and Alexa Fluor™ 488 goat anti-mouse IgG (H + L) conjugated secondary antibodies (Ref no: A11029) were acquired from Invitrogen by Thermo Fisher Scientific (Carlsbad, CA, USA). All primers for OCT4, NANOG, SOX2, and GAPDH were sourced from Eurofins Genomics (North America, US). JC-1 staining (Cat no: HY-15534) was purchased from MedChemExpress.

### Preparation of stock solution for res derivatives

The Res derivatives (SM-1, SM-2, SM-3, and SM-8) were made into a 50 mM stock solution by dissolving them in dimethyl sulfoxide (DMSO) and were then stored at −20 °C. Under high treatment conditions, the final concentration of DMSO was 0.2% v/v, which did not exhibit any cytotoxic effects.

### Cell viability assay

Cell viability was evaluated using the MTT assay to determine the half-maximal inhibitory concentration (IC_50_) of the Res derivatives (SM-1, SM-2, SM-3, and SM-8). Human NSCLC cells (A549, H292, and H460) were seeded at a density of 1 × 10^4^ cells per well in 96-well plates and incubated for 24 h at 37 °C. The cells were then treated with various concentrations of Res derivatives (0–200 µM) for another 24 h. The following day, the medium containing the Res derivatives was removed and replaced with 4 mg/mL MTT reagent in PBS. After incubating for 3–4 h, 100 µL of dimethyl sulfoxide was added to dissolve the formazan crystals, and the absorbance of the formazan product was measured at 570 nm using a microplate reader. Res was used as a positive control in this experiment.

### Nuclear staining assay

Apoptosis and necrotic cell death were evaluated using co-staining with Hoechst 33,342 and propidium iodide (PI). Human NSCLC cells were seeded at a density of 1 × 10^4^ cells per well in a 96-well plate and incubated at 37 °C. The cells were then treated with varying concentrations of the Res derivative SM-3 (0, 10, 50, and 100 µM) for 24 h. After treatment, the cells were washed with PBS and co-stained with Hoechst 33,342 (10 µg/mL) and propidium iodide (PI) (0.02 µg/mL) for 30 min at 37 °C. Fragmented nuclei, indicative of apoptosis, were stained with Hoechst 33,342, while necrotic cells were identified by positive PI staining. The stained cells were visualized and captured using fluorescence microscopy (Olympus IX51 with a DP70 digital camera, Olympus, Tokyo, Japan). In this experiment, Res was used as the positive control.

### Lipinski and PAINS filters

To ensure that the observed activity of SM-3 is not due to false positives or poor drug-likeness, we used the SWISS ADME^60^server for a comprehensive evaluation of the identified compounds. This analysis included assessing their compliance with Lipinski’s Rule of Five^61^to determine drug-likeness and identifying potential Pan-Assay Interference Compounds (PAINS)^62^ liabilities to rule out structural features associated with assay interference. These evaluations ensured the compounds possess favorable characteristics for drug discovery and are free from unreliable effects.

### Molecular docking

To assess the mode of action for inhibiting the kinase activity of mTOR, we carried out docking simulations of Res and SM-3 in ATP binding site of the mTOR. The crystal structure of mTOR in complex with the inhibitor, PI-103 (PDB code: 4 JT6)^63^as the receptor for docking simulations with SM-3 and Res, was downloaded from the RCSB Protein Data Bank (RCSB.org)^64^. The receptor was processed using UCSF Chimera 1.16^65^by removing all non-protein components, and the structure was saved in Mol2 format. The 3D conformers of ligands were constructed using SEQCROW^66^, and then optimized with the B3LYP/6-31G* basis set in the Gaussian 09 program^67^. Both the receptor and ligand were prepared by adding hydrogen atoms and assigning AM1BCC^68,69^charges. Molecular docking was performed using the DOCK 6.10 package^70^with a default protocol. The binding energy was calculated using a Hawkins GB/SA scores approach with the computationally intensive flexible docking algorithm. Finally, LigPlot + program^71^was used to generate 2D ligand-protein interaction diagrams, and the UCSF ChimeraX program^72^ was used to visualize 3D molecular structures.

### Molecular dynamics (MD) simulations and free energy calculations

The predicted binding modes of targeted compounds from molecular docking studies were used as initial structures for the molecular dynamics (MD) simulation using the Amber18^73^. The protein and compound parameters were used for the FF14SB force field^74^and generalized AMBER force field version 2 (GAFF2)^75^plus AM1-BCC. Next, we minimized and equilibrated the system following the same protocol as in previous reports^76,77^. After confirming that the system had reached equilibrium, we conducted a 100 ns production MD simulation. In this phase, only the protein was weakly restrained, while the ligand remained unrestrained, as in the final two steps of equilibration. The stability between the ligands and mTOR were measured by the root mean square deviation (RMSD), root mean square fluctuation (RMSF), solvent-accessible surface area (SASA), and hydrogen bond analysis using the CPPTRAJ^78^. The Molecular Mechanics Generalized Born Surface Area (MM/GBSA) method using the MMPBSA.py module^79^ in AMBER18 calculated the binding free energy for the complex systems.

### Proliferation assay

The MTT assay was used to assess the antiproliferative effects of the Res derivative SM-3 over three consecutive days. Human NSCLC cell lines A549, H292, and H460 were seeded at a density of 2 × 10^3^ cells per well in a 96-well plate. The cells were treated with varying concentrations of SM-3 (0, 10, and 50 µM) at different time points (0 h, 24 h, 48 h, and 72 h) and incubated in a 5% CO_2_ atmosphere at 37 °C. Cell proliferation was measured over three days using 4 mg/mL of 3-(4,5-dimethylthiazol-2-yl)−2,5-diphenyltetrazolium bromide (Sigma–Aldrich, St. Louis, MO, USA), with absorbance read at 570 nm. The percentage viability was calculated using the formula: Percentage viability = (Average A570 of treated cells/Average A570 of control cells) × 100. Res was used as the positive control in this experiment.

### Colony formation assay

The clonogenic or colony formation assay was employed to investigate the ability of cancer cells to proliferate from individual cells and develop tumor colonies following SM-3 treatment. A suspension of NSCLC cells (A549, H292, and H460) was seeded at a density of 250 cells per well in a 6-well plate and allowed to adhere for 12 h. The cells were then treated with various concentrations of SM-3 (0, 10, and 50 µM) for 24 h. After treatment, the drug-containing medium was removed and replaced with fresh complete medium. The colonies were incubated for 7 days, with medium changes every 2 days. Following this period, the colonies were washed with PBS and fixed using a methanol and acetic acid solution (3:1 v/v) for 5 min. They were then stained with crystal violet (0.05% w/v in 4% paraformaldehyde) for 30 min. Excess crystal violet was rinsed off with distilled water until a clear background was achieved, and the samples were allowed to air-dry at room temperature. The colonies were photographed, and the images were analyzed using ImageJ software to count the stained colonies. Res served as the positive control in this experiment.

### Western blot analysis

Human lung cancer cells were plated at a density of 3 × 10^5^ cells per well in 6-well culture plates. These cells were treated with SM-3 at concentrations of 0, 10, and 50 µM for 24 h at 37˚C. Following treatment, the cells were harvested and rinsed with ice-cold PBS. The cells were then incubated in a lysis buffer composed of 50 mM 4-(2-hydroxyethyl)−1-piperazineethanesulfonic acid (pH 7.5), 150 mM NaCl, 5 mM EDTA, 1% Triton X-100, 1 mM phenylmethylsulfonylfluoride, and 2 µg/mL pepstatin A, supplemented with a complete protease inhibitor cocktail from EASYpack (Roche, cat no: 04693116001), for 40 min on ice. The mixture was centrifuged at 12,000× g for 15 min at 4 °C to collect the lysate, and the protein content was quantified using the Bicinchoninic acid (BCA) protein kit (Thermo-Fisher Scientific, Rockford, IL, USA). An equal volume of the sample was denatured by heating at 95˚C for 5 min with a sample buffer. The protein was loaded onto a sodium dodecyl sulfate polyacrylamide gel electrophoresis (SDS-PAGE) gel. After separating the proteins, they were transferred onto a 0.45 μm nitrocellulose membrane. The membrane was blocked with 5% non-fat milk in TBST (25 mM Tris-HCl, pH 7.5, 125 mM NaCl, 0.05% Tween 20) for 1 h and then incubated with primary antibodies overnight at 4 °C. β-Actin was used as a loading control, with all primary antibodies diluted to 1:1000 in 5% w/v BSA in TBST. The following day, the membrane was washed three times with TBST for 5 min each time and then incubated with horseradish peroxidase (HRP)-conjugated isotype-specific secondary antibodies (anti-rabbit and anti-mouse IgG, diluted 1:2000 in 5% w/v skim milk in TBST) for 1 h at room temperature. The membranes were washed again with TBST. Chemiluminescent substrate (Supersignal West Pico, Pierce, Rockford, IL, USA) was applied to visualize protein reactivity, and the signal intensity was quantified using densitometry or iBright™ CL 1500 Imaging System (Cat no: A44240, Invitrogen™). Protein intensity was analyzed using ImageJ software (Image J 1.52a, Rasband, W., National Institutes of Health, USA). Res served as the positive control in this experiment.

### Quantitative analysis for real- time PCR analysis

RNA extraction was conducted on cells treated with SM-3 (3 × 10^5^ cells per well in 6-well plates) using GENEzol reagent. The total RNA extracted was utilized to synthesize complementary DNA (cDNA) using SuperScript III reverse transcriptase from Invitrogen. Reverse transcription quantitative polymerase chain reaction (RT-qPCR) was performed with 100 ng of cDNA, utilizing Luna^®^ Universal qPCR Master Mix (NEB, UK) for a final reaction volume of 20 µL. The reactions were carried out using the CFX 96 Real-time PCR system from Bio-Rad in Hercules, CA. The RT-PCR protocol consisted of an initial denaturation step at 95˚C for 1 min, followed by 45 cycles of denaturation at 95˚C for 15 s and primer annealing at 60˚C for 30 s. Melting curve analysis was performed to verify the specificity of the primers. The targeted gene of primers are:

OCT4 (Fwd) TCGAGAACCGAGTGAGAGG, Tm = 58.8˚C.

OCT4 (Rev) GAACCACACTCGGACCACA, Tm = 58.8˚C.

NANOG (Fwd) ATGCCTCACACGGAGACTGT, Tm = 59.4˚C.

NANOG (Rev) AAGTGGGTTGTTTGCCTTTG, Tm = 55.3˚C.

SOX2 (Fwd) TGATGGAGACGGAGCTGAA, Tm = 56.7˚C.

SOX2 (Rev) GGGCTGTTTTTCTGGTTGC Tm = 56.7˚C.

GAPDH (Fwd) GCTCAGAACACCTATGGGGAA Tm = 59.8˚C.

GAPDH (Rev) CATCGCCCCACTTGATTTGG Tm = 59.8˚C.

The PCR products were normalized using GAPDH genes as an internal control. The relative mRNA expression levels of the target gene were determined from the comparative Cq values. Res served as the positive control for this experiment.

### Immunofluorescence

Human lung cancer cells were seeded at a concentration of 1 × 10^4^ cells per well in 96-well plates and treated with various concentrations of SM-3 (0, 10, and 50 µM) for 24 h. Following treatment, the cells were washed with PBS, fixed with 4% paraformaldehyde for 10 min, and permeabilized using 0.5% Triton-X in PBS for 5 min at room temperature. Non-specific proteins were blocked with 10% FBS in 0.1% Triton-X PBS for 1 h at room temperature. The cells were then exposed to a 1:400 dilution of primary antibodies and incubated overnight at 4˚C. The next day, the cells were washed with 10% FBS in 0.1% Triton-X and subsequently incubated with secondary antibodies: Alexa Fluor 488 conjugated goat anti-rabbit IgG (H + L) and Alexa Fluor 488 conjugated goat anti-mouse IgG (H + L), along with Hoechst 33,342 for nuclear staining for 1 h at room temperature. After washing with PBS, the cells were fixed with 50% glycerol. Immunofluorescence images were captured using a fluorescence microscope (Olympus - IX51 with a DP70 digital camera, Olympus, Tokyo, Japan), and fluorescence intensity was analyzed using ImageJ software (ImageJ 1.52a, Rasband, W., National Institutes of Health, USA). Res served as the positive control for this experiment.

### Monodansylcadaverine staining

Human cancer cells were treated with different concentrations of SM-3 (0–100 µM) for 24 h. Afterward, the cells were stained with monodansylcadaverine (0.1 mM) at 37 °C for 30 min. Fluorescence intensity was then measured using a fluorescence microscope (Olympus IX51 with DP70, Olympus America Inc., Center Valley, PA, USA).

### Resazurin reduction assay

The lung cancer cells were treated with various concentrations of Res or SM-3 (0–200 µM) for 24 h. Therefore, the SM-3 treated cells were stained with Resazurin (0.05 mg/mL) and incubated at 37 °C for 3 h. The reduced form of resorufin was measured at 570 nm.

### Measurement of mitochondria function for intracellular ROS

The intracellular level of reactive oxygen species (ROS) was evaluated using fluorescence microscopy. Lung cancer cells were seeded in 96-well plates and exposed to varying concentrations (0–50 µM) of Res or SM-3 for 3 h. Following treatment, the cells were incubated with 15 µM DCFH2-DA for 30 min at 37 °C protect from light. Finally, the stained cells were imaged using a fluorescence microscope (Olympus IX51 with a DP70 digital camera, Olympus, Tokyo, Japan).

### JC-1 staining for mitochondria membrane potential

Lung cancer cells were exposed to different concentrations of Res or SM-3 (0–50 µM) for 24 h. Following treatment, the medium was removed from SM-3-treated cells, and they were rinsed with PBS. Subsequently, cells treated with Res or SM-3 were stained with 2 µM JC-1 for 20 min at 37 °C protect from light. Finally, JC-1-stained cells were imaged using a fluorescence microscope (Olympus IX51 with a DP70 digital camera, Olympus, Tokyo, Japan).

### Three-dimensional (3D) spheroids formation assay

A three-dimensional (3D) spheroids formation assay was conducted to evaluate the existence of CSCs subpopulations in lung cancer cells. Lung cancer cell lines, including A549, H292, and H460, were cultured in 24-well ultra-low attachment plates at a density of 1.5 × 10³ cells per well in medium containing 1% FBS for 7 days to generate primary spheroids. Fresh medium with 1% FBS was added every 2 days. After the 7-day period, the primary spheroids were harvested, trypsinized to obtain a single-cell suspension, and then reseeded into 24-well ultra-low attachment plates for an additional 10 days to form secondary spheroids.

The secondary spheroids were placed in Eppendorf tubes, with one single spheroid transferred to 96-well ultra-low attachment plates. These spheroids were treated with SM-3 (50 µM) for 3 days, with imaging conducted every 24 h using a microscope. After 3 days, the SM-3 treated spheroids were co-stained with Hoechst 33,342 and propidium iodide (PI) and captured using a fluorescence microscope (Olympus IX51 with a DP70 digital camera, Olympus, Tokyo, Japan).

After 10 days, the secondary spheroids underwent a 24 h treatment with SM-3 (50 µM). The spheroids were fixed with 4% paraformaldehyde in PBS for 20 min and then permeabilized with 0.1% Triton X in PBS for 5 min at room temperature. To block non-specific protein interactions, the spheroids were incubated in a mixture of 5% FBS and 2% BSA in PBS for 1 h at room temperature. Following this, they were incubated with primary antibodies diluted 1:250 in PBS containing 2.5% FBS and 1% BSA overnight at 4˚C.

The next day, the spheroids were rinsed with PBS and incubated with secondary antibodies (Alexa Fluor 594-conjugated goat anti-rabbit IgG (H + L) and Alexa Fluor 488-conjugated goat anti-mouse IgG (H + L)) diluted 1:500 in PBS with 2.5% FBS and 1% BSA for 1 h and 30 min at room temperature. They were then co-stained with Hoechst 33,342 (Sigma, St. Louis, MO, USA) at a concentration of 10 µg/mL for 15 min at room temperature. The fluorescence signals were observed using a fluorescence microscope (Olympus IX51 with DP70, Olympus America Inc., Center Valley, PA), and the fluorescence intensity was quantified using ImageJ software. Spheroids treated with Res served as the positive control in this experiment.

### Organoids 3D culture in vitro

Corning Matrigel Basement Membrane Matrix was thawed overnight by placing the vial in a refrigerator at 4 °C before use. A volume of 200 µL of the Matrigel matrix was added to a pre-chilled 24-well plate and incubated at 37 °C for 30 min to allow it to gel. A single-cell suspension of lung cancer cells, including A549, H292, and H460, was prepared at a density of 2 × 10⁵ cells/mL (250 µL) and placed onto the pre-coated Matrigel matrix in the 24-well plate, then incubated at 37 °C for an additional 30 min. Following this, a cool complete medium mixed with 10% Matrigel matrix (250 µL) was added to the culture plate. The cells were cultured for 4 days to develop organoids, with the Matrigel medium mixture changed every 2 days. The organoids were treated with Res or SM-3 (50 µM) for another 24 h.

After treatment, the medium was removed from the organoids culture, and the organoids were washed twice with PBS. To extract the organoids from the Matrigel, 1 mL of freshly prepared 4% paraformaldehyde was added for 30 min at room temperature, followed by 500 µL of PBS for 15 min at room temperature. This process of alternating washes with paraformaldehyde and PBS was repeated two more times. The fixed organoids were then permeabilized with a blocking buffer (5% FBS + 0.5% Triton X-100 in PBS) for 2 h at room temperature. The primary antibody, diluted 1:200 in the blocking buffer, was added and incubated overnight at 4 °C.

The next day, the organoids were washed three times with PBS and stained with a secondary antibody overnight at 4 °C, prepared at a dilution of 1:250 in the blocking buffer. The following day, the organoids with conjugated secondary antibodies were washed three times with PBS and subjected to nuclear staining with Hoechst 33,342 for 15 min at room temperature. The immunostaining of the 3D organoid cells was captured using a confocal microscope at 40× magnification (Zeiss Microscopy LSM 900; Carl Zeiss, Maple Grove, MN, USA).

### Statistical analysis

Data are expressed as the mean ± standard deviation (SD) from three or more independent biological experiments. Multiple comparisons were performed using one-way ANOVA followed by a post hoc test in GraphPad Prism software version 9.0 (GraphPad Software, La Jolla, CA, USA). A statistically significant difference between group levels was defined as a p-value of less than 0.05.

## Conclusion

This study identifies SM-3 as a promising candidate for mTOR-targeted drug discovery, offering enhanced binding stability and favorable interactions compared to Res. The integration of computational insights with experimental validation suggests that SM-3 could serve as a basis for developing selective and effective mTOR inhibitors for therapeutic use. The modifying Res by substituting a methyl group on the hydroxy group of ring A and introducing a methoxy group at the para position of ring B to create the compound SM-3 results in a more effective targeted suppression of CSCs through the inhibition of the mTOR protein in lung cancer cells. Moreover, SM-3 serves as a targeted therapy that induces autophagy-related cell death and suppresses CSCs by inhibiting mTOR (Fig. 11). This mTOR inhibition promotes autophagic cell death in lung cancer cells while suppressing CSCs traits.

## Electronic supplementary material

Below is the link to the electronic supplementary material.

Supplementary Material 1

Supplementary Material 2

Supplementary Material 3

Supplementary Material 4

Supplementary Material 5

Supplementary Material 6

Supplementary Material 7

Supplementary Material 8

Supplementary Material 9

## Data Availability

Data is provided within the manuscript or supplementary information files.
