# Supplementary material for: Substituents introduction of methyl and methoxy functional groups on resveratrol stabilizes mTOR binding for autophagic cell death induction
Source: Sci Rep. 2025 Apr 26;15:14675. doi: 10.1038/s41598-025-98616-6 (PMC12033263; doi:10.1038/s41598-025-98616-6)
Supplement: Supplementary file 1 — Supplementary Material 1 [file 41598_2025_98616_MOESM1_ESM.pdf]

Supporting information

Substituents introduction of methyl and methoxy functional groups on Resveratrol stabilizes mTOR binding for autophagic cell death induction

Zin Zin Ei[1,2], Satapat Racha[1,2,5], Preedakorn Chunhacha[2,6], Masashi Yokoya[7], Sohsuke Moriue[7], Hongbin Zou[8], Pithi Chanvorachote[1,2,3,4*]

[1]Department of Pharmacology and Physiology, Faculty of Pharmaceutical Sciences, Chulalongkorn university, Bangkok 10330, Thailand.
[2]Center of Excellence in Cancer Cell and Molecular Biology, Faculty of Pharmaceutical Sciences, Chulalongkorn University, Bangkok 10330, Thailand.
[3]Faculty of Pharmacy, Silpakorn University, Nakhon Pathom, Thailand.
[4]Sustainable Environment Research Institute, Chulalongkorn University, Bangkok, Thailand.
[5]Interdisciplinary Program in Pharmacology, Graduate School, Chulalongkorn university, Bangkok 10330, Thailand.
[6]Department of Biochemistry and Microbiology, Faculty of Pharmaceutical Sciences, Chulalongkorn University, Bangkok, Thailand.
[7]Department of Pharmaceutical Chemistry, Meiji Pharmaceutical University, 2-522-1, Noshio, Kiyose, Tokyo 204-8588, Japan.
[8]College of Pharmaceutical Sciences, Zhejiang University, Hangzhou 310058, China.

*Correspondence: pithi_chan@yahoo.com or pithi.c@chula.ac.th; Tel.: +66-2218-8344

^{1}H-NMR of **2b** in $CDCl_3$ (400 MHz)

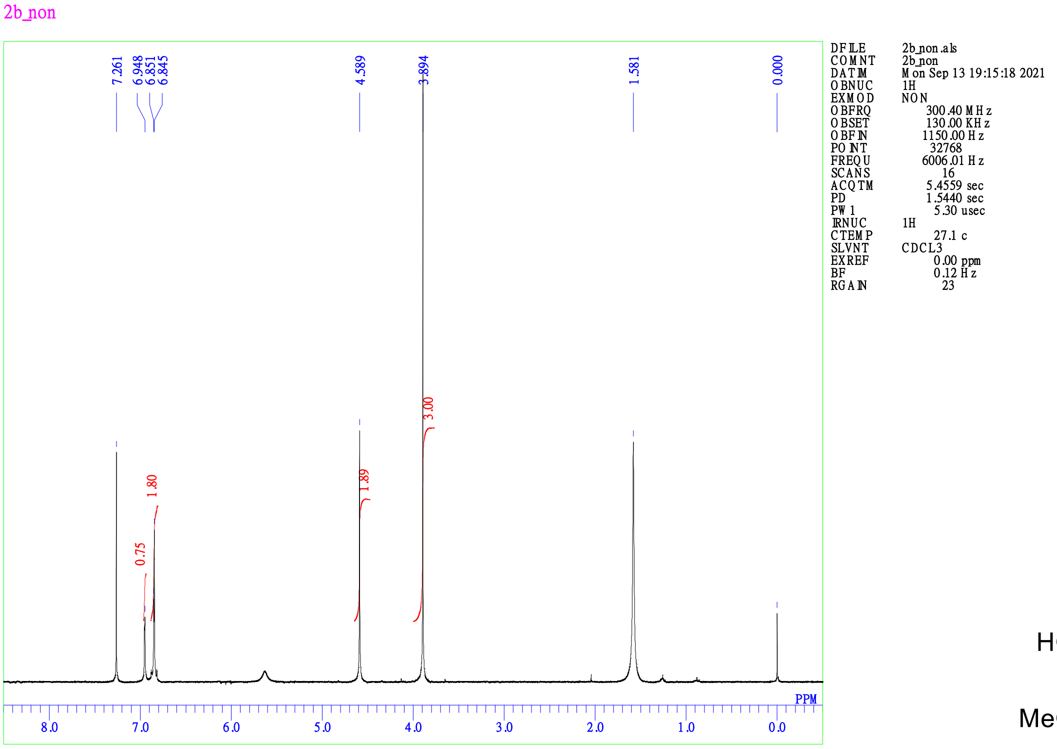

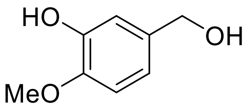

2b

^{1}H-NMR of **3b** in $CDCl_3$ (400 MHz)

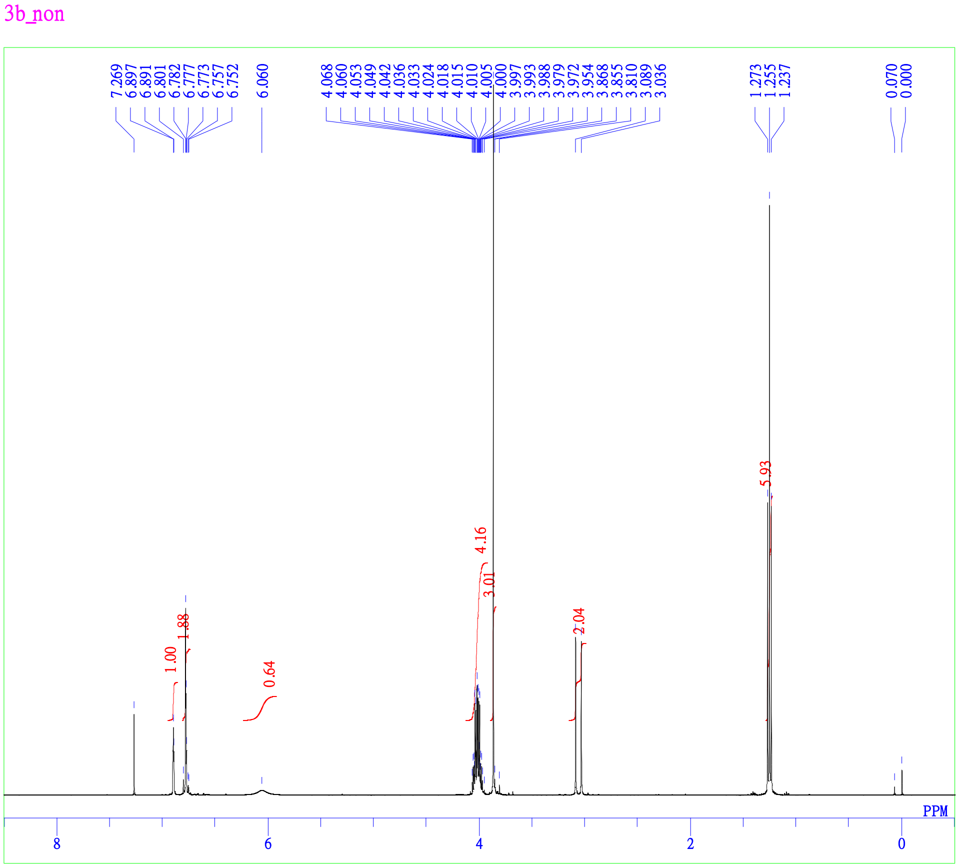

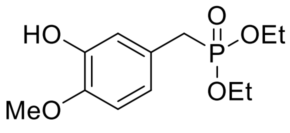

3b

^{1}H-NMR of **5** in $CDCl_3$ (400 MHz)

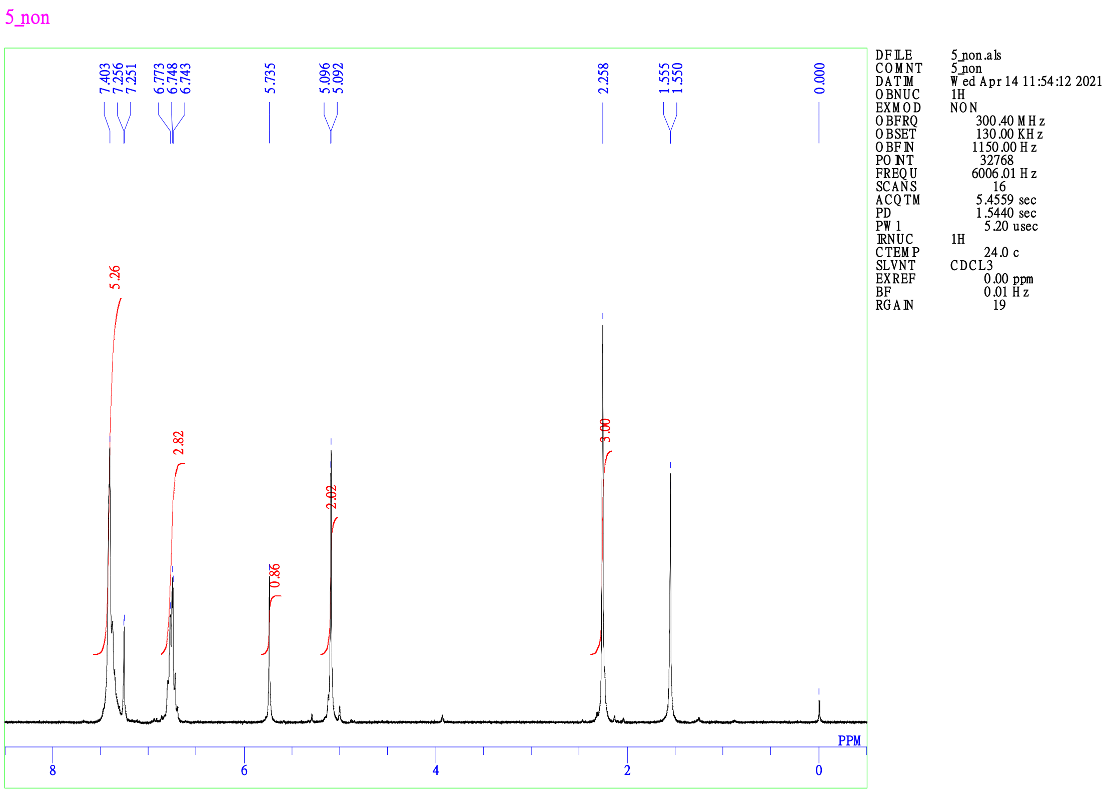

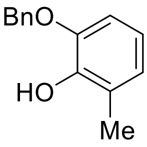

5

^{1}H-NMR of **6** in $CDCl_3$ (400 MHz)

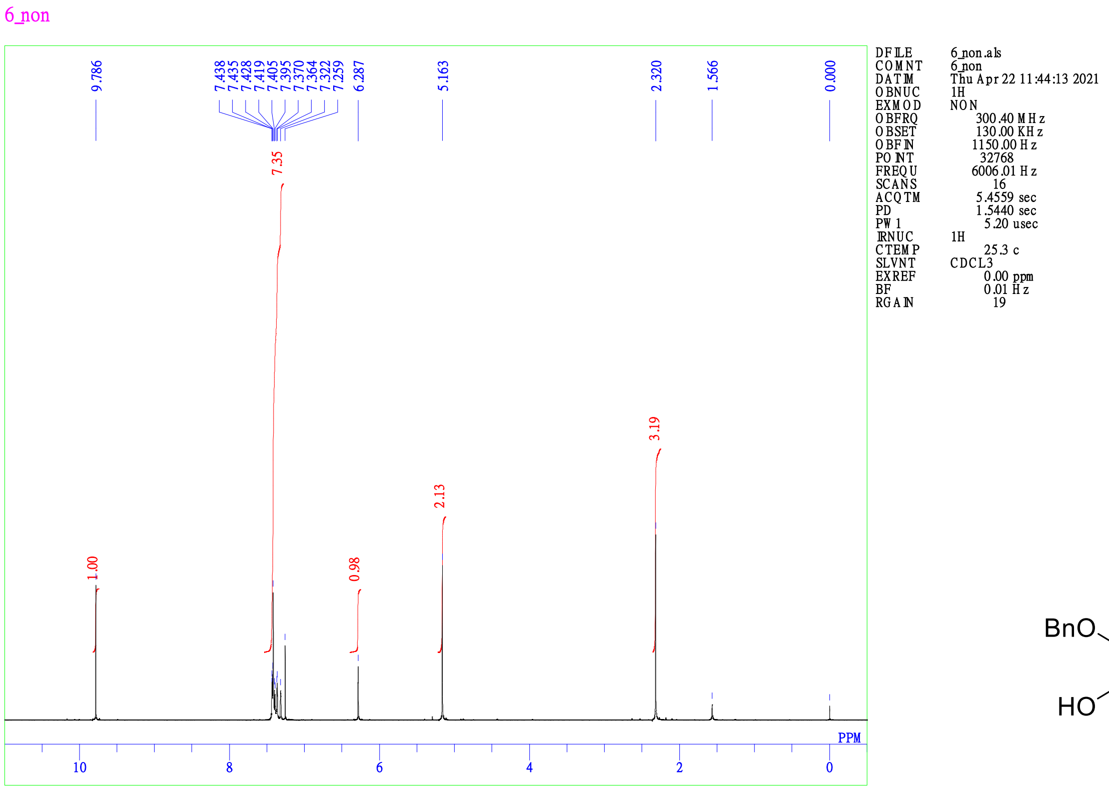

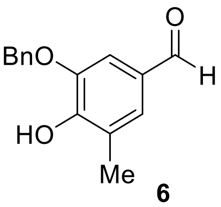

6

^{1}H-NMR of **1d** in $CDCl_3$ (400 MHz)

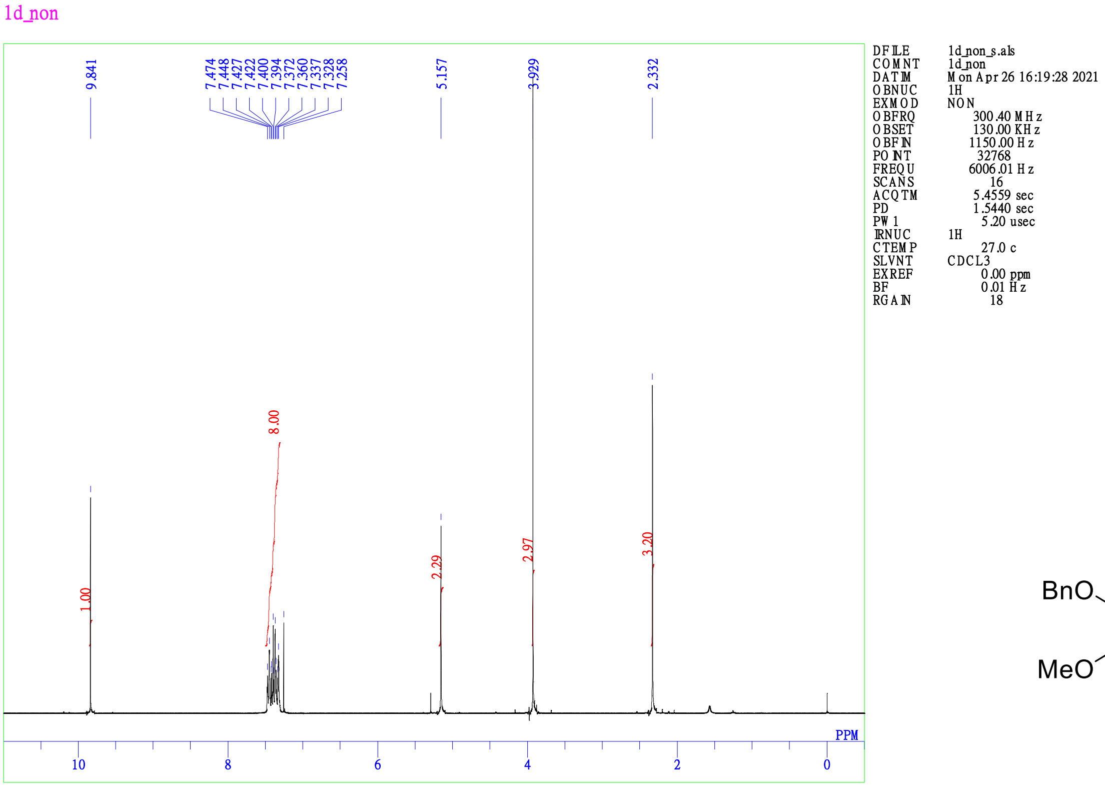

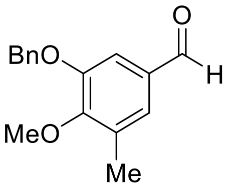

1d

^{1}H-NMR of **2d** in $CDCl_3$ (400 MHz)

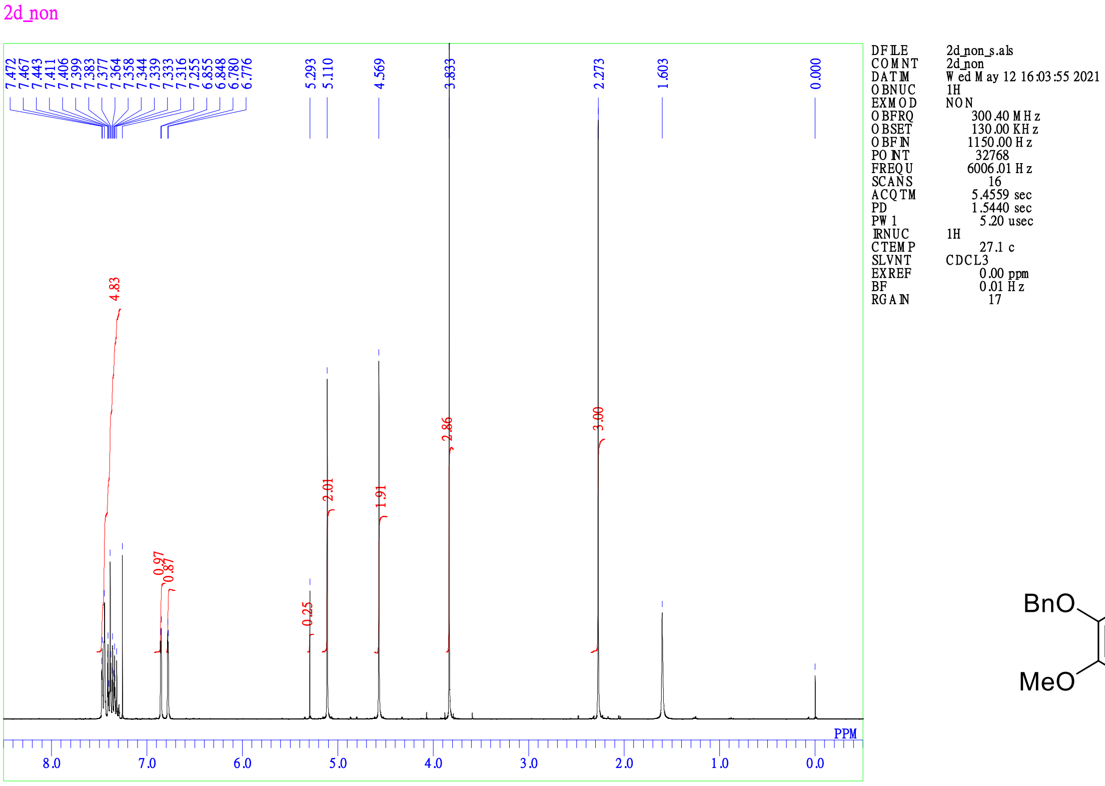

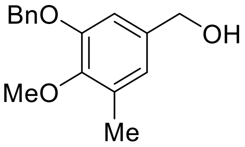

2d

^{1}H-NMR of **3d** in $CDCl_3$ (400 MHz)

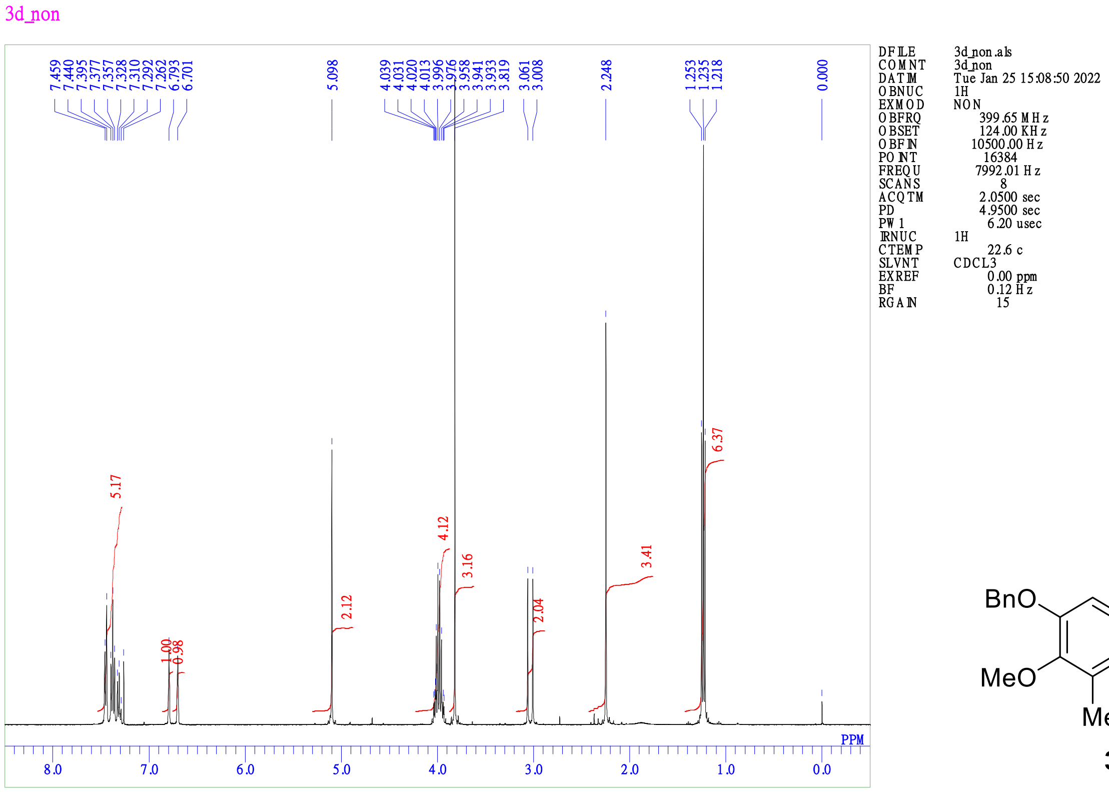

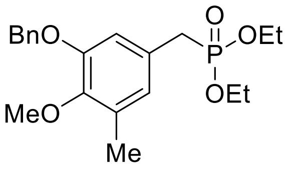

3d

^{13}C-NMR of **3d** in $CDCl_3$ (100 MHz)

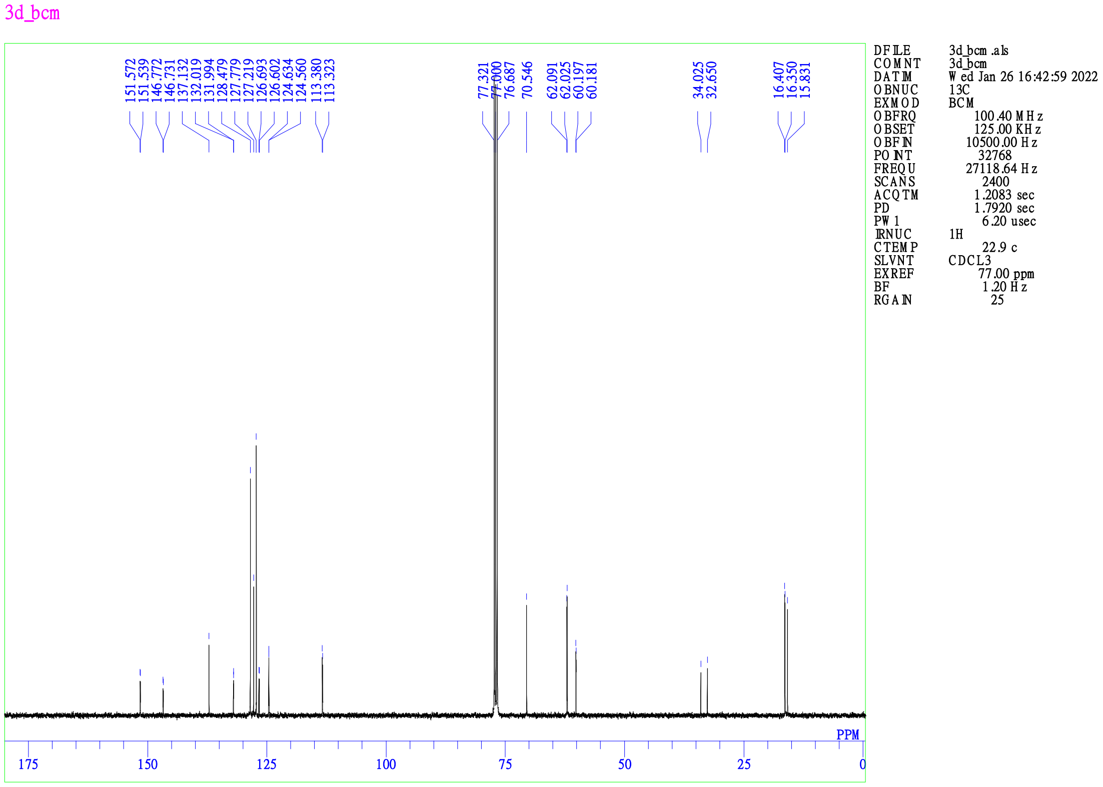

^{1}H-NMR of **7a** in $CDCl_3$ (400 MHz)

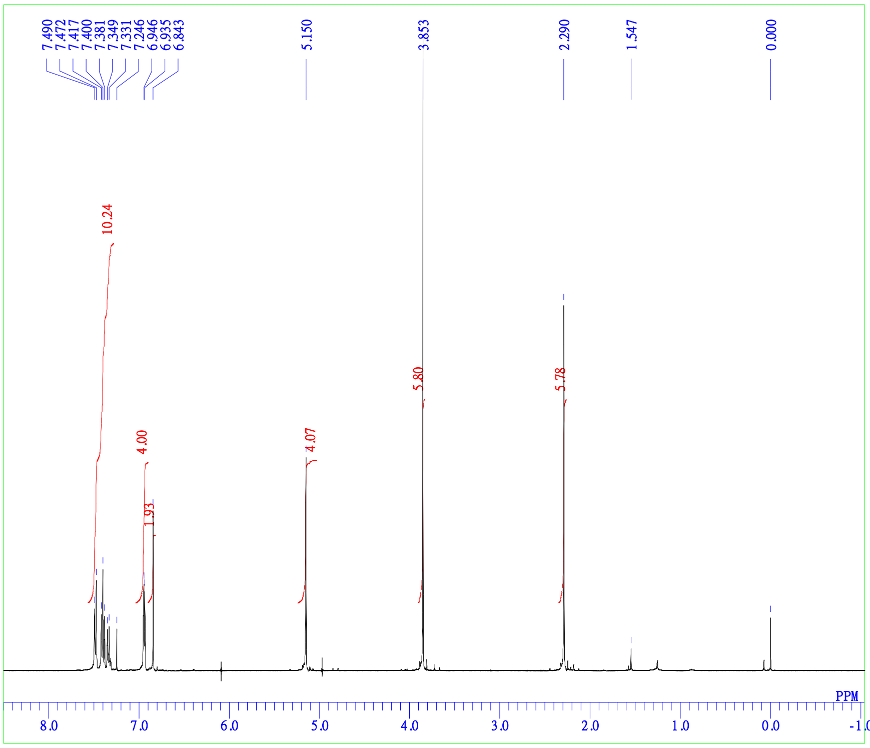

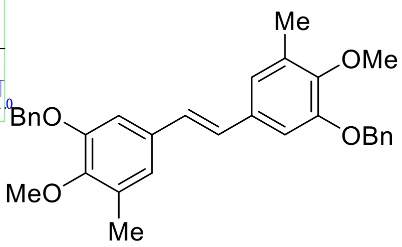

7a

^{13}C-NMR of **7a** in $CDCl_3$ (100 MHz)

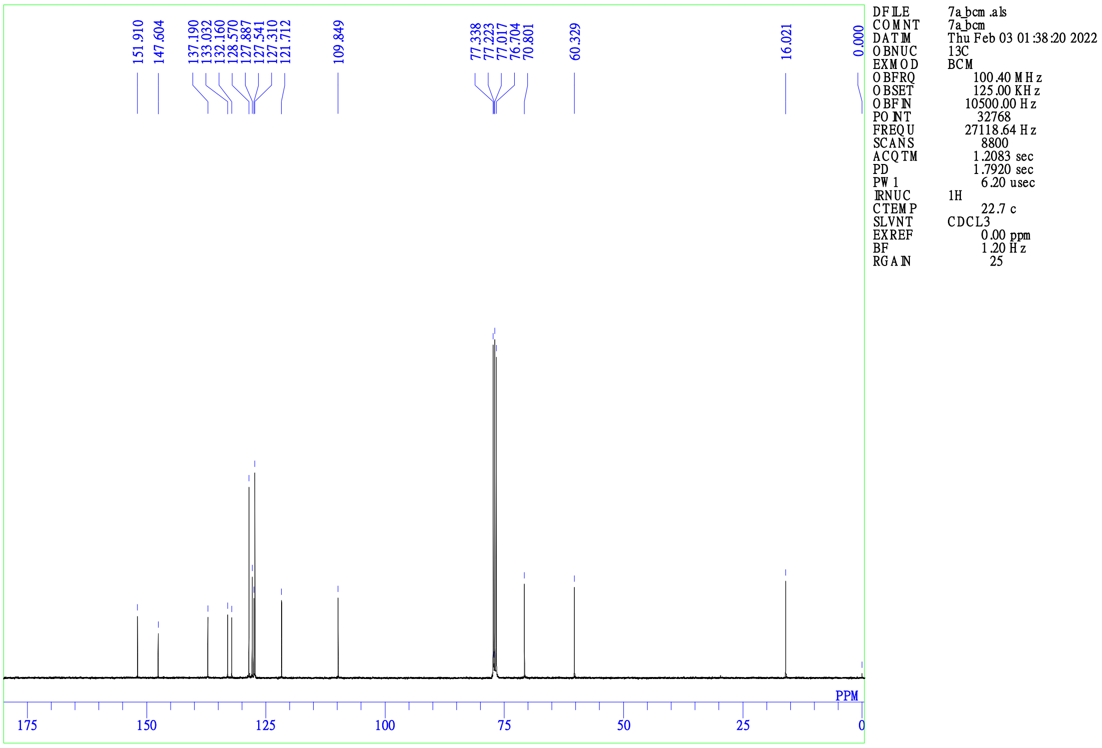

^{1}H-NMR of **8a (SM-1)** in $CDCl_3$ (400 MHz)

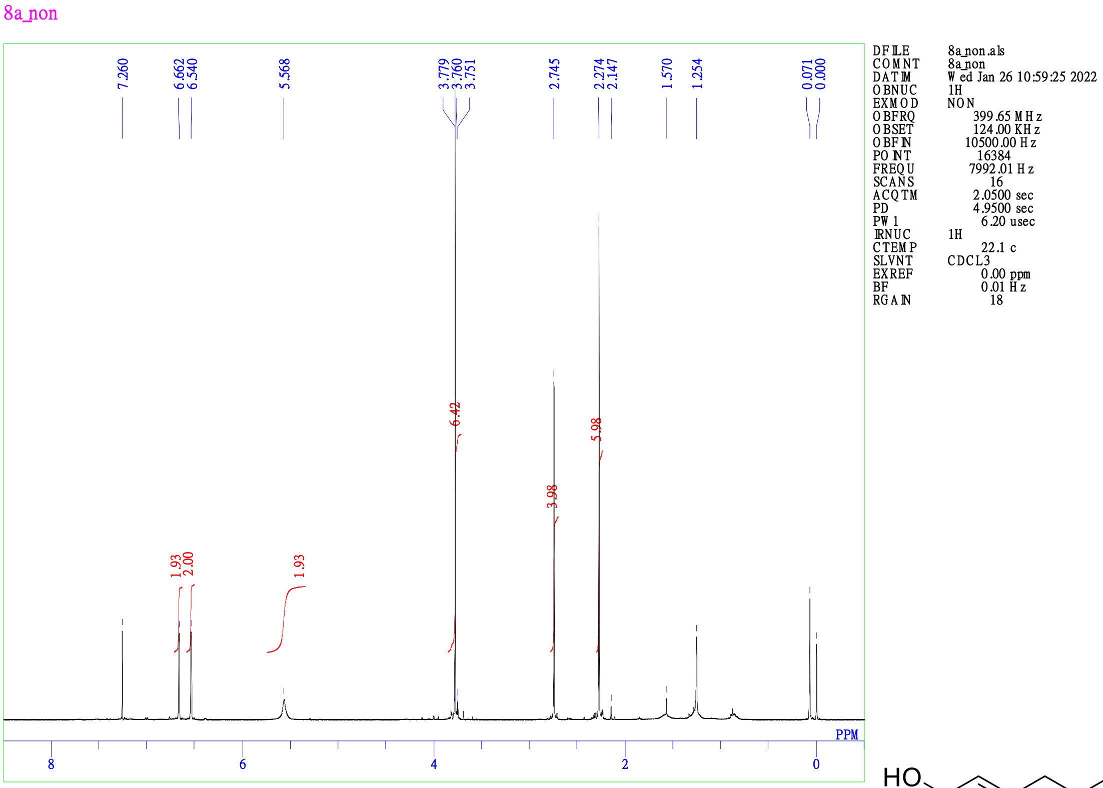

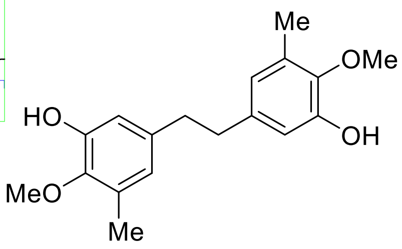

8a (SM-1)

^{13}C-NMR of **8a (SM-1)** in $CDCl_3$ (100 MHz)

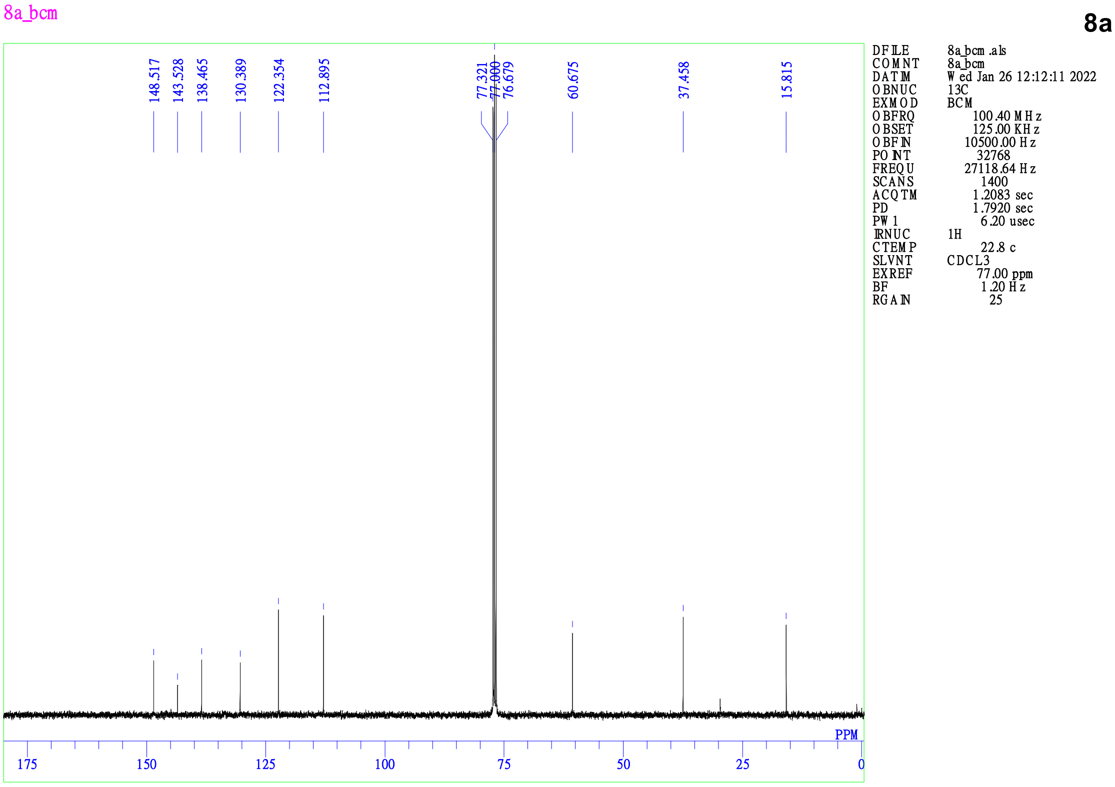

^{1}H-NMR of **7b** in $CDCl_3$ (400 MHz)

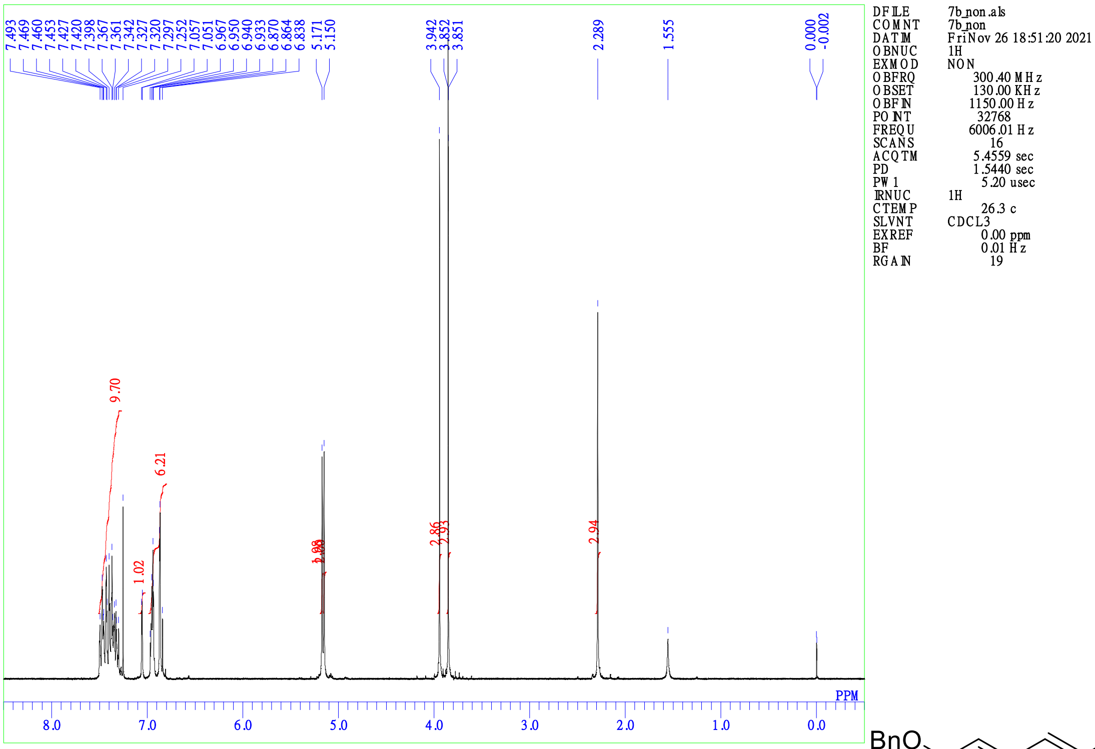

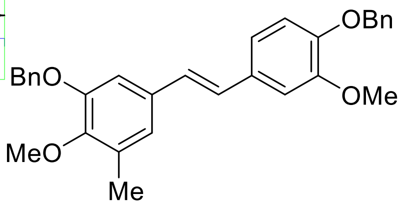

7b

^{13}C-NMR of **7b** in $CDCl_3$ (400 MHz)

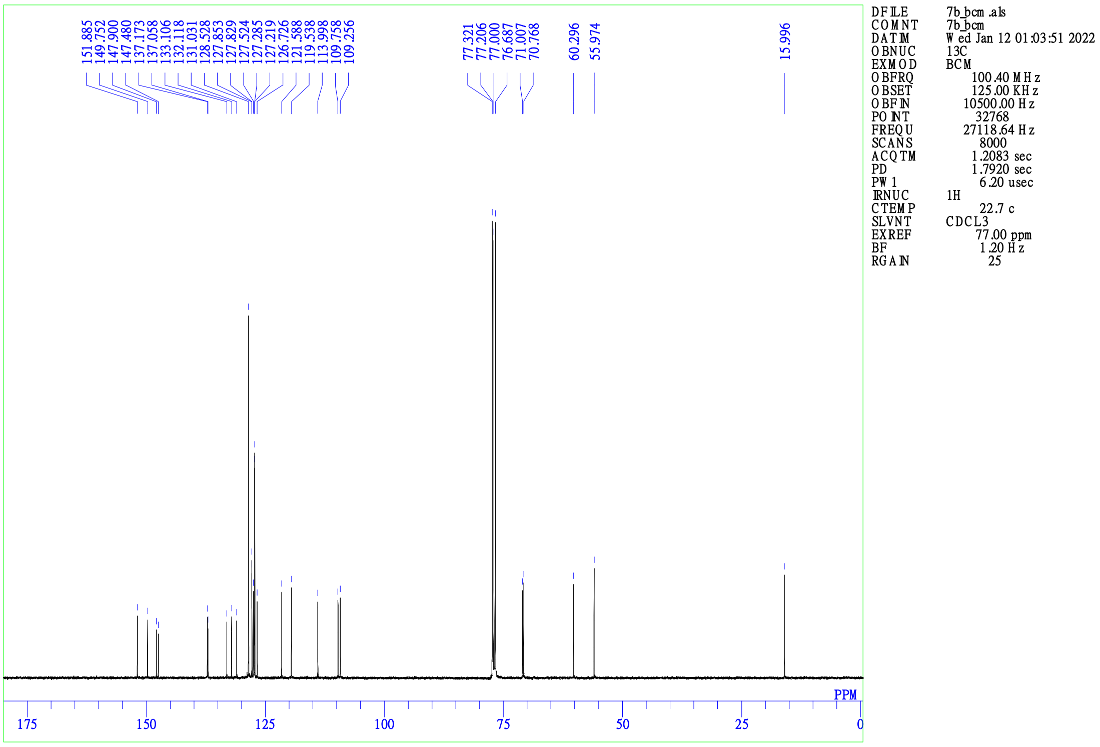

^{1}HNMR of **8b (SM-2)** in $CDCl_3$ (100 MHz)

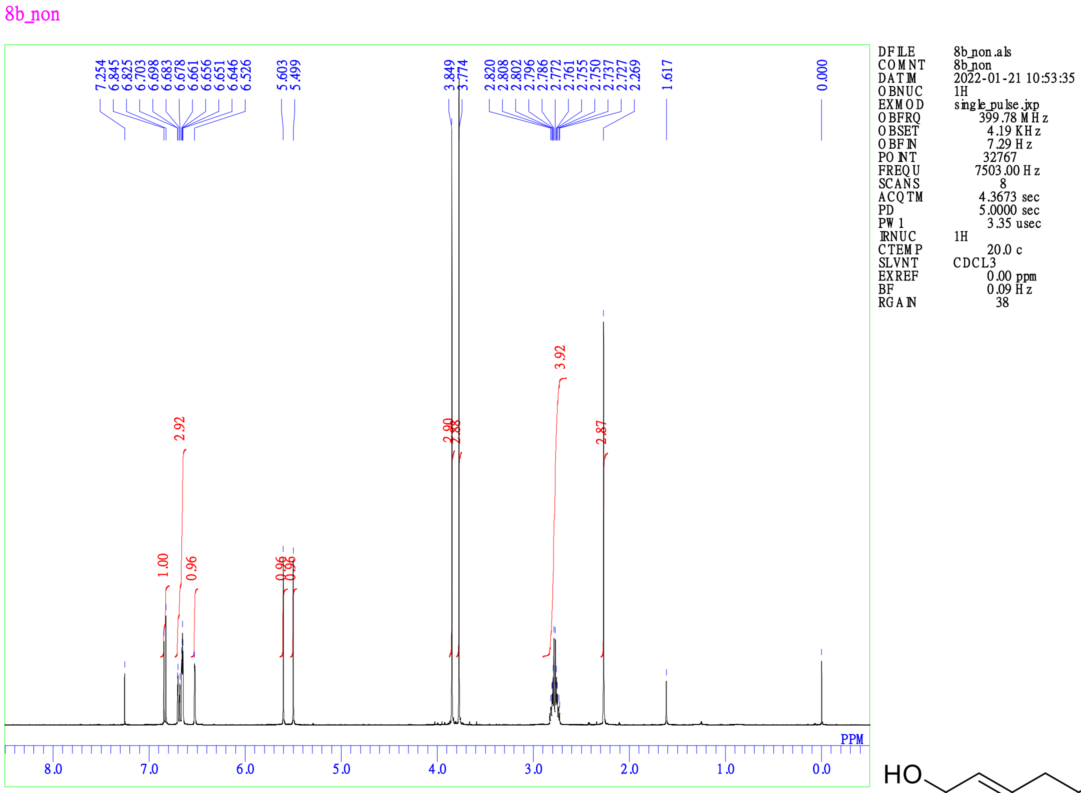

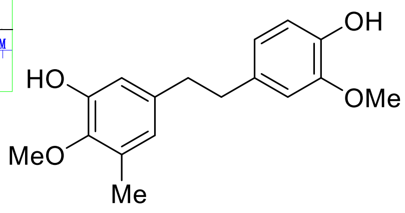

8b (SM-2)

^{13}C-NMR of **8b (SM-2)** in $CDCl_3$ (100 MHz)

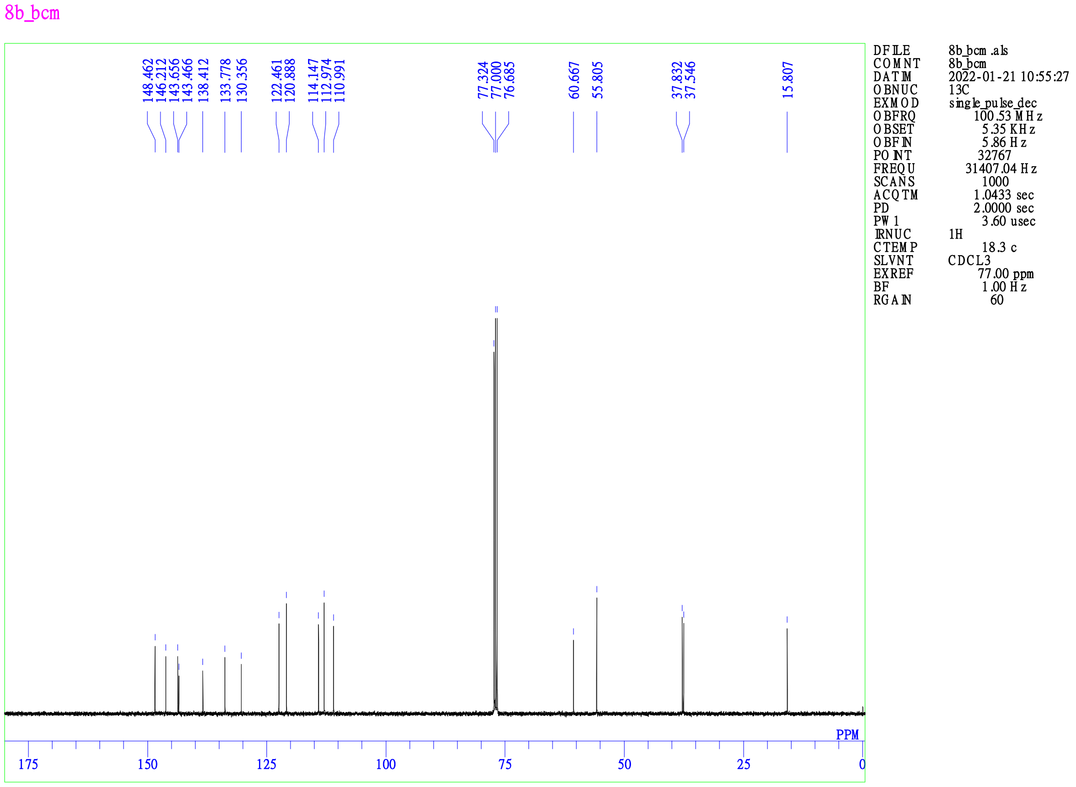

^{1}H-NMR of **7c** in $CDCl_3$ (400 MHz)

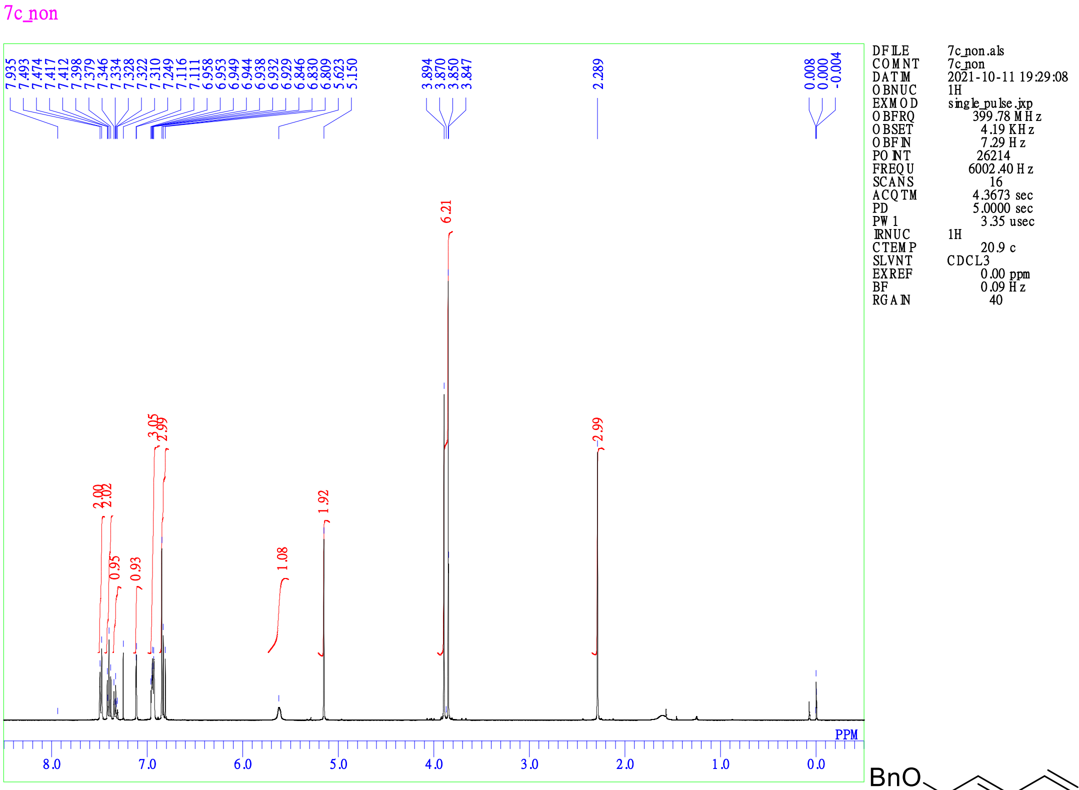

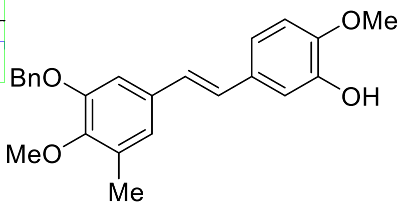

7c

^{13}C-NMR of **7c** in $CDCl_3$ (400 MHz)

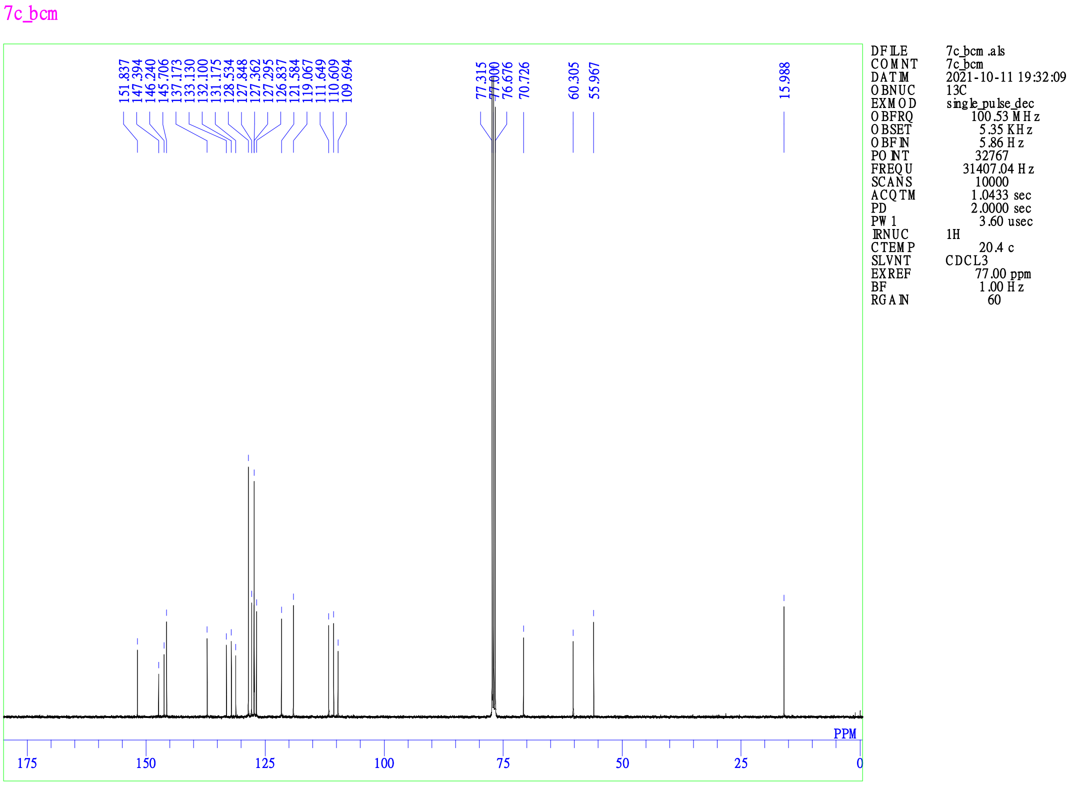

^{1}H-NMR of **8c (SM-3)** in $CDCl_3$ (100 MHz)

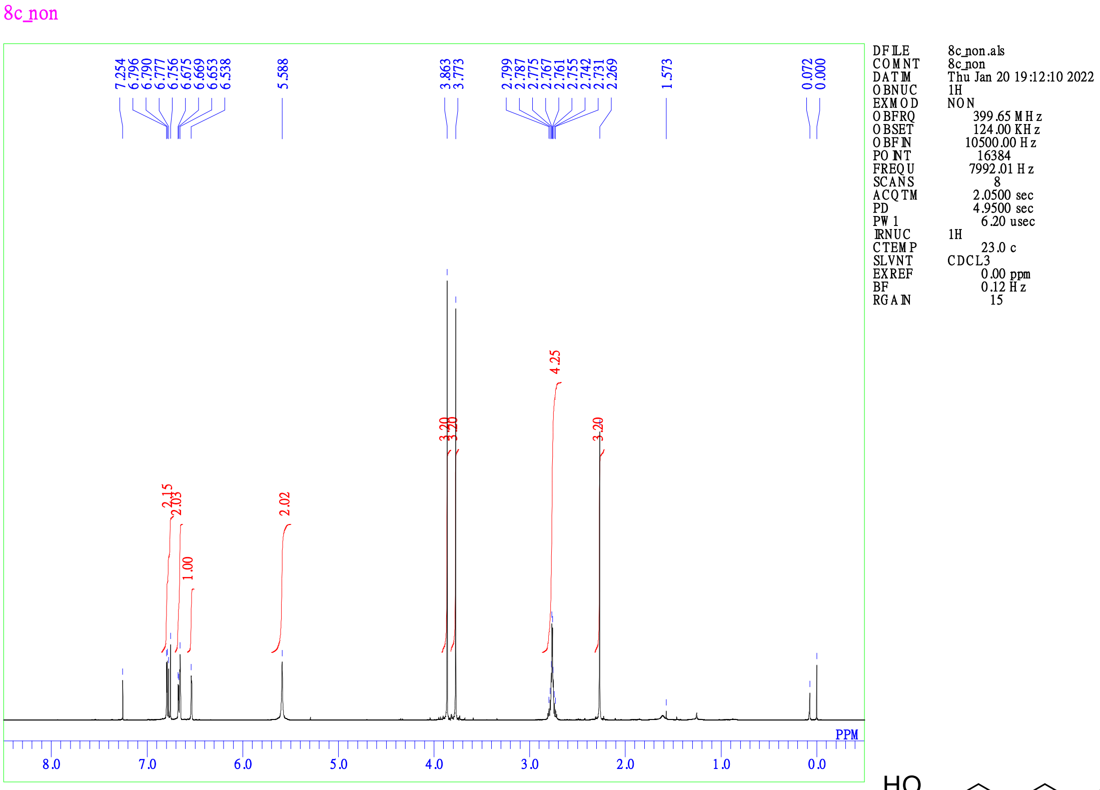

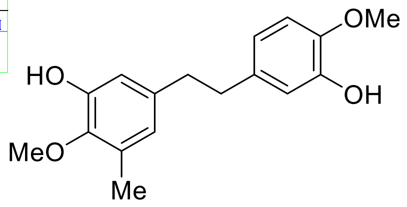

8c (SM-3)

^{13}C-NMR of **8c (SM-3)** in $CDCl_3$ (100 MHz)

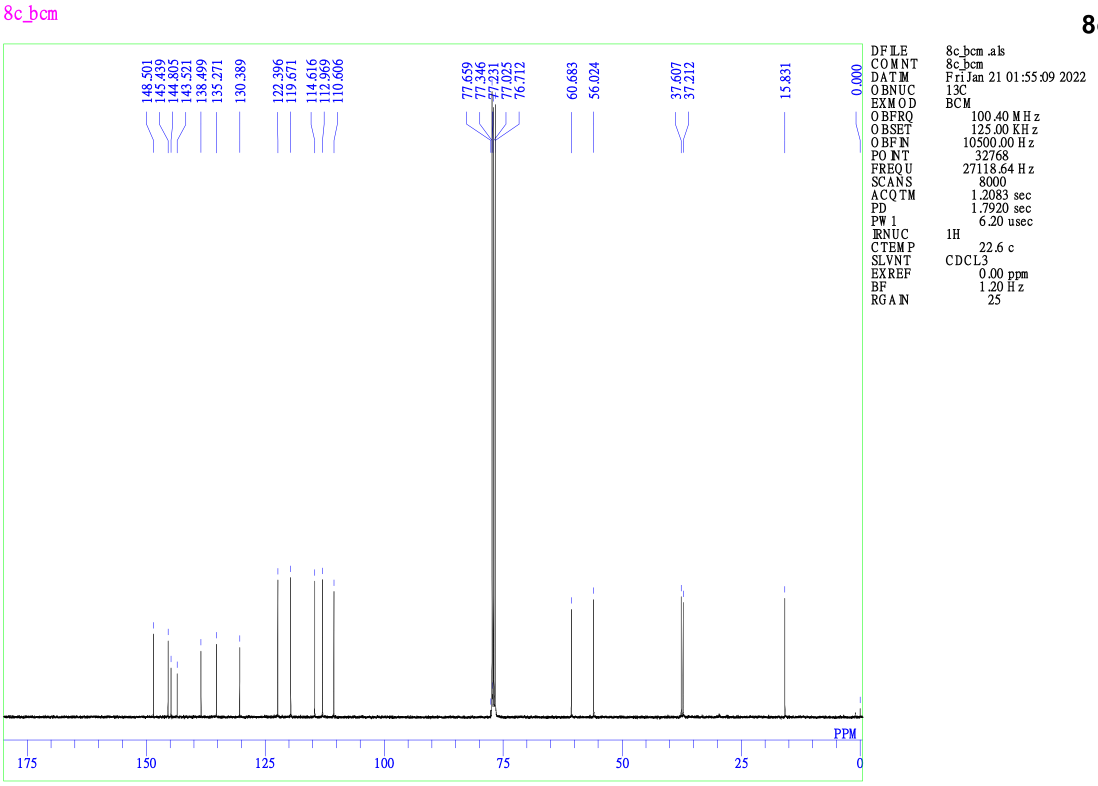

^{1}H-NMR of **7d** in $CDCl_3$ (400 MHz)

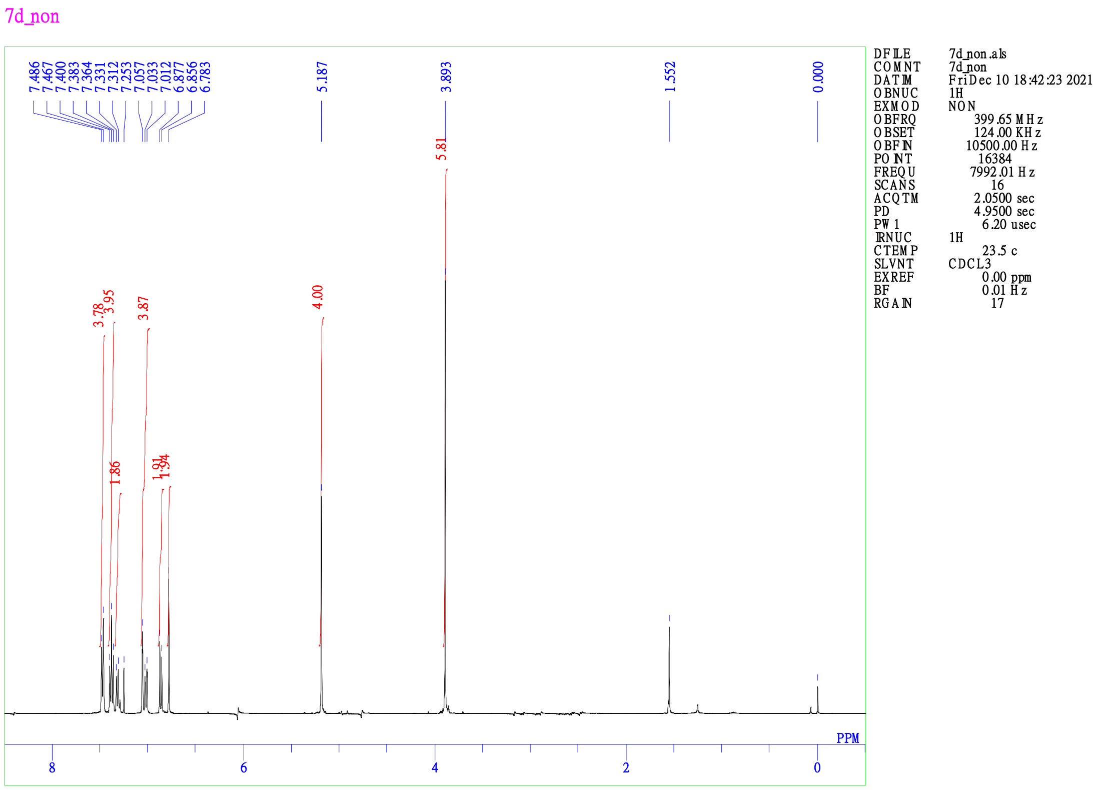

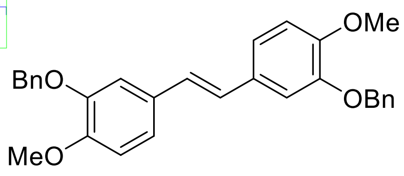

7d

^{13}C-NMR of **7d** in $CDCl_3$ (100 MHz)

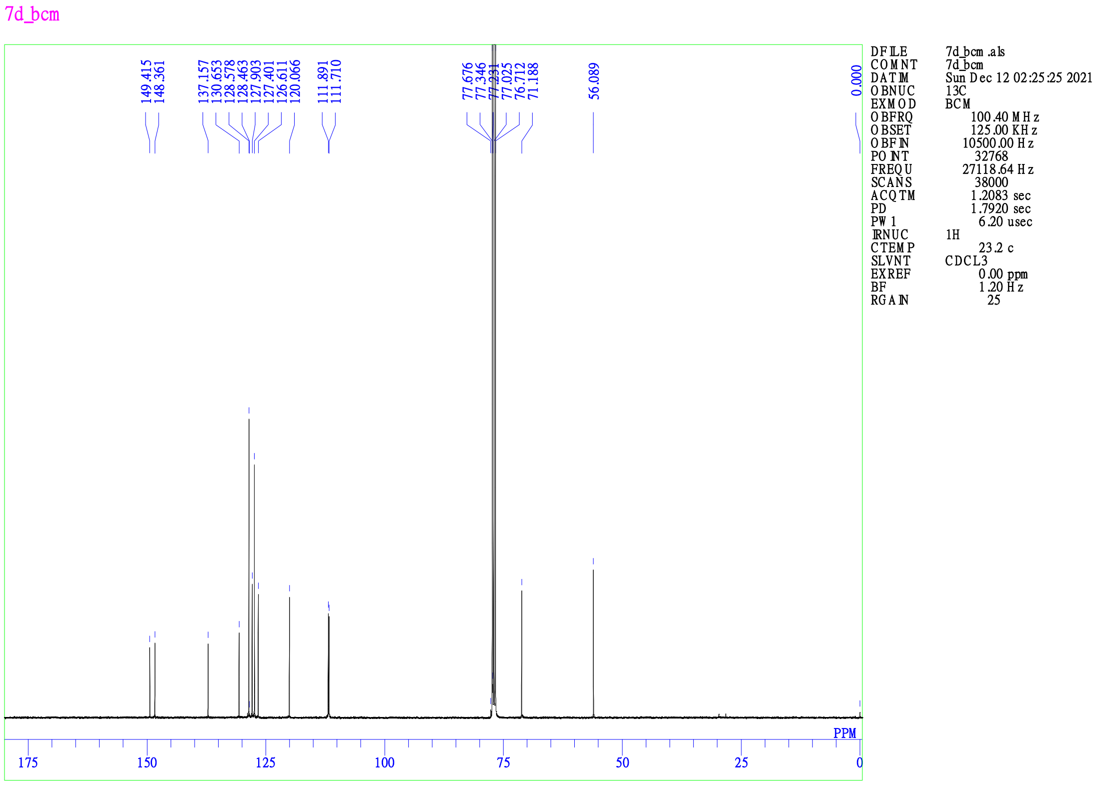

^{1}H-NMR of **8d (SM-8)** in $CDCl_3$ (400 MHz)

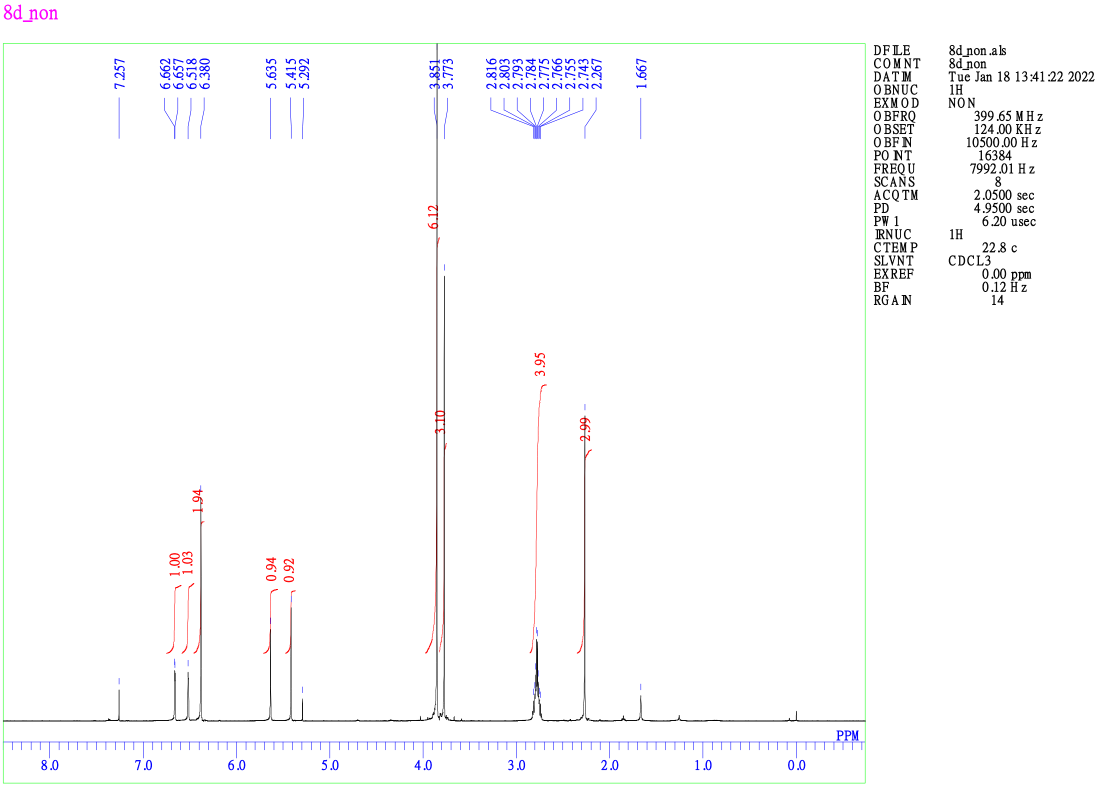

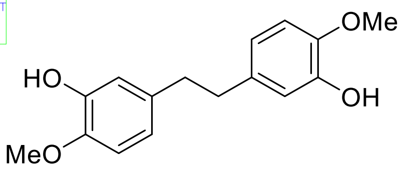

8d (SM-8)

^{13}C-NMR of **8d (SM-8)** in $CDCl_3$ (100 MHz)

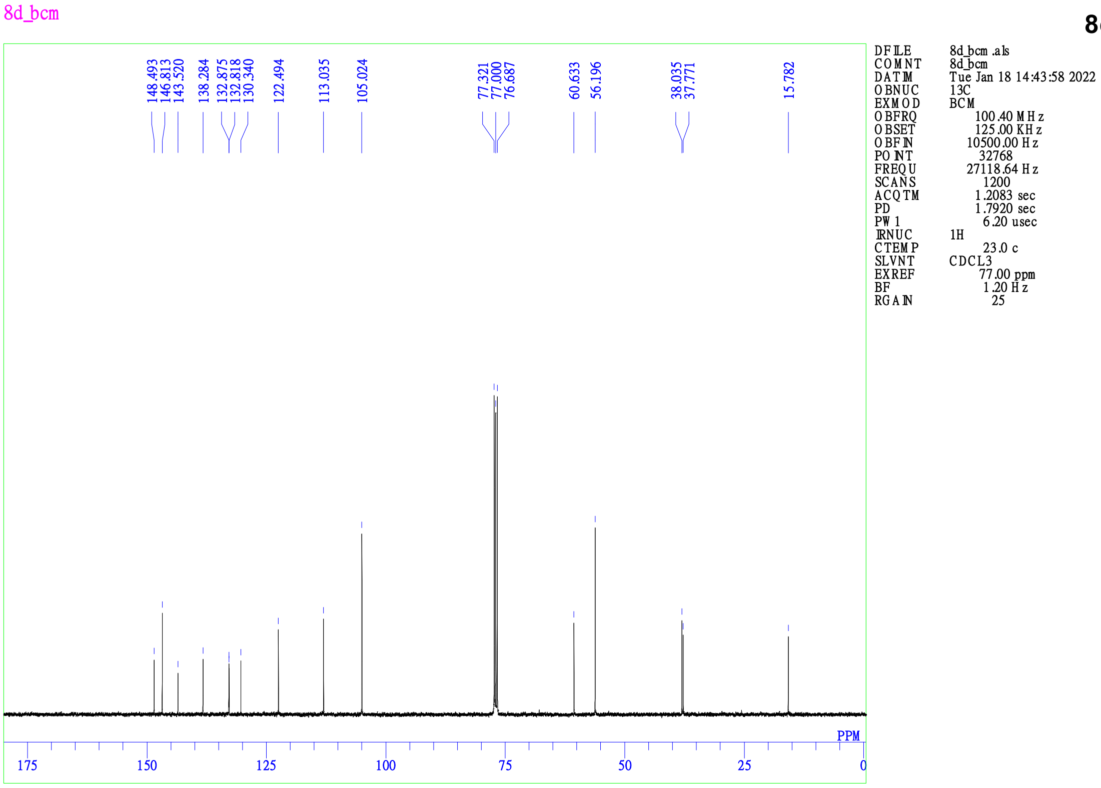

Table S1. Evaluation of Lipinski's Rule and PAINS Liabilities for Compounds

Compounds	SMILES	Lipinski	PAINS
Res	C1=CC(=CC=C1/C=C/C2=CC(=CC(=C2)O)O)O	Yes	0 alert
SM-3	COC1=C(O)C=C(CCC2=CC(O)=C(OC)C(C)=C2)C=C1	Yes	0 alert

Table S2. Hydrogen bond lifetime profiles of mTOR-Res and mTOR-SM-3

Systems	Acceptor	DonorH	Donor	Frames	%
mTOR-Res	ASP2195@OD2	Res@H6	Res@O1	7,802	39.01
	ASP2195@OD1	Res@H6	Res@O1	5,596	27.98
	Res@O1	TYR_2225@HH	TYR_2225@OH	5,317	26.58
	VAL2240@O	Res@H	Res@O	5,167	25.84
	ASP2357@OD1	Res@H8	Res@O2	2,063	10.32
mTOR-SM-3	GLU2190@OE1	SM-3@H13	SM-3@O3	10,850	54.25
	SM-3@O	VAL2240@H	VAL2240@N	5,816	29.08
	GLU2190@OE2	SM-3@H13	SM-3@O3	2,471	12.35

(A)

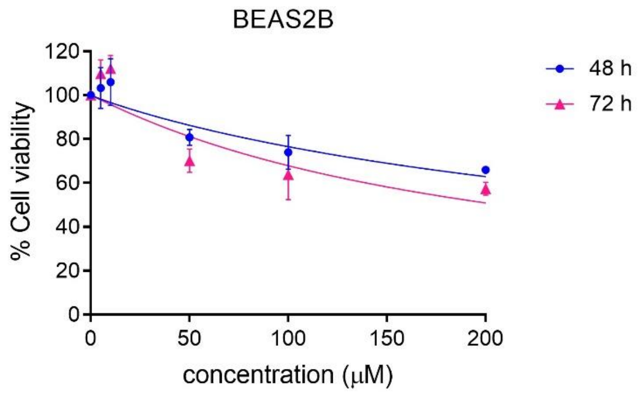

	BEAS2B IC_{50} (μM)±SD (μM)
SM-3_48 h	349.0 ± 0.43
SM-3_72 h	206.9 ± 0.53

Figure S1. (A) The effectiveness and selectivity profile of SM-3 based on IC_{50} values in BEAS2B cells at 48 h and 72 h

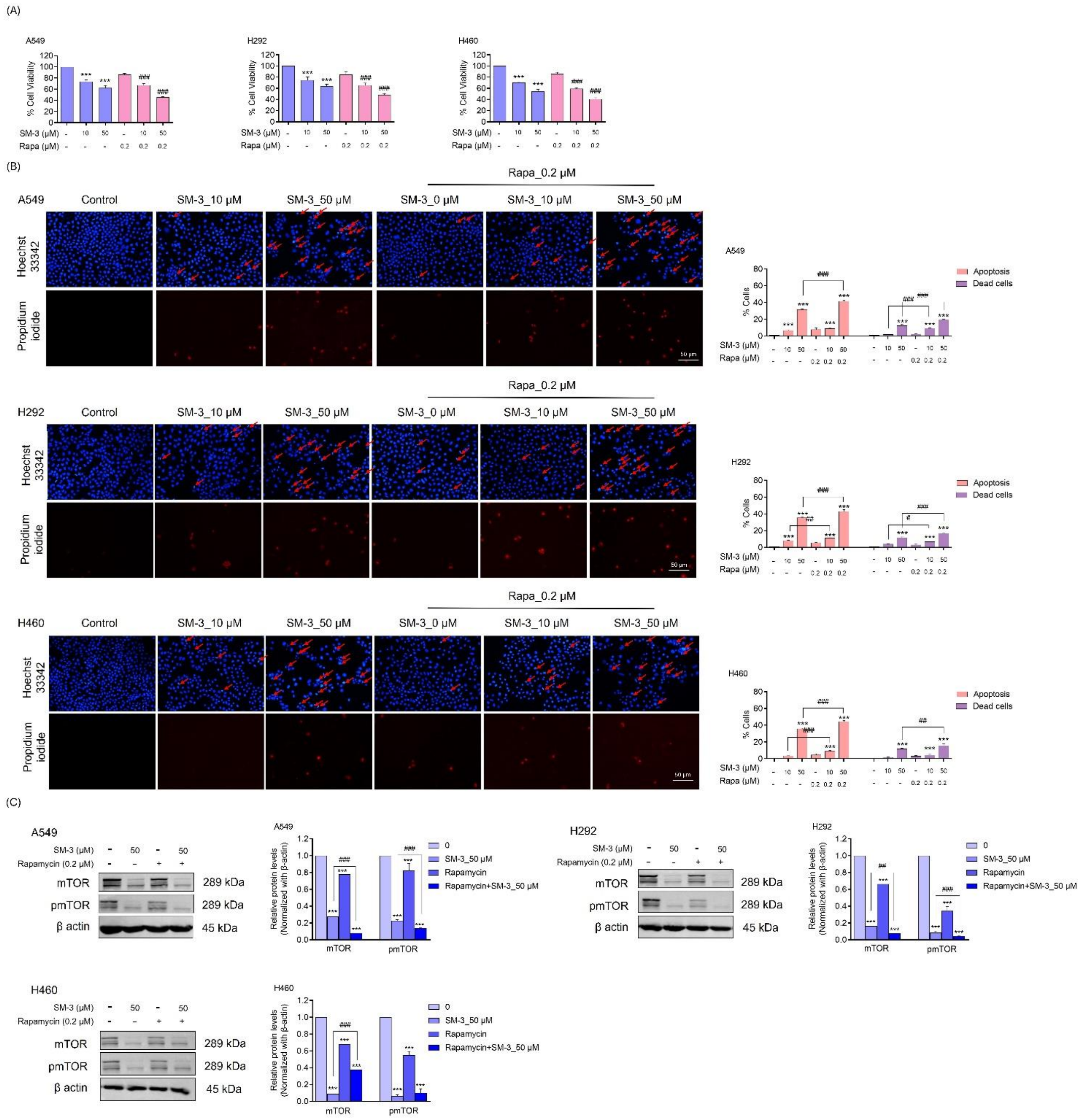

Figure S2. Evaluate the effect of SM-3 on cell viability and cell death induction in NSCLC cells in present of mTORC1 inhibitor, Rapamycin. NSCLC cells were seeded and pretreatment with Rapamycin (0.2 μM) for 1 h treatment with 0-50 μM SM-3 for 24 h treatment. (A) After that cell viability was determined by MTT assay. (B) Double staining with Hoechst 33342 and propidium iodide (PI) to identify chromatin condensation or nuclear fragmentation for apoptosis and necrotic cells. Scale bar: 50 μm (magnification: 20x) (C) Western blot analysis was performed to detect protein levels for mTOR and pmTOR (Ser2448). β-actin act as the loading control. Data are presented as mean ± SD (n = 3), with significance indicated as ***$p < 0.001$ compared to untreated control cells, $^{\#}p < 0.05$, $^{\#\#}p < 0.01$, $^{\#\#\#}p < 0.001$ compared to SM-3 treatment alone.

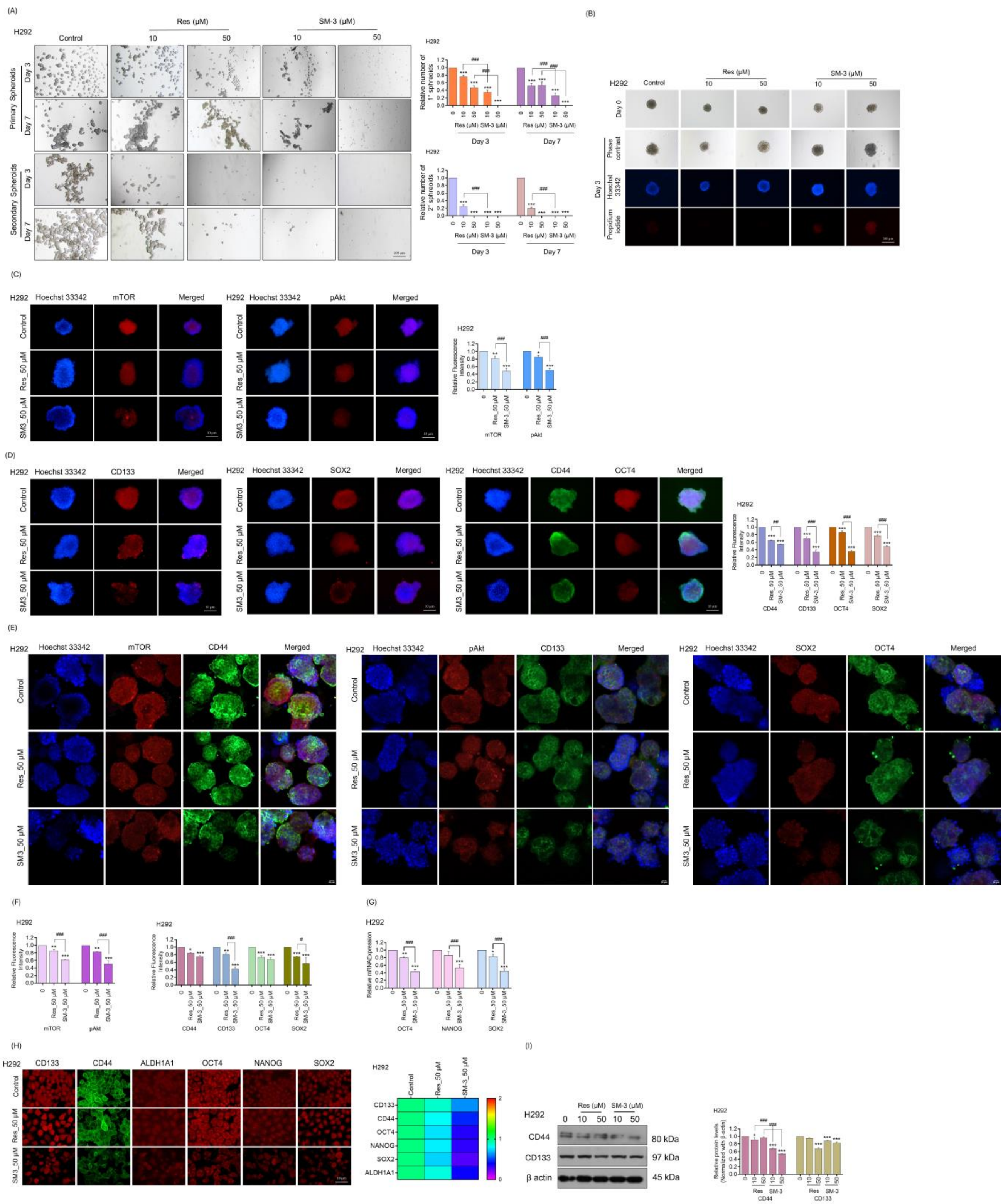

Figure S3. Suppressive effects of SM-3 on CSCs markers via mTOR inhibition during spheroids and organoids formation in H292 cells. (A) The H292 cells were treated with SM-3 (50 μM) and observed using a phase-contrast microscope after 3 and 7 days of incubated primary and secondary spheroids. The relative number of spheroids were analyzed using ImageJ software. Scale bar: 100 μm (magnification: 4x) (B) Single spheroids from a CSCs-rich population of H292 cells were treated with

SM-3 (50 µM) for 3 days, and CSCs viability was evaluated using Hoechst 33342/PI double staining. Scale bar: 50 µm (magnification: 20x) (C, D) The CSCs-rich spheroids were treated with SM-3 (50 µM) for 24 h. The levels of upstream proteins mTOR and pAkt in SM-3 treated CSCs-rich spheroids were assessed using immunofluorescence. The SM-3 treated H292 spheroids were examined for the expression of stem cell markers, as well as the transcription factors. Scale bar: 50 µm (magnification: 20x) (E) SM-3 targeted CSCs by inhibiting mTOR during 3D organoids formation in H292 cells, as analyzed by immunofluorescence. The expression levels of pAkt (Ser473), mTOR, CSCs-rich transcription factors (CD133 and CD44), and stem cell markers (OCT4 and SOX2) were measured in SM-3-treated organoids. Scale bar: 20 µm (magnification: 40x) by confocal microscope (F) The fluorescence intensity was measured by image J software. (G) Cells were treated with SM-3 (50 µM), and the mRNA expression levels of the stem cell transcription factors OCT4, NANOG, and SOX2 were measured. The mRNA levels were normalized against the housekeeping gene GAPDH, and relative mRNA expression was calculated using comparative Ct cycles. (H) The heat map displays the fluorescence intensity of stem cell markers and stem cell transcription factors analyzed by using Image J software. Scale bar: 10 µm (magnification: 40x) (I) The protein expression levels of stem cell markers were assessed using western blot analysis, with the blot reprobed for β-actin to ensure equal protein loading. The parent compound Res (50 µM) served as the positive control. Data are presented as mean ± SD (n = 3). Significance is indicated as *p < 0.05, **p < 0.01, ***p < 0.001 compared to untreated control cells, and $^{\#}p < 0.05$, $^{\#\#}p < 0.01$, $^{\#\#\#}p < 0.001$ compared to Res-treated H292 cells.

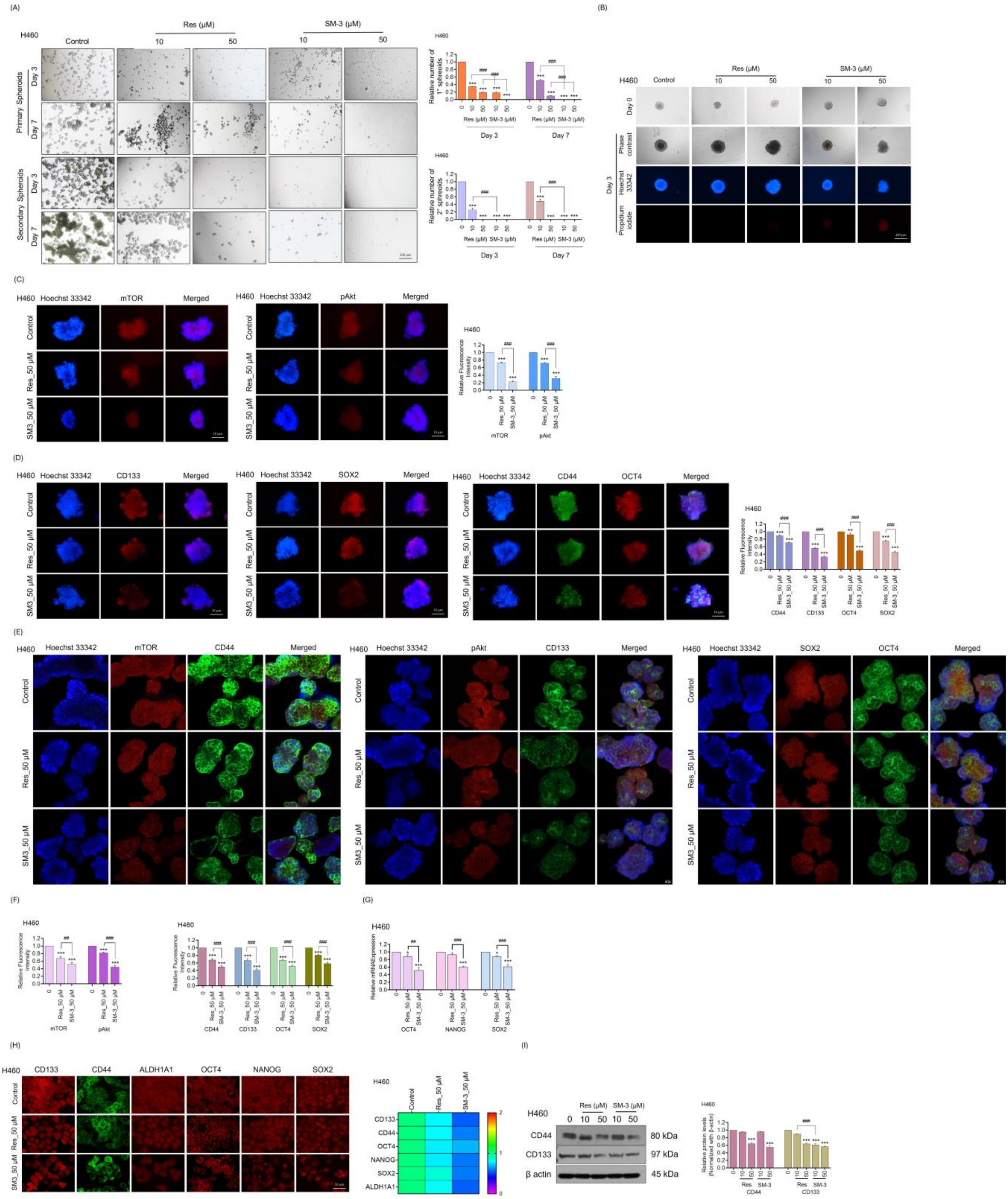

Figure S4. Suppressive effects of SM-3 on CSCs markers via mTOR inhibition during spheroids and organoids formation in H460 cells. (A) The H460 cells were treated with SM-3 (50 μM) and observed using a phase-contrast microscope after 3 and 7 days of incubated primary and secondary spheroids. The relative number of spheroids were analyzed using ImageJ software. Scale bar: 100 μm (magnification: 4x) (B) Single spheroids from a CSCs-rich population of H460 cells were treated with

SM-3 (50 µM) for 3 days, and CSCs viability was evaluated using Hoechst 33342/PI double staining. Scale bar: 50 µm (magnification: 20x) (C, D) The CSCs-rich spheroids were treated with SM-3 (50 µM) for 24 h. The levels of upstream proteins mTOR and pAkt in SM-3 treated CSCs-rich spheroids were assessed using immunofluorescence. The SM-3 treated H460 spheroids were examined for the expression of stem cell markers, as well as the transcription factors. Scale bar: 50 µm (magnification: 20x)(E) SM-3 targeted CSCs by inhibiting mTOR during 3D organoids formation in H460 cells, as analyzed by immunofluorescence. The expression levels of pAkt (Ser473), mTOR, CSCs-rich transcription factors (CD133 and CD44), and stem cell markers (OCT4 and SOX2) were measured in SM-3-treated organoids. Scale bar: 20 µm (magnification: 40x) by confocal microscope (F) The fluorescence intensity was measured by image J software. (G) Cells were treated with SM-3 (50 µM), and the mRNA expression levels of the stem cell transcription factors OCT4, NANOG, and SOX2 were measured. The mRNA levels were normalized against the housekeeping gene GAPDH, and relative mRNA expression was calculated using comparative Ct cycles. (H) The heat map displays the fluorescence intensity of stem cell markers and stem cell transcription factors analyzed by using Image J software. Scale bar: 10 µm (magnification: 40x) (I) The protein expression levels of stem cell markers were assessed using western blot analysis, with the blot reprobed for β-actin to ensure equal protein loading. The parent compound Res (50 µM) served as the positive control. Data are presented as mean ± SD (n = 3). Significance is indicated as $^{*}p < 0.05$, $^{**}p < 0.01$, $^{***}p < 0.001$ compared to untreated control cells, and $^{\#}p < 0.05$, $^{\#\#}p < 0.01$, $^{\#\#\#}p < 0.001$ compared to Res-treated H460 cells.

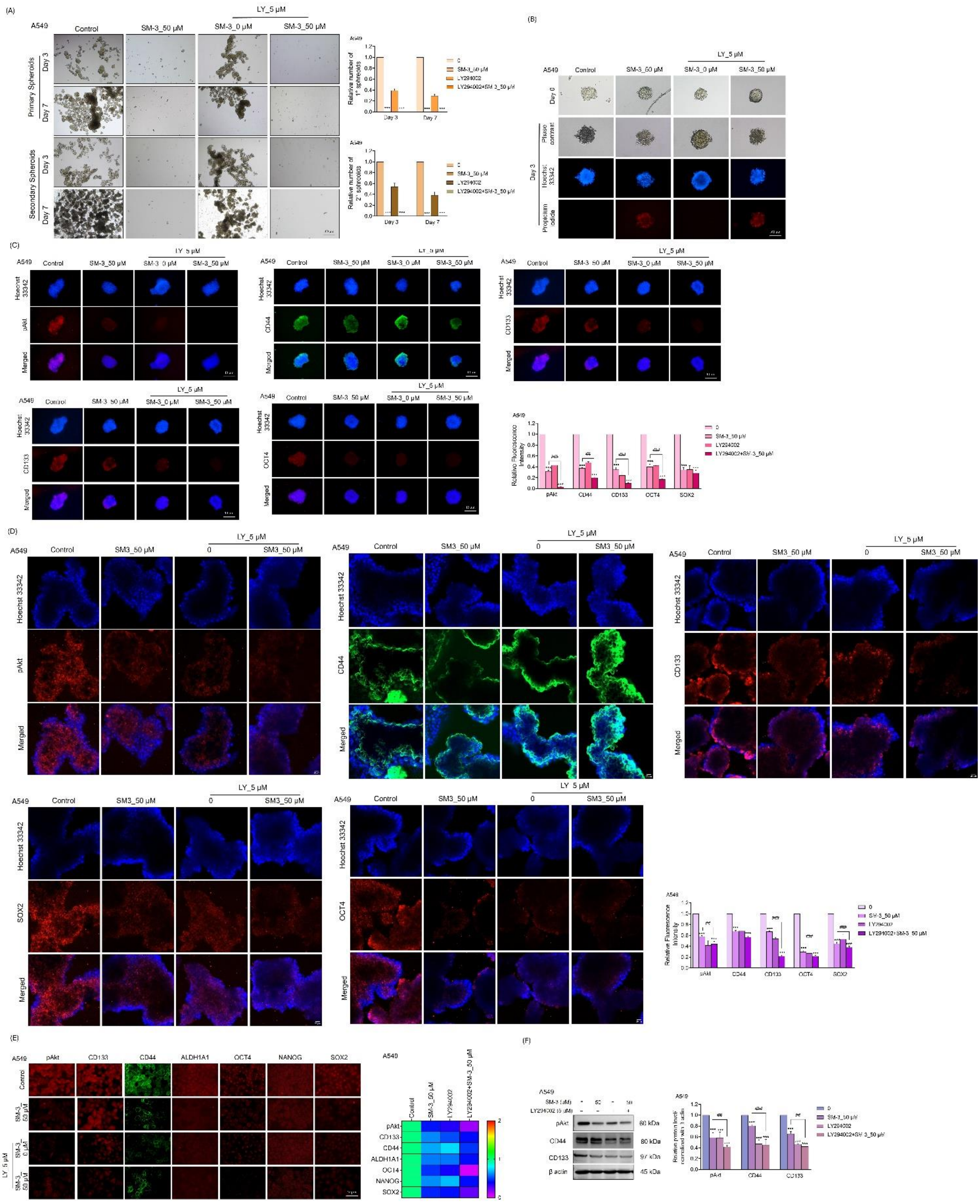

Figure S5. Suppressive effects of SM-3 on CSCs markers via Akt inhibition (LY294002) during spheroids and organoids formation in A549 cells. (A) The A549 cells were pretreated with LY294002 (5 μM) for 1h with SM-3 (50 μM) for 24h and observed using a phase-contrast microscope after 3 and 7 days of incubated primary and secondary spheroids. The relative number of spheroids were analyzed using

ImageJ software. Scale bar: 100 µm (magnification: 4x) (B) Single spheroids from a CSCs-rich population of A549 cells were treated with LY294002 with SM-3 (50 µM) and SM-3 alone for 3 days, and CSCs viability was evaluated using Hoechst 33342/PI double staining. Scale bar: 50 µm (magnification: 20x) (C) The levels of upstream proteins pAkt in LY294002 with SM-3 treated CSCs-rich spheroids were assessed using immunofluorescence. The LY294002 with SM-3 treated A549 spheroids were examined for the expression of stem cell markers, as well as the transcription factors. Scale bar: 50 µm (magnification: 20x) (D) SM-3 with Akt inhibitor (LY294002) targeted CSCs rich 3D organoids formation in A549 cells, as analyzed by immunofluorescence. The expression levels of pAkt (Ser473), CSCs-rich transcription factors (CD133 and CD44), and stem cell markers (OCT4 and SOX2) were measured in LY294002 with SM-3-treated organoids. Scale bar: 20 µm (magnification: 40x) by confocal microscope (E) The heat map displays the fluorescence intensity of stem cell markers and stem cell transcription factors analyzed by using Image J software. Scale bar: 10 µm (magnification: 40x) (F) The protein expression levels of stem cell markers were assessed using western blot analysis, with the blot reprobed for β-actin to ensure equal protein loading. Data are presented as mean ± SD (n = 3). Significance is indicated as $^{*}p < 0.05$, $^{**}p < 0.01$, $^{***}p < 0.001$ compared to untreated control cells, and $^{\#}p < 0.05$, $^{\#\#}p < 0.01$, $^{\#\#\#}p < 0.001$ compared to SM-3 alone.

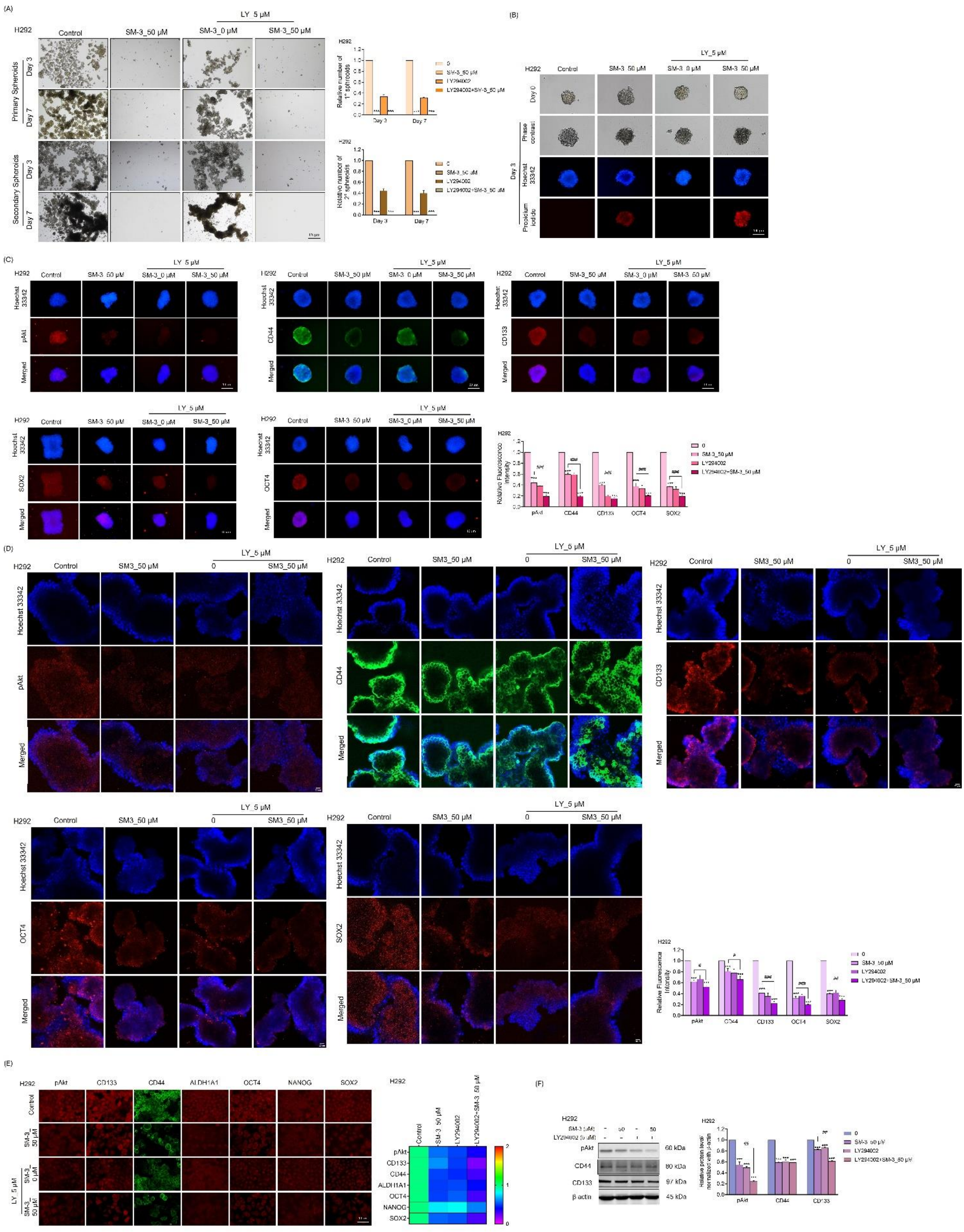

Figure S6. Suppressive effects of SM-3 on CSCs markers via Akt inhibition (LY294002) during spheroids and organoids formation in H292 cells. (A) The H292 cells were pretreated with LY294002 (5 μM) for

1h with SM-3 (50 µM) for 24h and observed using a phase-contrast microscope after 3 and 7 days of incubated primary and secondary spheroids. The relative number of spheroids were analyzed using ImageJ software. Scale bar: 100 µm (magnification: 4x) (B) Single spheroids from a CSCs-rich population of H292 cells were treated with LY294002 with SM-3 (50 µM) and SM-3 alone for 3 days, and CSCs viability was evaluated using Hoechst 33342/PI double staining. Scale bar: 50 µm (magnification: 20x) (C) The levels of upstream proteins pAkt in LY294002 with SM-3 treated CSCs-rich spheroids were assessed using immunofluorescence. The LY294002 with SM-3 treated H292 spheroids were examined for the expression of stem cell markers, as well as the transcription factors. Scale bar: 50 µm (magnification: 20x) (D) SM-3 with Akt inhibitor (LY294002) targeted CSCs rich 3D organoids formation in H292 cells, as analyzed by immunofluorescence. The expression levels of pAkt (Ser473), CSCs-rich transcription factors (CD133 and CD44), and stem cell markers (OCT4 and SOX2) were measured in LY294002 with SM-3-treated organoids. Scale bar: 20 µm (magnification: 40x) by confocal microscope (E) The heat map displays the fluorescence intensity of stem cell markers and stem cell transcription factors analyzed by using Image J software. Scale bar: 10 µm (magnification: 40x) (F) The protein expression levels of stem cell markers were assessed using western blot analysis, with the blot reprobed for β-actin to ensure equal protein loading. Data are presented as mean ± SD (n = 3). Significance is indicated as $^{*}p < 0.05$, $^{**}p < 0.01$, $^{***}p < 0.001$ compared to untreated control cells, and $^{\#}p < 0.05$, $^{\#\#}p < 0.01$, $^{\#\#\#}p < 0.001$ compared to SM-3 alone.

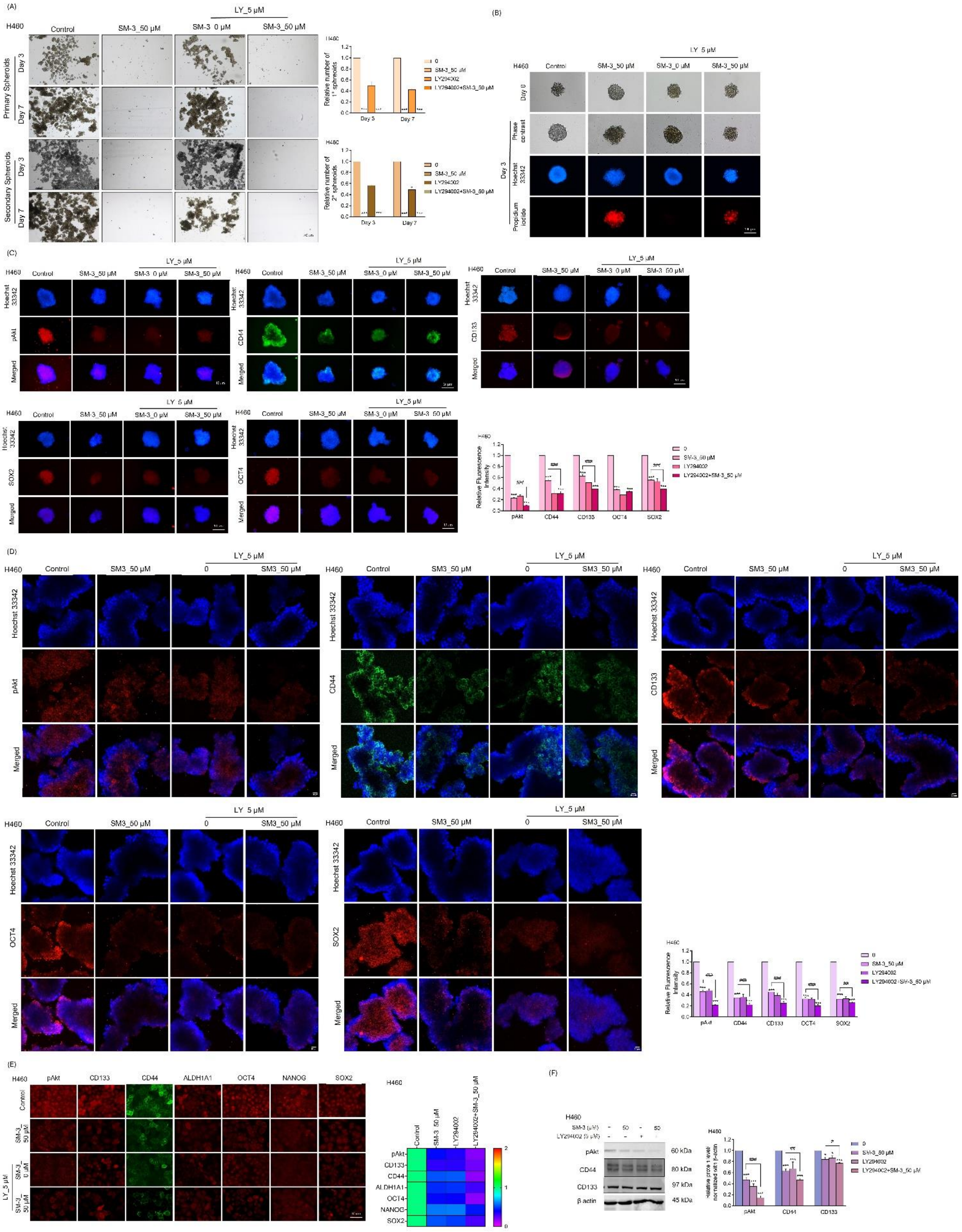

Figure S7. Suppressive effects of SM-3 on CSCs markers via Akt inhibition (LY294002) during spheroids and organoids formation in H460 cells. (A) The H460 cells were pretreated with LY294002 (5 μM) for

1h with SM-3 (50 µM) for 24h and observed using a phase-contrast microscope after 3 and 7 days of incubated primary and secondary spheroids. The relative number of spheroids were analyzed using ImageJ software. Scale bar: 100 µm (magnification: 4x) (B) Single spheroids from a CSCs-rich population of H460 cells were treated with LY294002 with SM-3 (50 µM) and SM-3 alone for 3 days, and CSCs viability was evaluated using Hoechst 33342/PI double staining. Scale bar: 50 µm (magnification: 20x) (C) The levels of upstream proteins pAkt in LY294002 with SM-3 treated CSCs-rich spheroids were assessed using immunofluorescence. The LY294002 with SM-3 treated H460 spheroids were examined for the expression of stem cell markers, as well as the transcription factors. Scale bar: 50 µm (magnification: 20x) (D) SM-3 with Akt inhibitor (LY294002) targeted CSCs rich 3D organoids formation in H460 cells, as analyzed by immunofluorescence. The expression levels of pAkt (Ser473), CSCs-rich transcription factors (CD133 and CD44), and stem cell markers (OCT4 and SOX2) were measured in LY294002 with SM-3-treated organoids. Scale bar: 20 µm (magnification: 40x) by confocal microscope (E) The heat map displays the fluorescence intensity of stem cell markers and stem cell transcription factors analyzed by using Image J software. Scale bar: 10 µm (magnification: 40x) (F) The protein expression levels of stem cell markers were assessed using western blot analysis, with the blot reprobed for β-actin to ensure equal protein loading. Data are presented as mean ± SD (n = 3). Significance is indicated as $^{*}p < 0.05$, $^{**}p < 0.01$, $^{***}p < 0.001$ compared to untreated control cells, and $^{\#}p < 0.05$, $^{\#\#}p < 0.01$, $^{\#\#\#}p < 0.001$ compared to SM-3 alone.

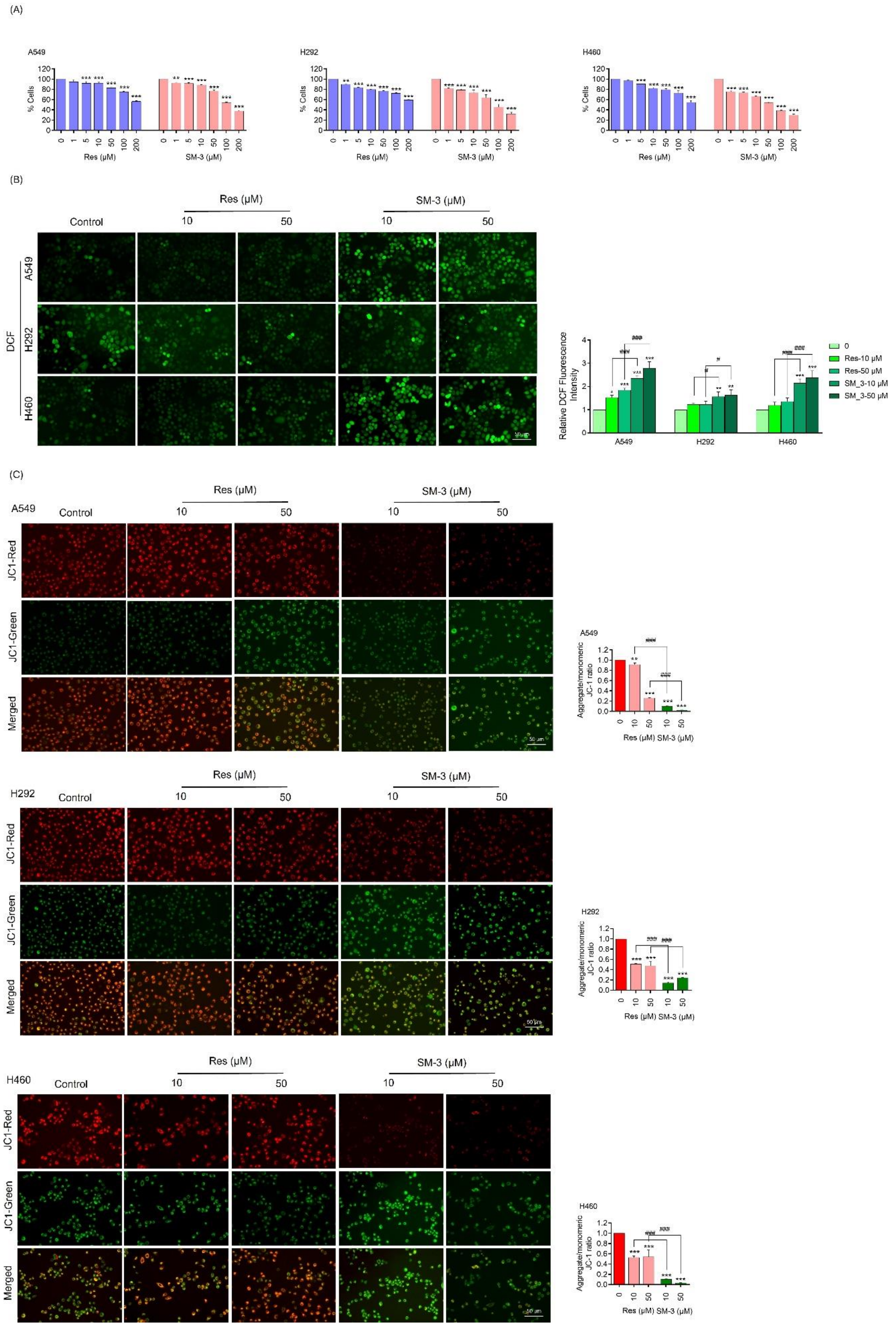

Figure S8. Effects of Res and SM-3 on mitochondria function and integrity on NSCLC cells. (A) NSCLCs

cells were treated with various concentrations of Res or SM-3 (0-200 µM) for 24 h. The resazurin reduction assay was determined for mitochondrial metabolic activity of Res and SM-3 treated NSCLC cells. (B) Lung cancer cells were treated with various concentrations of Res or SM-3 (0-50 µM). The intracellular ROS production of Res or SM-3 treated lung cancer cells were detected by fluorescence microscopy using DCFH2-DA probe. (C) Detection of mitochondrial membrane potential by JC-1 staining after Res or SM-3 treatment on lung cancer cells. Quantitative analysis for aggregate (red)/monomeric (green) ratio for SM-3 treated lung cancer cells compared to Res treated population. Data are presented as mean ± SD (n = 3), with significance indicated as $^{*}p < 0.05$, $^{**}p < 0.01$, and $^{***}p < 0.001$ compared to untreated control cells, $^{\#}p < 0.05$, $^{\#\#}p < 0.01$, $^{\#\#\#}p < 0.001$ compared to Res-treated NSCLC cells.

Uncropped blotting bands for western blot analysis

Figure 8 (B)

A549

mTOR = 289 kDa

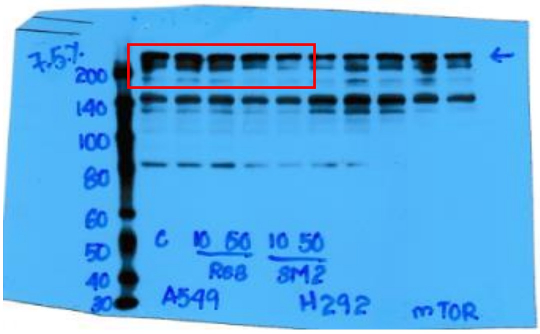

pmTOR= 289 kDa

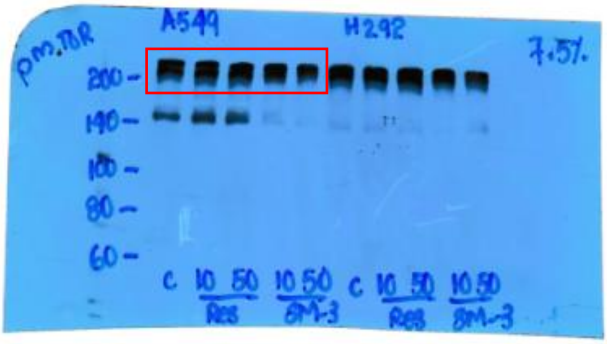

Akt = 60 kDa

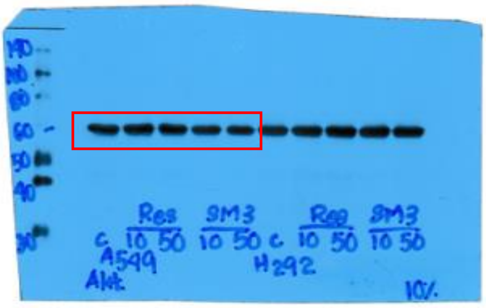

pAkt= 60 kDa

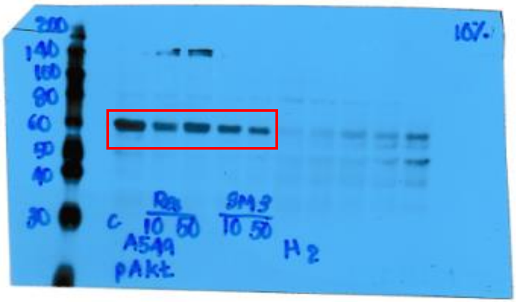

β-actin = 45 kDa

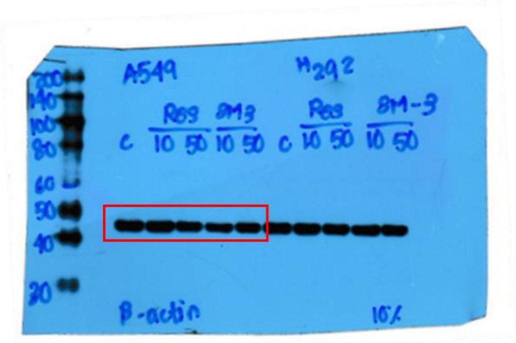

Figure 8 (B)

H292

mTOR = 289 kDa

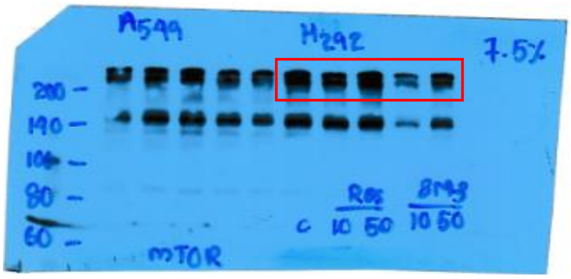

pmTOR= 289 kDa

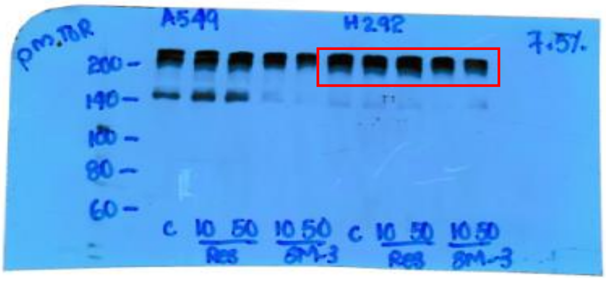

Akt = 60 kDa

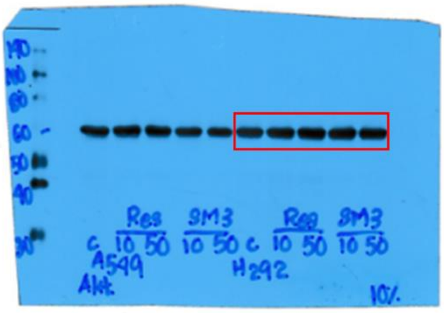

pAkt= 60 kDa

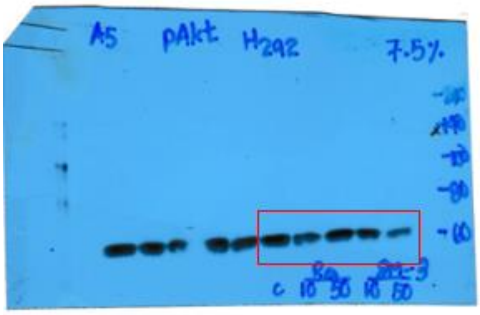

β-actin = 45 kDa

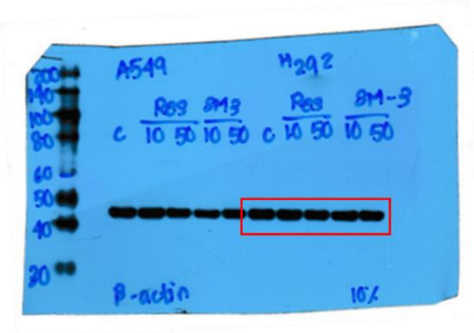

Figure 8 (B)

H460

mTOR = 289 kDa

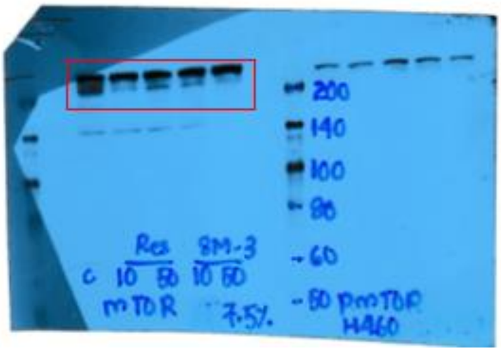

pmTOR= 289 kDa

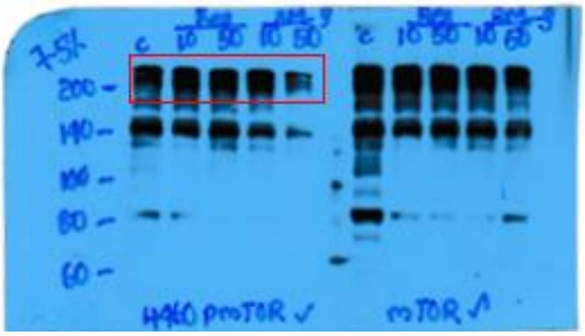

Akt = 60 kDa

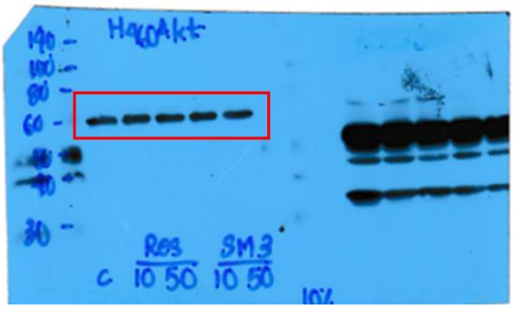

pAkt= 60 kDa

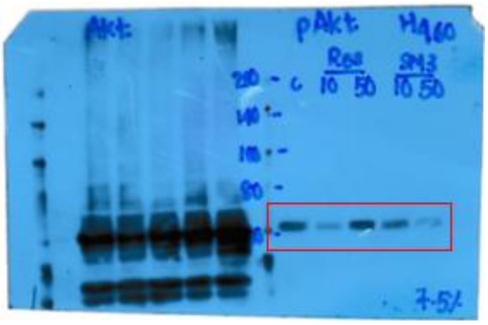

β-actin = 45 kDa

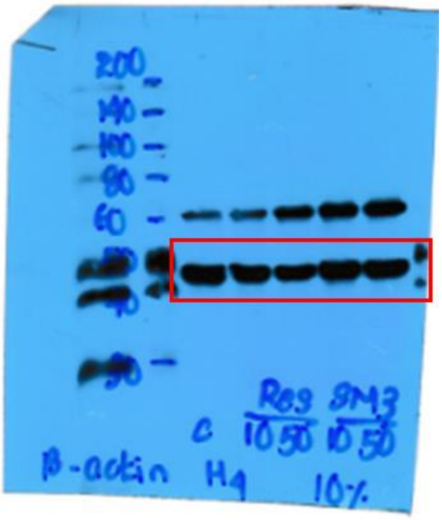

Figure 9 (I)

A549

CD44 = 80 kDa

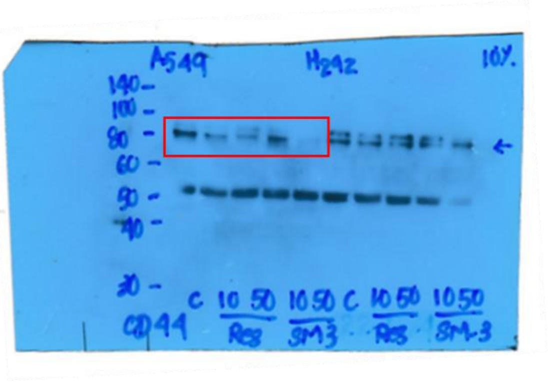

CD133= 97 kDa

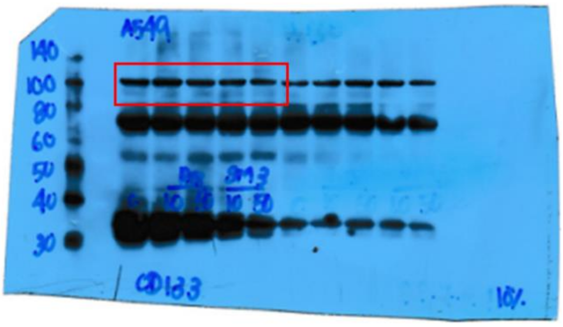

β-actin = 45 kDa

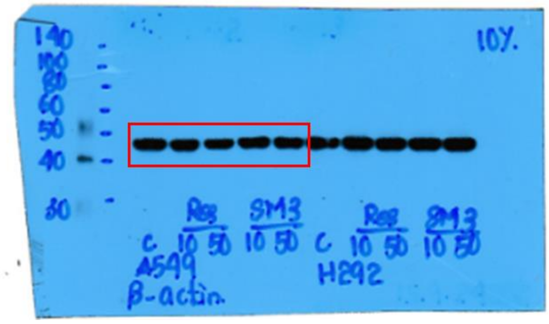

Figure 10 (B)

A549

LC3BI/II = 16, 14 kDa

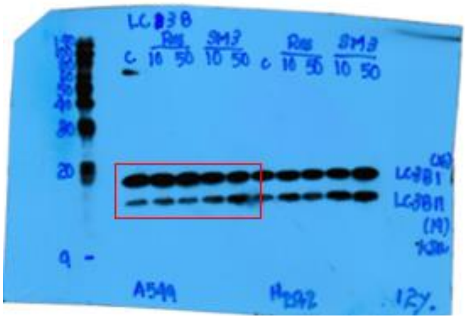

p62= 62 kDa

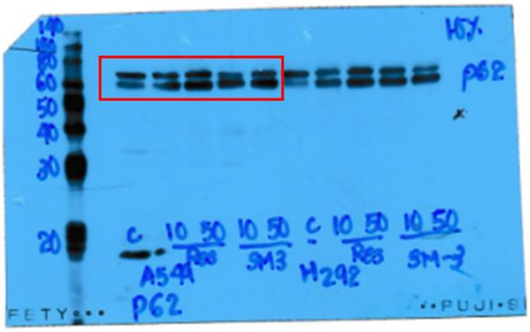

ATG7 = 78 kDa

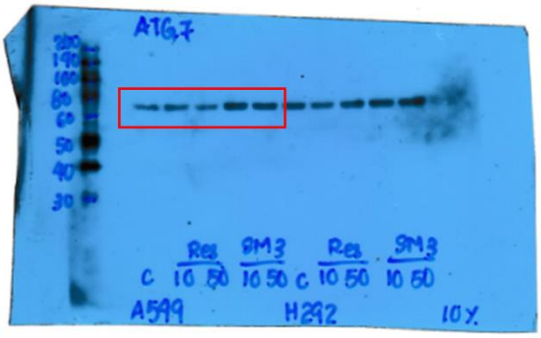

ATG5= 55 kDa

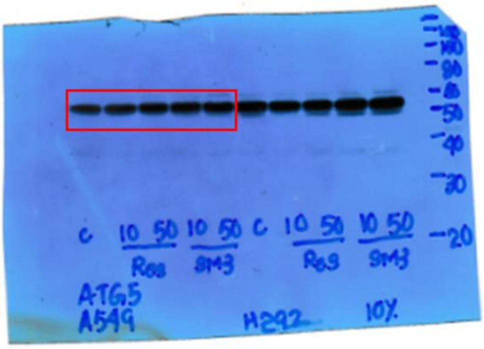

β-actin = 45 kDa

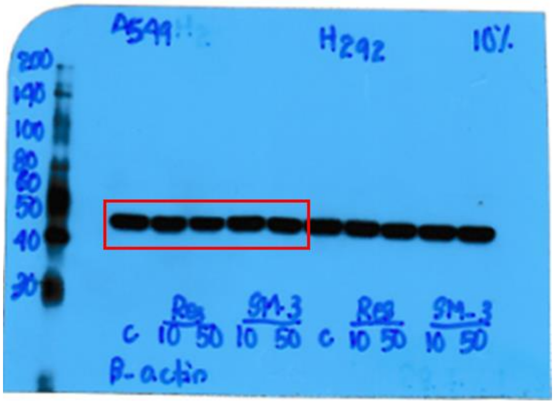

Figure 10 (B)

H292

LC3BI/II = 16, 14 kDa

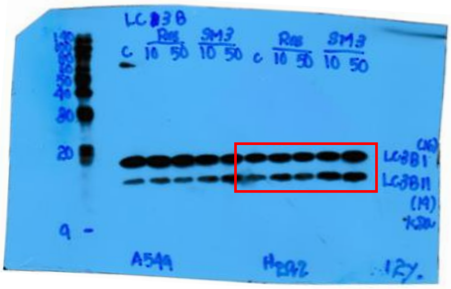

p62= 62 kDa

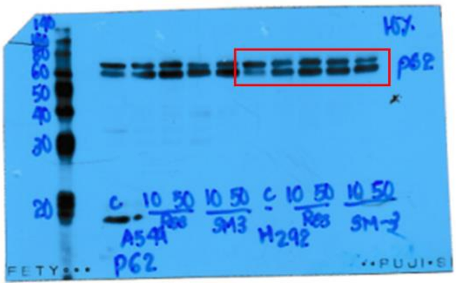

ATG7 = 78 kDa

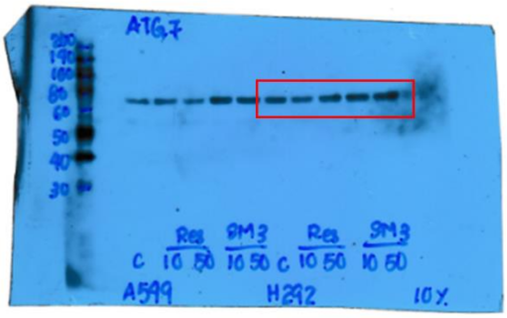

ATG5= 55 kDa

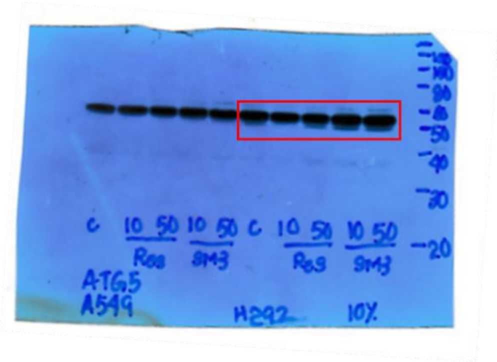

β-actin = 45 kDa

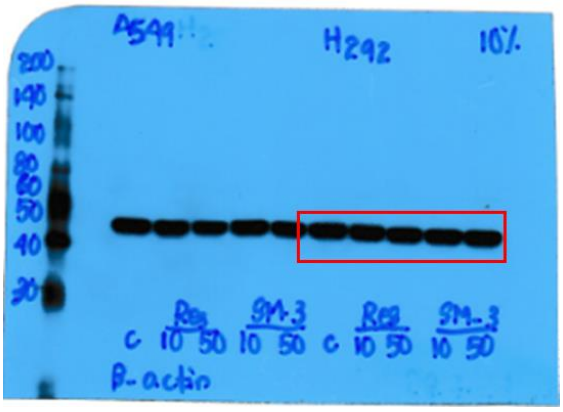

Figure 10 (B)

H460

LC3BI/II = 16, 14 kDa

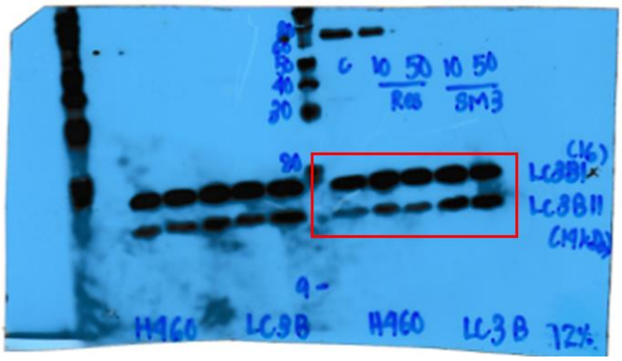

p62= 62 kDa

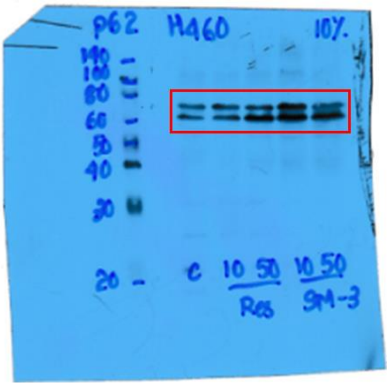

ATG7 = 78 kDa

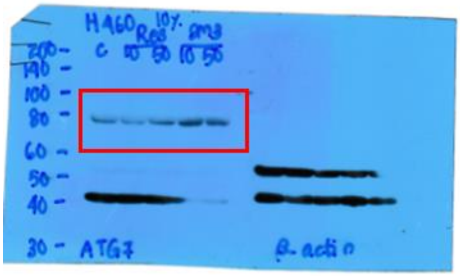

ATG5= 55 kDa

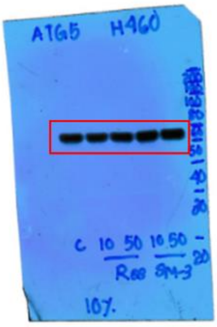

β-actin = 45 kDa

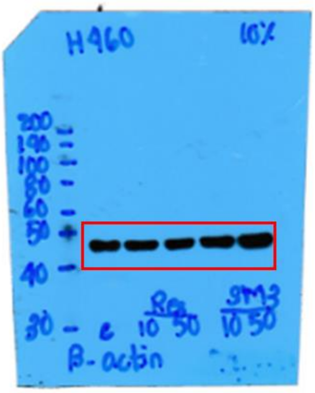

Figure S3

H292

CD44 = 80 kDa

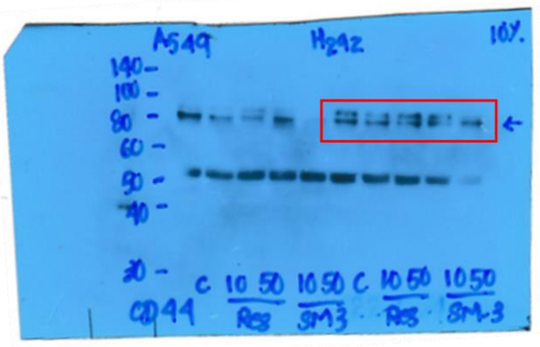

CD133= 97 kDa

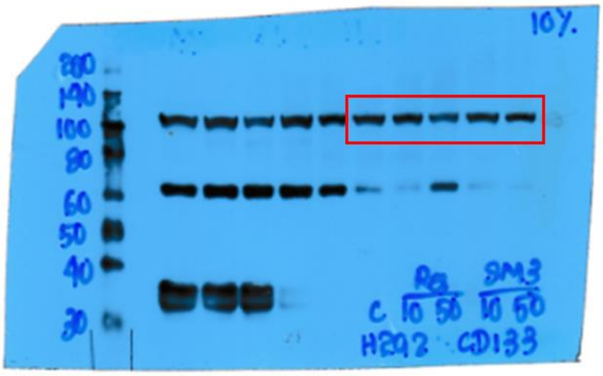

β-actin = 45 kDa

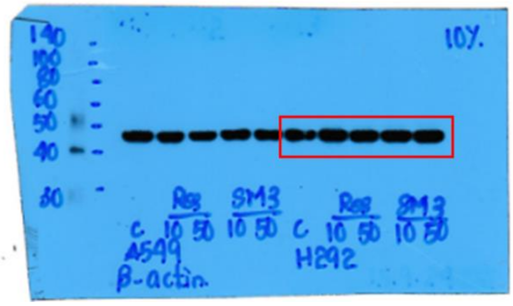

Figure S4

H460

CD44 = 80 kDa

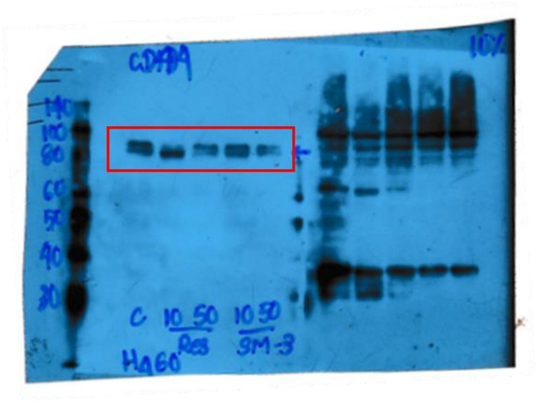

CD133= 97 kDa

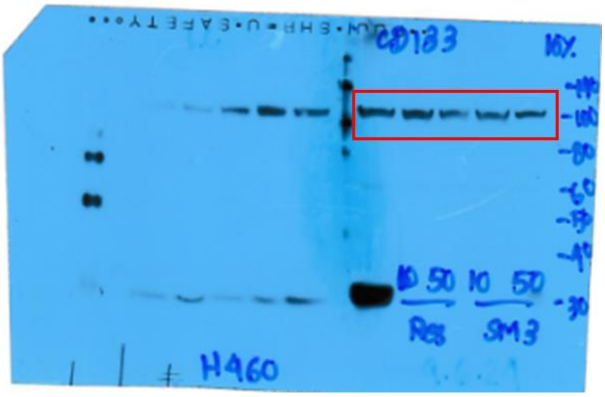

β-actin = 45 kDa

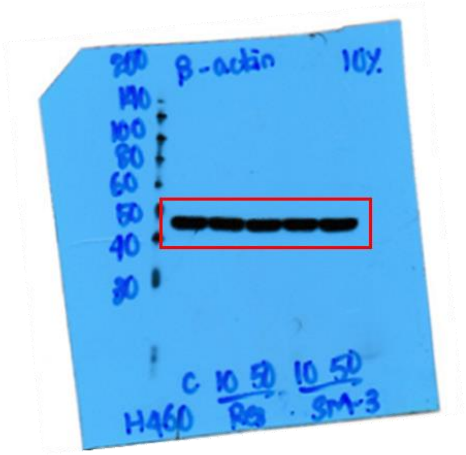

Figure S2 (C)
A549
mTOR = 289 kDa

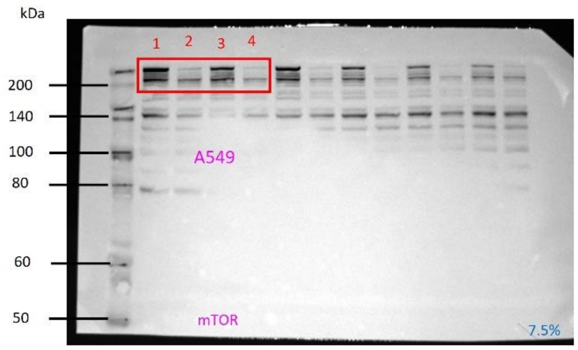

1. Ctrl
2. SM-3 (50 μM)
3. Rapamycin (0.2 μM)
4. Rapamycin (0.2 μM)+ SM-3 (50 μM)

pmTOR = 289 kDa

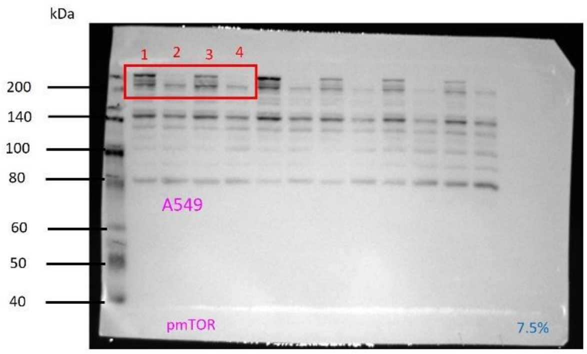

1. Ctrl
2. SM-3 (50 μM)
3. Rapamycin (0.2 μM)
4. Rapamycin (0.2 μM)+ SM-3 (50 μM)

β-actin = 45 kDa

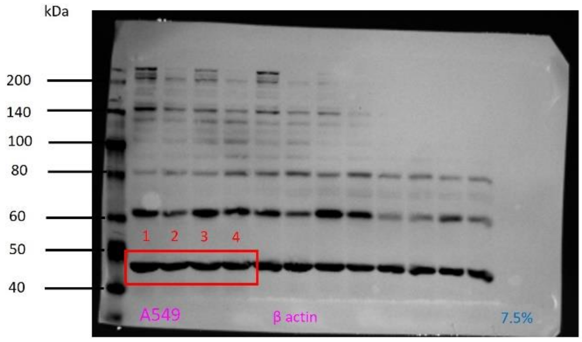

1. Ctrl
2. SM-3 (50 μM)
3. Rapamycin (0.2 μM)
4. Rapamycin (0.2 μM)+ SM-3 (50 μM)

Figure S2 (C)
H292
mTOR = 289 kDa

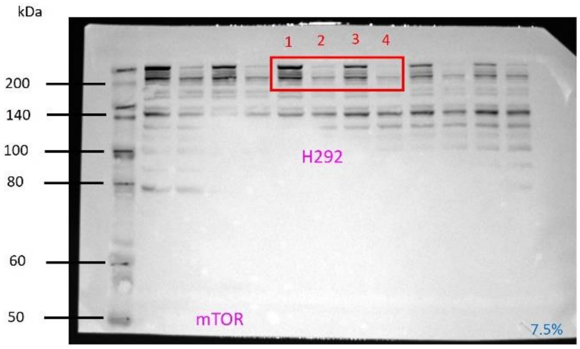

1. Ctrl
2. SM-3 (50 μM)
3. Rapamycin (0.2 μM)
4. Rapamycin (0.2 μM)+ SM-3 (50 μM)

pmTOR = 289 kDa

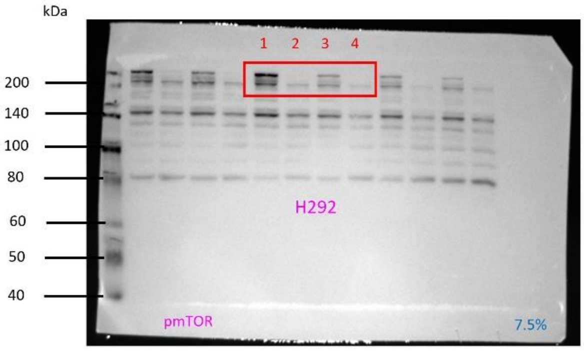

1. Ctrl
2. SM-3 (50 μM)
3. Rapamycin (0.2 μM)
4. Rapamycin (0.2 μM)+ SM-3 (50 μM)

β-actin = 45 kDa

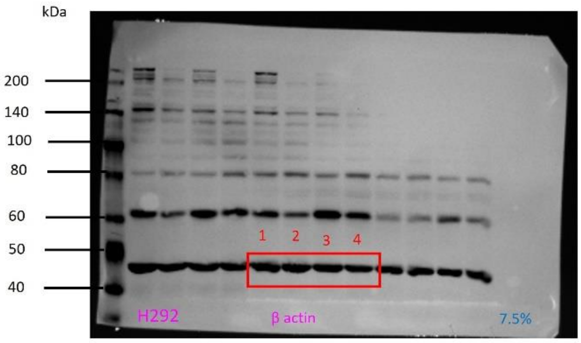

1. Ctrl
2. SM-3 (50 μM)
3. Rapamycin (0.2 μM)
4. Rapamycin (0.2 μM)+ SM-3 (50 μM)

Figure S2 (C)
H460
mTOR = 289 kDa

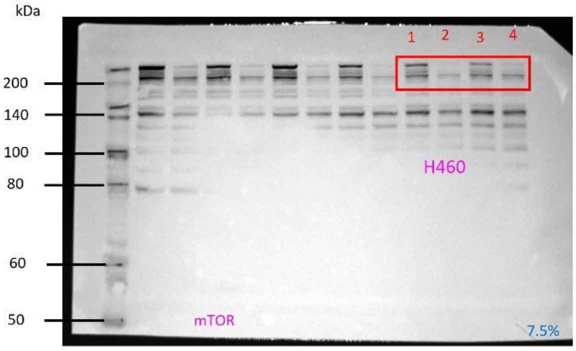

1. Ctrl
2. SM-3 (50 µM)
3. Rapamycin (0.2 µM)
4. Rapamycin (0.2 µM)+ SM-3 (50 µM)

pmTOR = 289 kDa

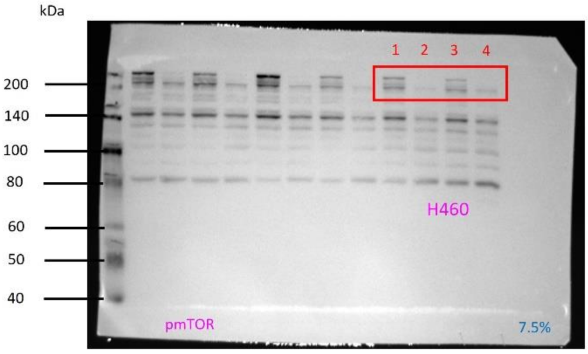

1. Ctrl
2. SM-3 (50 µM)
3. Rapamycin (0.2 µM)
4. Rapamycin (0.2 µM)+ SM-3 (50 µM)

β-actin = 45 kDa

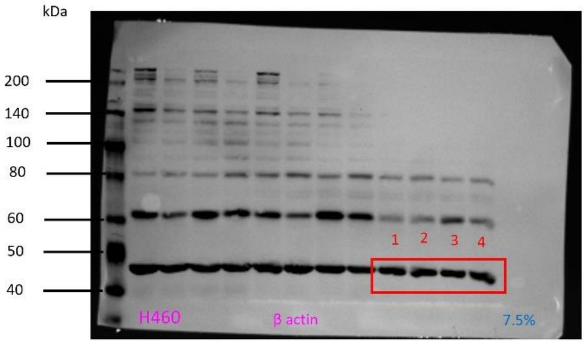

1. Ctrl
2. SM-3 (50 µM)
3. Rapamycin (0.2 µM)
4. Rapamycin (0.2 µM)+ SM-3 (50 µM)

Figure S5 (F)
A549
pAkt = 60 kDa

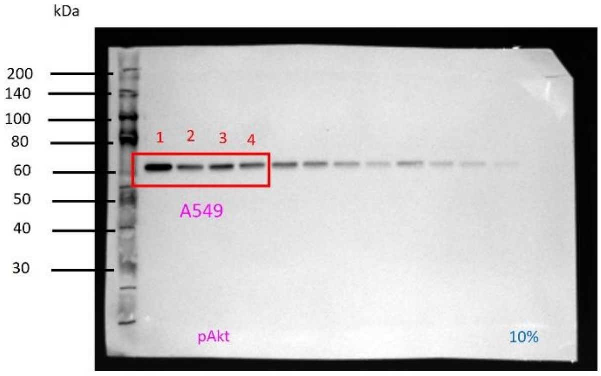

1. Ctrl
2. SM-3 (50 µM)
3. LY294002 (5 µM)
4. LY294002 (5 µM)+ SM-3 (50 µM)

CD44 = 80 kDa

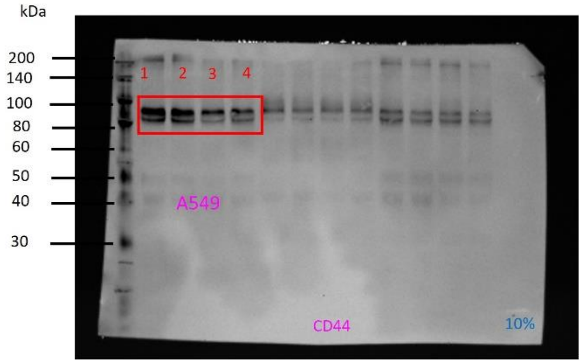

1. Ctrl
2. SM-3 (50 µM)
3. LY294002 (5 µM)
4. LY294002 (5 µM)+ SM-3 (50 µM)

CD133 = 97 kDa

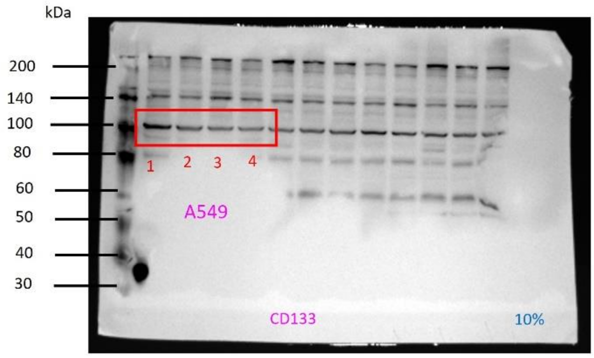

1. Ctrl
2. SM-3 (50 µM)
3. LY294002 (5 µM)
4. LY294002 (5 µM)+ SM-3 (50 µM)

β-actin = 45 kDa

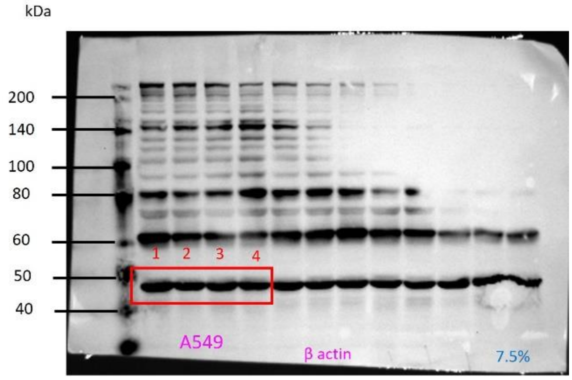

1. Ctrl
2. SM-3 (50 µM)
3. LY294002 (5 µM)
4. LY294002 (5 µM)+ SM-3 (50 µM)

Figure S6 (F)
H292
pAkt = 60 kDa

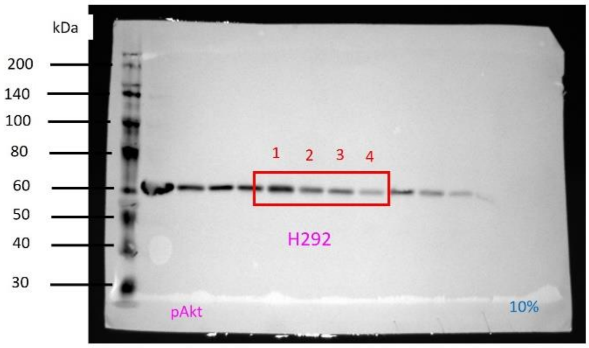

1. Ctrl
2. SM-3 (50 μM)
3. LY294002 (5 μM)
4. LY294002 (5 μM)+ SM-3 (50 μM)

CD44 = 80 kDa

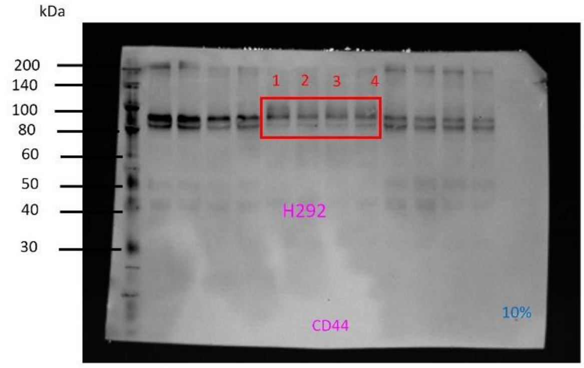

1. Ctrl
2. SM-3 (50 μM)
3. LY294002 (5 μM)
4. LY294002 (5 μM)+ SM-3 (50 μM)

CD133 = 97 kDa

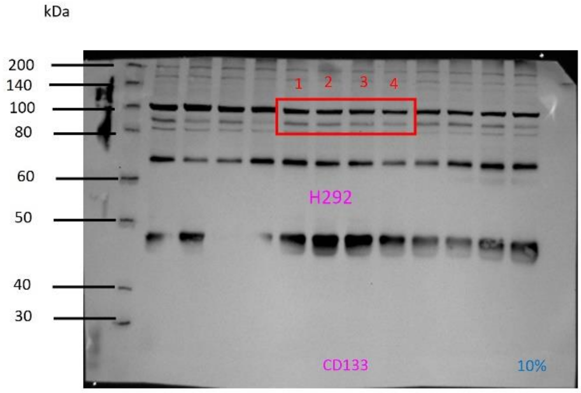

1. Ctrl
2. SM-3 (50 μM)
3. LY294002 (5 μM)
4. LY294002 (5 μM)+ SM-3 (50 μM)

β-actin = 45 kDa

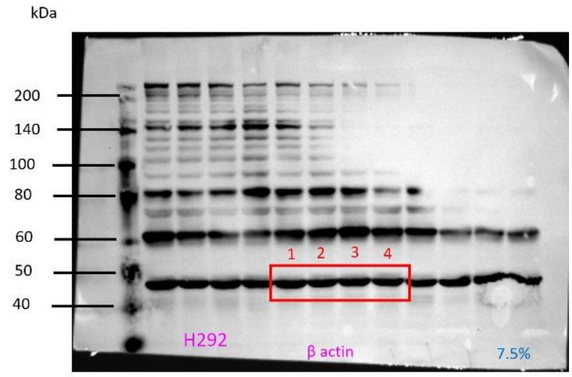

1. Ctrl
2. SM-3 (50 μM)
3. LY294002 (5 μM)
4. LY294002 (5 μM)+ SM-3 (50 μM)

Figure S7 (F)
H460
pAkt = 60 kDa

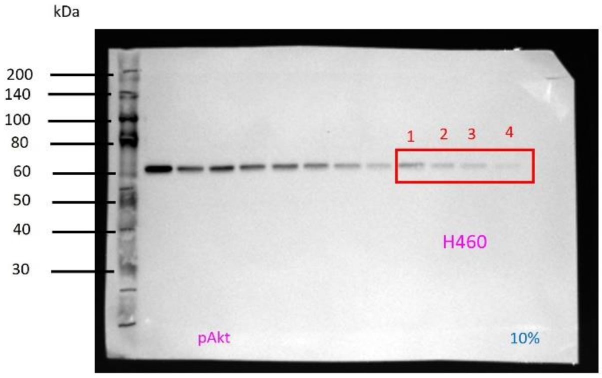

1. Ctrl
2. SM-3 (50 µM)
3. LY294002 (5 µM)
4. LY294002 (5 µM)+ SM-3 (50 µM)

CD44 = 80 kDa

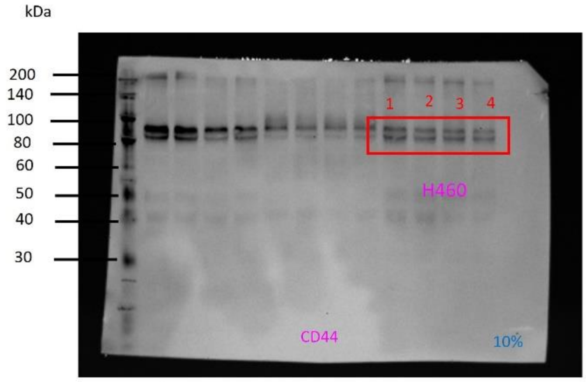

1. Ctrl
2. SM-3 (50 µM)
3. LY294002 (5 µM)
4. LY294002 (5 µM)+ SM-3 (50 µM)

CD133 = 97 kDa

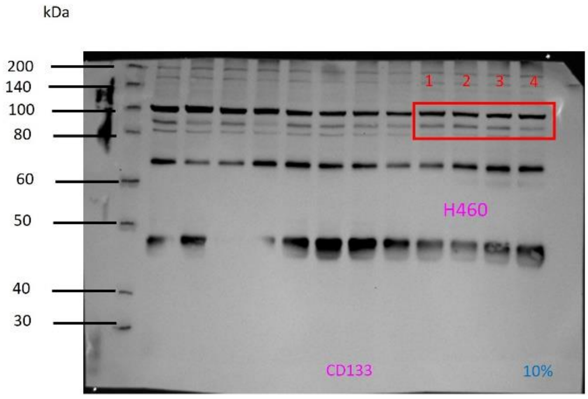

1. Ctrl
2. SM-3 (50 µM)
3. LY294002 (5 µM)
4. LY294002 (5 µM)+ SM-3 (50 µM)

β-actin = 45 kDa

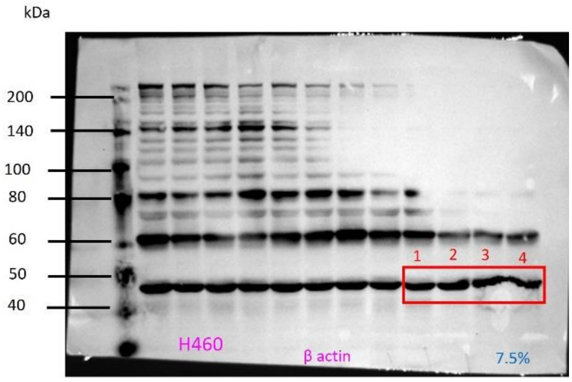

1. Ctrl
2. SM-3 (50 µM)
3. LY294002 (5 µM)
4. LY294002 (5 µM)+ SM-3 (50 µM)
