# Supplementary figures and images for: Substituents introduction of methyl and methoxy functional groups on resveratrol stabilizes mTOR binding for autophagic cell death induction
Source: Sci Rep. 2025 Apr 26;15:14675. doi: 10.1038/s41598-025-98616-6 (PMC12033263; doi:10.1038/s41598-025-98616-6)

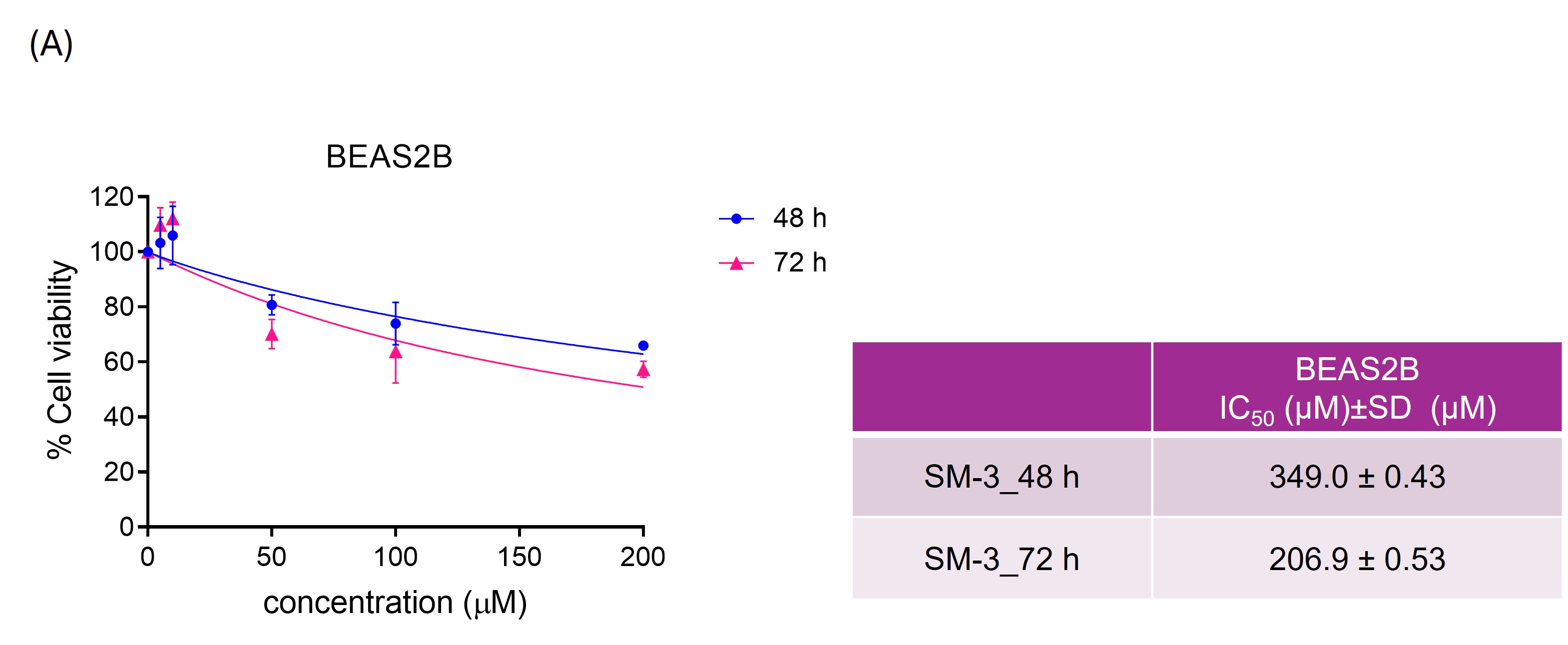

Supplement: Supplementary file 2 — Supplementary Material 2 [file 41598_2025_98616_MOESM2_ESM.jpg]

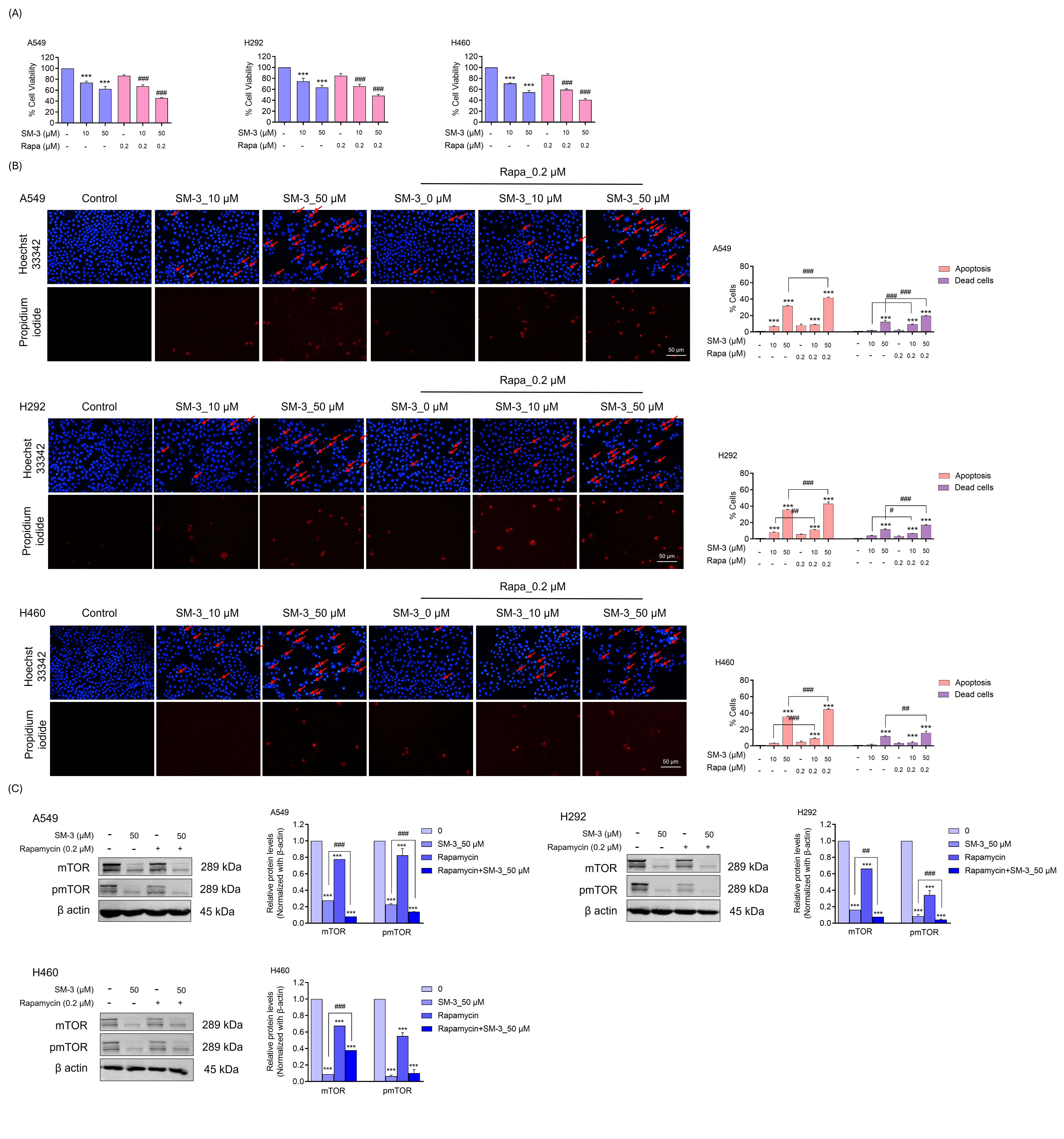

Supplement: Supplementary file 3 — Supplementary Material 3 [file 41598_2025_98616_MOESM3_ESM.jpg]

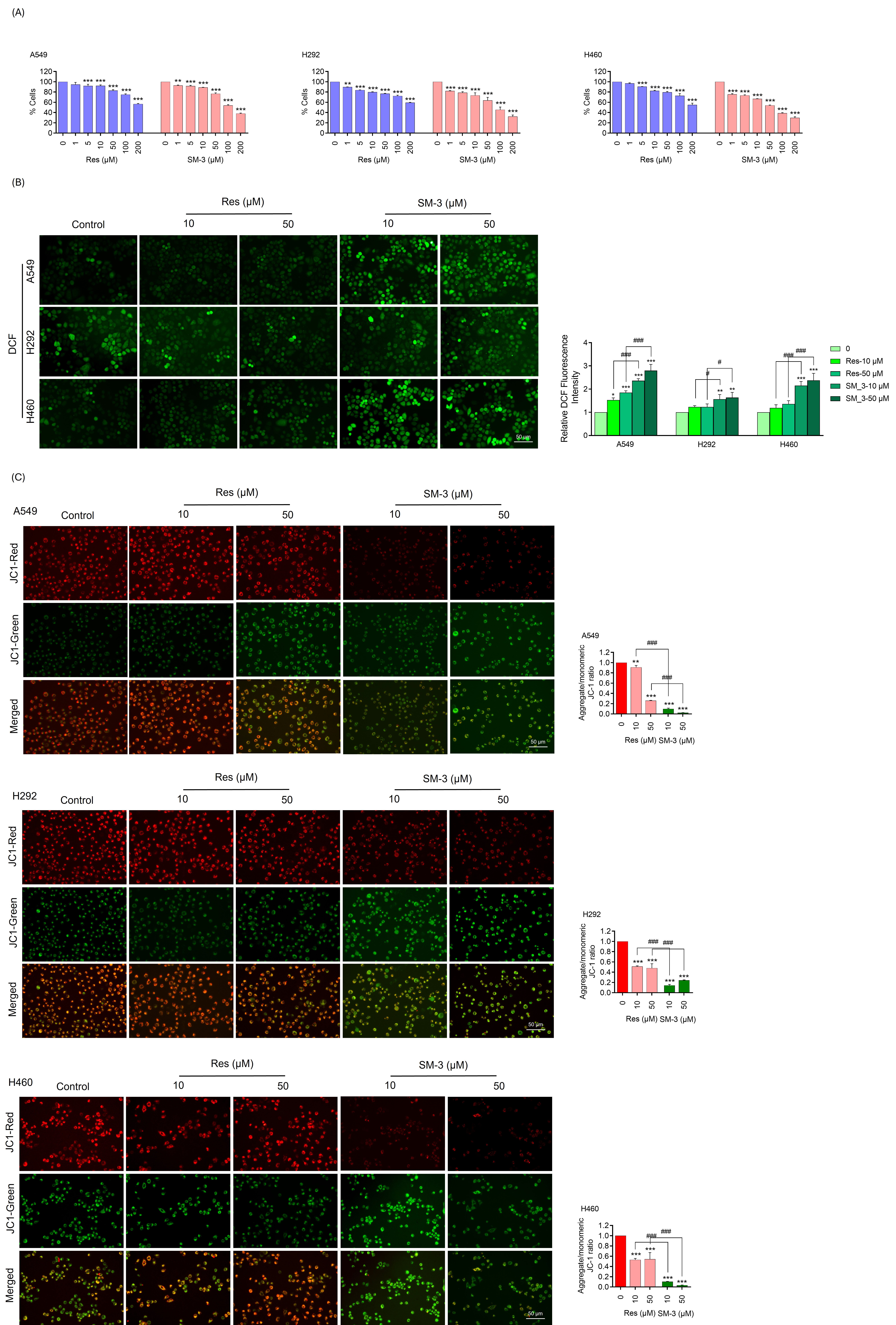

Supplement: Supplementary file 9 — Supplementary Material 9 [file 41598_2025_98616_MOESM9_ESM.jpg]
